# Human model of primary carnitine deficiency cardiomyopathy reveals ferroptosis as a novel mechanism

**DOI:** 10.1016/j.stemcr.2023.09.002

**Published:** 2023-10-05

**Authors:** Malte Loos, Birgit Klampe, Thomas Schulze, Xiaoke Yin, Konstantinos Theofilatos, Bärbel Maria Ulmer, Carl Schulz, Charlotta S. Behrens, Tessa Diana van Bergen, Eleonora Adami, Henrike Maatz, Michaela Schweizer, Susanne Brodesser, Boris V. Skryabin, Timofey S. Rozhdestvensky, Sara Bodbin, Konstantina Stathopoulou, Torsten Christ, Chris Denning, Norbert Hübner, Manuel Mayr, Friederike Cuello, Thomas Eschenhagen, Arne Hansen

**Affiliations:** 1 University Medical Center Hamburg-Eppendorf, Department of Experimental Pharmacology and Toxicology, 20246 Hamburg, Germany; 2 German Center for Heart Research (DZHK), Partner site Hamburg/Lübeck/Kiel, 20246 Hamburg, Germany; 3 King’s British Heart Foundation Centre of Research Excellence, King’s College London, London, UK; 4 Cardiovascular and Metabolic Sciences, Max Delbrück Center for Molecular Medicine in the Helmholtz Association (MDC), 13125 Berlin, Germany; 5 Electron Microscopy Unit, Center for Molecular Neurobiology Hamburg, University Medical Center Hamburg-Eppendorf, 20251 Hamburg, Germany; 6 Cluster of Excellence Cellular Stress Responses in Aging-associated Diseases (CECAD), Faculty of Medicine and University Hospital of Cologne, 50931 Cologne, Germany; 7 Transgenic animal and genetic engineering Models (TRAM), Faculty of Medicine of the Westfalian Wilhelms-University, 48149 Muenster, Germany; 8 Division of Cancer & Stem Cells, Biodiscovery Institute, University of Nottingham, NG7 2RD Nottingham, UK; 9 DZHK (German Centre for Cardiovascular Research), Partner Site Berlin, 13347 Berlin, Germany; 10 Charité-Universitätsmedizin, 10117 Berlin, Germany

**Keywords:** disease modeling, Metabolism, Dilated Cardiomyopathy, iPSC, Cardiomyocytes

## Abstract

Primary carnitine deficiency (PCD) is an autosomal recessive monogenic disorder caused by mutations in *SLC22A5*. This gene encodes for OCTN2, which transports the essential metabolite carnitine into the cell. PCD patients suffer from muscular weakness and dilated cardiomyopathy. Two OCTN2-defective human induced pluripotent stem cell lines were generated, carrying a full OCTN2 knockout and a homozygous OCTN2 (N32S) loss-of-function mutation. OCTN2-defective genotypes showed lower force development and resting length in engineered heart tissue format compared with isogenic control. Force was sensitive to fatty acid-based media and associated with lipid accumulation, mitochondrial alteration, higher glucose uptake, and metabolic remodeling, replicating findings in animal models. The concordant results of OCTN2 (N32S) and -knockout emphasizes the relevance of OCTN2 for these findings. Importantly, genome-wide analysis and pharmacological inhibitor experiments identified ferroptosis, an iron- and lipid-dependent cell death pathway associated with fibroblast activation as a novel PCD cardiomyopathy disease mechanism.

## Highlights

• Modeling of primary carnitine deficiency cardiomyopathy in hiPSC cardiomyocytes
• Demonstration of low force and metabolic remodeling as key disease phenotype
• Identification of ferroptosis and fibroblast activation as novel disease mechanisms

## Graphical abstract

**Figure:**
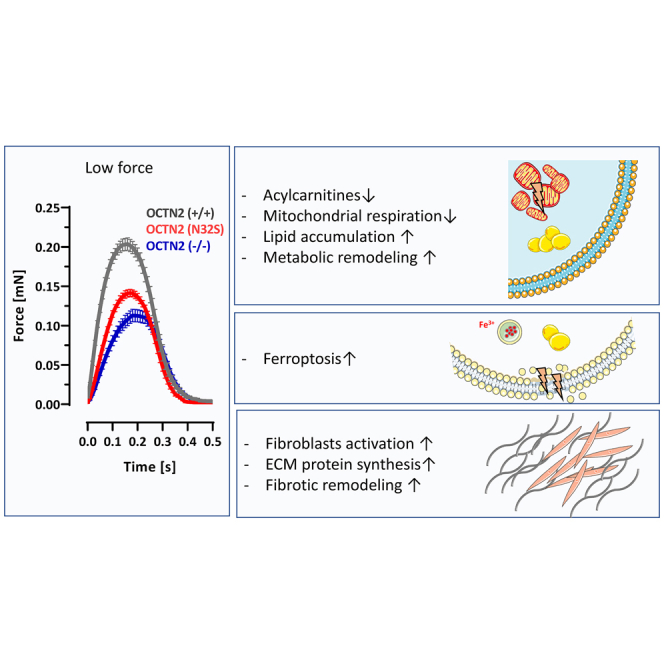

## Teaser

Primary carnitine deficiency (PCD) caused by mutations in *SLC22A5* lead to disturbed carnitine transport into mitochondria associated with muscular weakness and dilated cardiomyopathy (DCM). In this study, Hansen and colleagues show that PCD DCM modeled in human induced pluripotent stem cell cardiomyocytes reveal lower force and metabolic remodeling. Ferroptosis and fibroblast activation were identified as novel contributing mechanisms.

## Introduction

Primary carnitine deficiency (PCD) is an autosomal recessive disorder resulting in insufficient cellular carnitine (β-hydroxy-γ-trimethylammonium butyrate) uptake and low cytoplasmic concentrations (Longo et al., 2016). PCD is caused by pathogenic variants of *SLC22A5* (NC_000005.10), leading to loss of function of the encoded organic cation transporter novel family member 2 (OCTN2). OCTN2 is strongly expressed in the myocardium, skeletal muscle, fibroblasts, renal tubules, placenta, and intestine (Wagner et al., 2000). OCTN2 transports carnitine in a sodium-dependent manner and maintains intracellular concentrations 20- to 50-fold higher than extracellular (Tamai et al., 1998). Cytoplasmic carnitine palmitoyltransferase 1 (CPT1) catalyzes acylcarnitine formation. Carnitine-acylcarnitine translocase (CACT) transports acylcarnitine into the mitochondria, where carnitine palmitoyltransferase 2 (CPT2) reconverts acylc-arnitine to acyl-CoA, which subsequently enters beta-oxidation. Low cytoplasmic carnitine concentration impairs fatty acid beta-oxidation and promotes cytoplasmic lipid accumulation. This results in a glucose-dependent energy metabolism, inhibited gluconeogenesis via pyruvate carboxylase inhibition, and diminished ketogenesis via lack of acetyl-CoA (Longo et al., 2016). Dependence on fatty acids renders cardiomyocytes particularly susceptible to PCD pathomechanisms. Carnitine conjugation is also important to reduce coenzyme A (CoA) bound to acyl residues. Cytoplasmic acyl-CoA accumulation results in ceramide, triglycerides, and cholesteryl-ester formation triggering inflammation and apoptosis (Cooper et al., 2015).

PCD symptoms are fasting-mediated hypoglycemia and hypoketonemia, liver and muscular dysfunction, and dilated cardiomyopathy (DCM) (Wang et al., 2014). PCD patients have low plasma carnitine concentrations (0–5 vs. 25–50 μmol/L) (Longo et al., 2006) because carnitine reabsorption in the renal proximal tubules system is impaired (Rasmussen et al., 2014). Patients receive a lifetime oral high dose carnitine supplementation (100–200 mg/kg/day) (Wang et al., 2014). Side effects are vomiting, abdominal cramps, diarrhea, a fishy body odor, and accumulation of atherogenic trimethylamine N-oxide (Alesci et al., 2004; Koeth et al., 2013; Rebouche, 2004). Asymptomatic untreated PCD patients reach adulthood but have an increased risk for sudden cardiac death (Rasmussen et al., 2020). Detailed mechanisms of the PCD DCM remain poorly understood.

PCD is a rare disease with a prevalence of 1:20,000–1:70,000 (United States) (Magoulas and El-Hattab, 2012). The Faroe Islands, a Northern Atlantic archipelago, has a higher prevalence with 1:300 (Rasmussen et al., 2014). The *SLC22A5* c.95A>G (N32S) mutation is the characteristic PCD mutation on the Faroe Islands (Rasmussen et al., 2014). The overall approximate allelic frequency of *SLC22A5* pathogenic variants in the population is 0.5%–1% (Tan et al., 2020).

Juvenile visceral steatosis (JVS) mice represent an animal model of carnitine deficiency (Tomomura et al., 1992). This strain was discovered by coincidence to carry an OCTN2 p.L352R missense mutation (Koizumi et al., 1988). JVS mice exhibit high renal carnitine excretion and tissue lipid accumulation, hypoglycemia, hepatic steatosis, and growth retardation (Horiuchi et al., 1993). Also, JVS mice develop cardiac hypertrophy with accumulation of diacylglycerols and triglycerides (Saburi et al., 2003), lower myocardial ATP content (Asai et al., 2006), and high expression of the pyruvate dehydrogenase (PDH) inhibitor PDH kinase 4 (*PDK4*) (Horiuchi et al., 1999). A pharmacological carnitine deficiency animal model was established by administering the competitive OCTN2 and BBOX1 (γ butyrobetaine hydroxylase) inhibitor N-trimethyl-hydrazine-3-propionateto wild-type rats for 3 weeks. The rats developed increased renal carnitine excretion and hepatic steatosis, upregulation of carnitine shuttle proteins, but no apparent cardiac or skeletal phenotype (Spaniol et al., 2001; Degrace et al., 2004). No human induced pluripotent stem cell (hiPSC) model of PCD has been published so far.

The aim of this study was the development of a hiPSC model of PCD DCM. Two hiPSC lines were generated by CRISPR-Cas9 technology. The lines carry a full OCTN2-knockout (OCTN2 (−/−)) or the homozygous missense Faroe Islands founder mutation (OCTN2 (N32S), *SLC22A5* c.95A>G) (Rasmussen et al., 2014). Cardiomyocytes were differentiated, and the PCD disease phenotype was analyzed in genome-wide, molecular, functional, and morphological assays. Overall, this hiPSC PCD model replicates a wide range of PCD DCM characteristics and unveils ferroptosis linked to fibroblast activation as a novel disease mechanism of PCD.

## Results

### CRISPR-Cas9

A control hiPSC line (OCTN2 (+/+)) was used for the CRISPR-Cas9 engineering and served as isogenic control. The CRISPR-Cas9 strategy is presented in Figures S1A and S1B. Representative Sanger sequencing traces of edited clones are depicted in Figure S1C and show the OCTN2 wild-type sequence (upper lane) with a heterozygous silent point mutation (c.277C>T) in the isogenic control. The middle lane shows introduction of the homozygous OCTN2 (N32S) c.95A>G point mutation in exon 1. Due to the large deletion of OCTN2 (−/−), Sanger sequencing could not be aligned (lower lane). PCR products with internal and flanking primers are shown in Figures S1D–S1F. The knockout was confirmed by quantitative reverse-transcription PCR (qPCR) of the *SLC22A5* transcript (Figure S1G). Southern blots validated the integrity of the edited locus. Figure S2A displays the predicted cutting sites for restriction endonucleases HindIII and EcoRI. Predicted fragment size and Southern blot results are shown in Figures S2B and S2C. NanoString analysis revealed normal karyotypes (Figure S2D).

### Functional analysis

Cardiomyocytes were differentiated from all three hiPSC lines. Figure S3A shows no difference in the percentage of cardiac troponin T (cTnT)-positive cells (OCTN2 (+/+), 87.5% ± 2.5%, n = 10; OCTN2 (N32S), 84.1% ± 5.0%, n = 10; OCTN2 (−/−), 86.2% ± 8.2%, n = 9).

**Figure 1:**
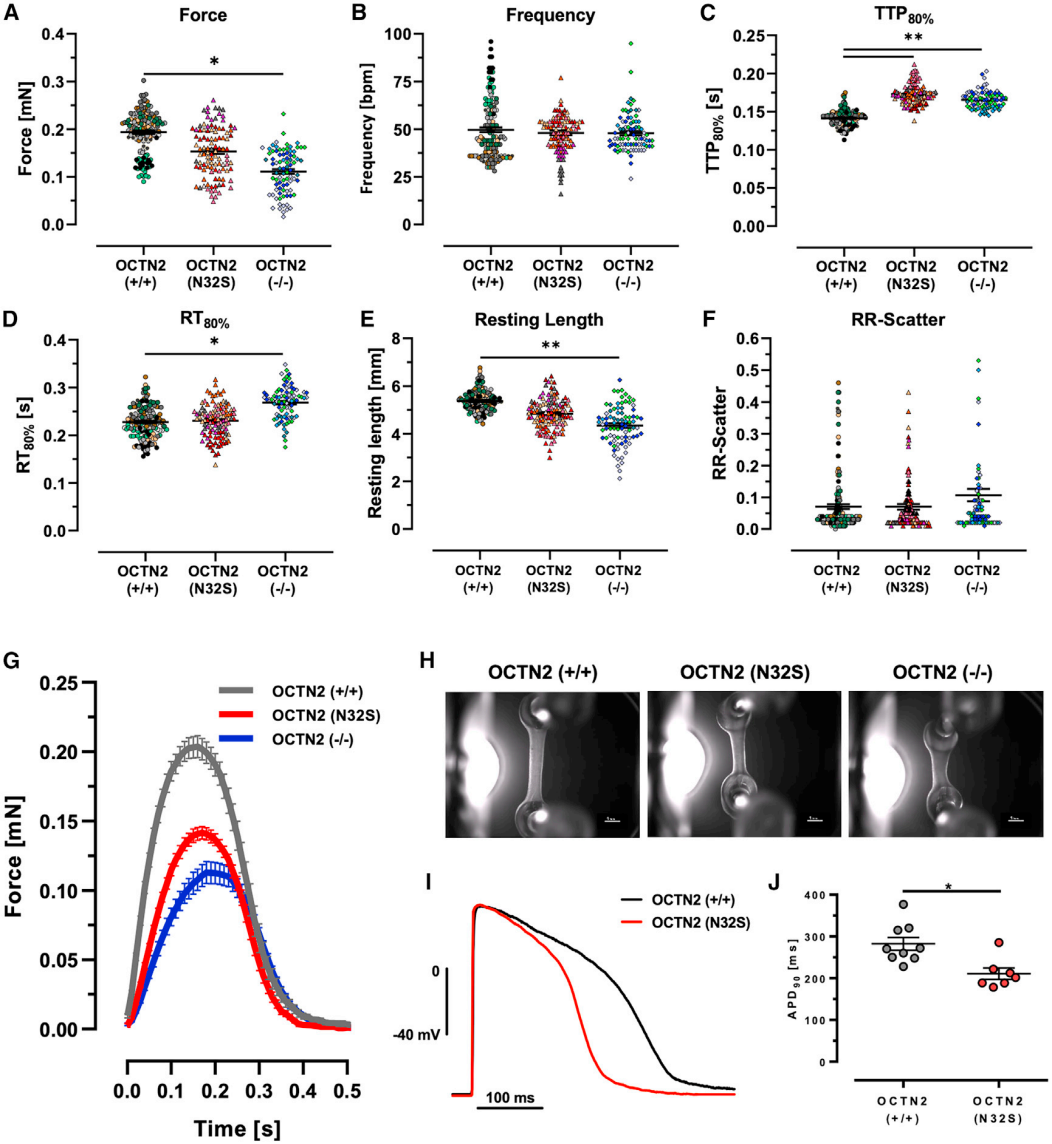
Contractile phenotype of OCTN2 genotypes (A–H) Effect of OCTN2 genotype on contractile parameters of spontaneous beating EHTs on day 21. OCNT2 (+/+), n = 153 EHTs from 9 batches; OCTN2 (N32S), n = 108 EHTs from 7 batches; OCTN2 (−/−), n = 91 EHTs from 5 batches. Nested one-way ANOVA followed by Bonferroni’s post test for multiple comparisons, ^∗^p < 0.05, ^∗∗^p < 0.01, ^∗∗∗^p < 0.001, ^∗∗∗∗^p < 0.0001. Each data point represents one EHT. Each color represents one independent differentiation batch. Data are expressed as mean ± SEM. (G) Representative average EHT contraction peaks of OCTN2 (+/+), OCTN2 (N32S), and OCTN2 (−/−). EHTs were electrically paced at 1.5 Hz in standard EHT medium, n = 9–14 EHTs from one batch. (H) Representative video-optical EHT images. Scale bars, 1 mm. (I and J) (I) Action potential measurement: representative action potential for OCTN2 (+/+) and OCTN2 (N32S). (J) Action potential duration (APD90) of OCTN2 (+/+) and OCTN2 (N32S) by sharp microelectrode measurement at 1.5 Hz. Student’s t test vs. OCTN2 (+/+), ^∗^p < 0.05. Data are expressed as mean ± SEM. Each data point represents one EHT. See also Figure S3.

**Figure 2:**
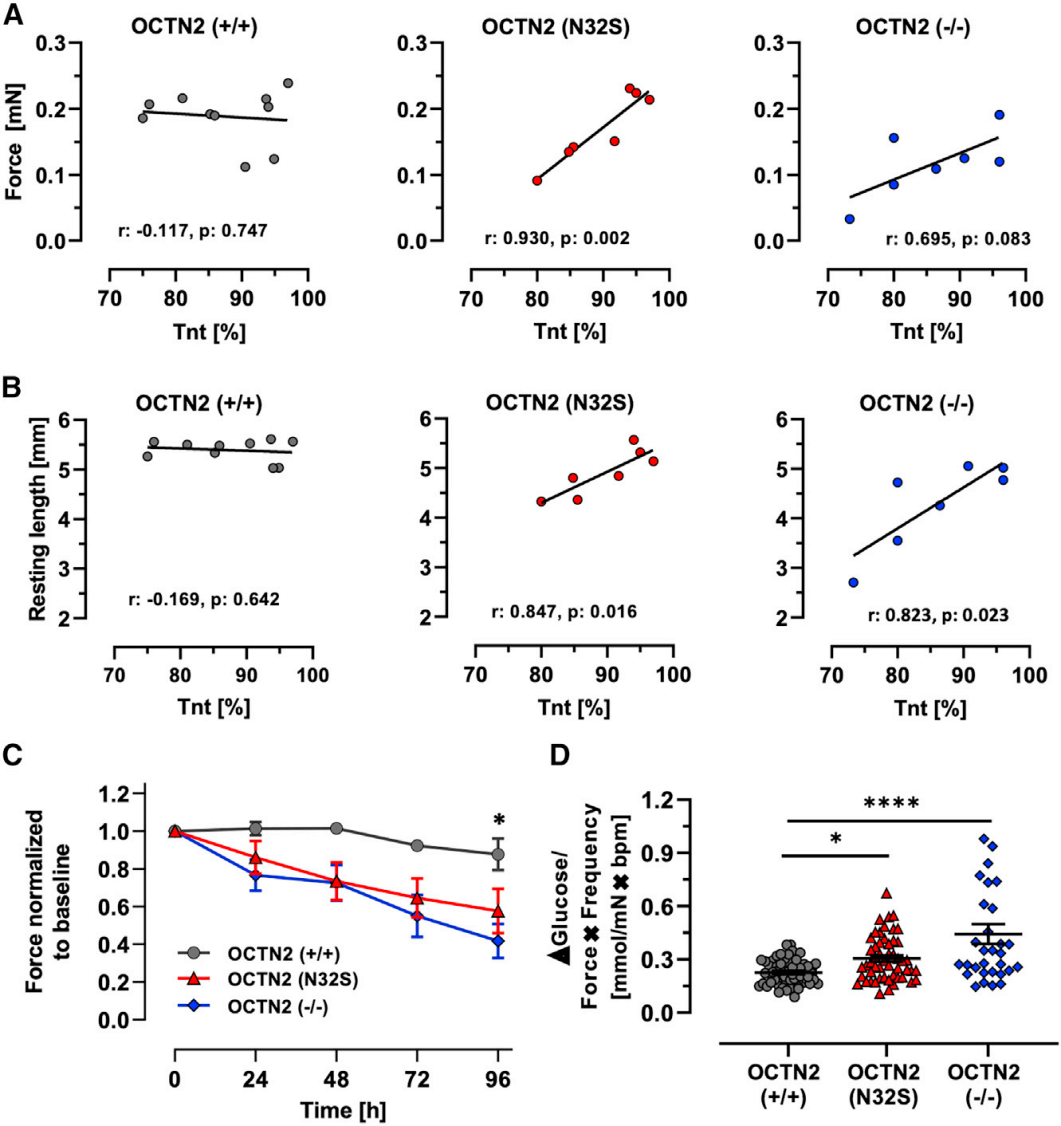
Effect of input cell compostion and cell culture media on EHT properties (A and B) Pearson correlation of (A) force and (B) resting length of EHTs with percentage of cTNT-positive input cells for EHT generation. OCTN2 (+/+), n = 10; OCTN2 (N32S), n = 7; OCTN2 (−/−), n = 7 differentiation batches. Each replicate represents the mean value of 7–20 EHTs for the specific differentiation batch. (C) EHT force development in fatty acid medium. Serum-free cell culture medium was supplemented with 50 μM carnitine, linoleic acid- and oleic acid-albumin. Data are normalized to baseline force. OCNT2 (+/+), n = 11 EHTs from 2 batches; OCTN2 (N32S), n = 11 EHTs from 2 batches; OCTN2 (−/−), n = 12 EHTs from 2 batches. Two-way ANOVA vs. OCNT2 (+/+) followed by Bonferroni’s post test for multiple comparisons, ^∗^p < 0.05. Data are expressed as mean ± SEM. (D) Difference in ΔGlucose medium concentration divided by product of individual spontaneous beating frequency × force. ΔGlucose = glucose concentration at baseline minus glucose concentration after 24 h of incubation in medium containing 5.5 mM glucose and 10% horse serum. OCNT2 (+/+), n = 59 EHTs from 5 batches; OCTN2 (N32S), n = 51 EHTs from 4 batches; OCTN2 (−/−), n = 28 EHTs from 4 batches. One-way ANOVA followed by Bonferroni’s post test for multiple comparisons, ^∗^p < 0.05, ^∗∗^p < 0.01, ^∗∗∗^p < 0.001, ^∗∗∗∗^p < 0.0001. One data point represents one EHT. Data are expressed as mean ± SEM. See also Figure S3.

Spontaneously beating engineered heart tissues (EHTs) were subjected to video-optical force analysis starting on day 7 (Figures S3B–S3G). Contractile parameters reached a plateau after day 21. OCTN2 (−/−) showed lower force, higher contraction time, and shorter resting length (length of the EHT at relaxed state) for the entire culture time. Contractile values of day 21 were compared (Figures 1A–1F) and showed lower force and a shorter resting length for OCTN2 (−/−) compared with OCTN2 (+/+) (OCTN2 (+/+), 0.194 ± 0.004 mN, n = 153 EHTs; OCTN2 (N32S), 0.16 ± 0.01 mN, n = 108 EHTs; OCTN2 (−/−), 0.11 ± 0.01 mN, n = 91 EHTs). Both OCTN2-defective genotypes exhibited longer contraction time, and additionally OCTN2 (−/−) a longer relaxation time. No difference for the RR scatter, a surrogate for arrhythmic beating, could be detected. The effect of proarrhythmic triggers were not studied. Figures 1G and 1H and Videos S1, S2, and S3 depict representative average contraction peaks, images, and videos. Sharp microelectrode experiments revealed shorter APD_90_ of 211.0 ± 13.6 (SEM, n = 7) in OCTN2 (N32S) vs. 288.2 ± 15.5 (SEM, n = 9) in OCTN2 (+/+) (Figures 1I and 1J). Importantly, OCTN2 (+/+) showed no correlation between cardiomyocyte purity (% cTNT^+^ input cells) and force as recently described (Mannhardt et al., 2020) and resting length in contrast to the OCTN2-defective genotypes (Figures 2A and 2B). This could suggest a relevant impact of non-cardiomyocytes on the phenotype.

Video S1. Spontaneously beating OCTN2 (+/+) EHT

Video S2. Spontaneously beating OCTN2 (N32S) EHT

Video S3. Spontaneously beating OCTN2 (−/−) EHT

In medium containing long-chain fatty acids (LCFA) plus carnitine [50 μM], force remained stable in OCTN2 (+/+) EHTs but declined in OCTN2-defective EHTs (Figure 2C), indicating a reduced ability to metabolize LCFA. In glucose and serum-containing EHT medium, delta-glucose and lactate values were higher for OCTN2 (N32S), but not OCTN2 (−/−) compared with OCTN2 (+/+). The delta-lactate/delta-glucose ratio as a surrogate for anaerobic glucose metabolism showed no difference (Figures S3H–S3J). Higher glucose consumption for OCTN2 (N32S) and OCTN2 (−/−) became evident when normalized to workload (force × beating frequency) (OCTN2 (+/+), 0.23 ± 0.01 mM/bpm × mN; OCTN2 (N32S), 0.3 ± 0.1 mM; OCTN2 (−/−), 0.4 ± 0.1 mM) (Figure 2D).

### Proteomics, Seahorse

**Figure 3:**
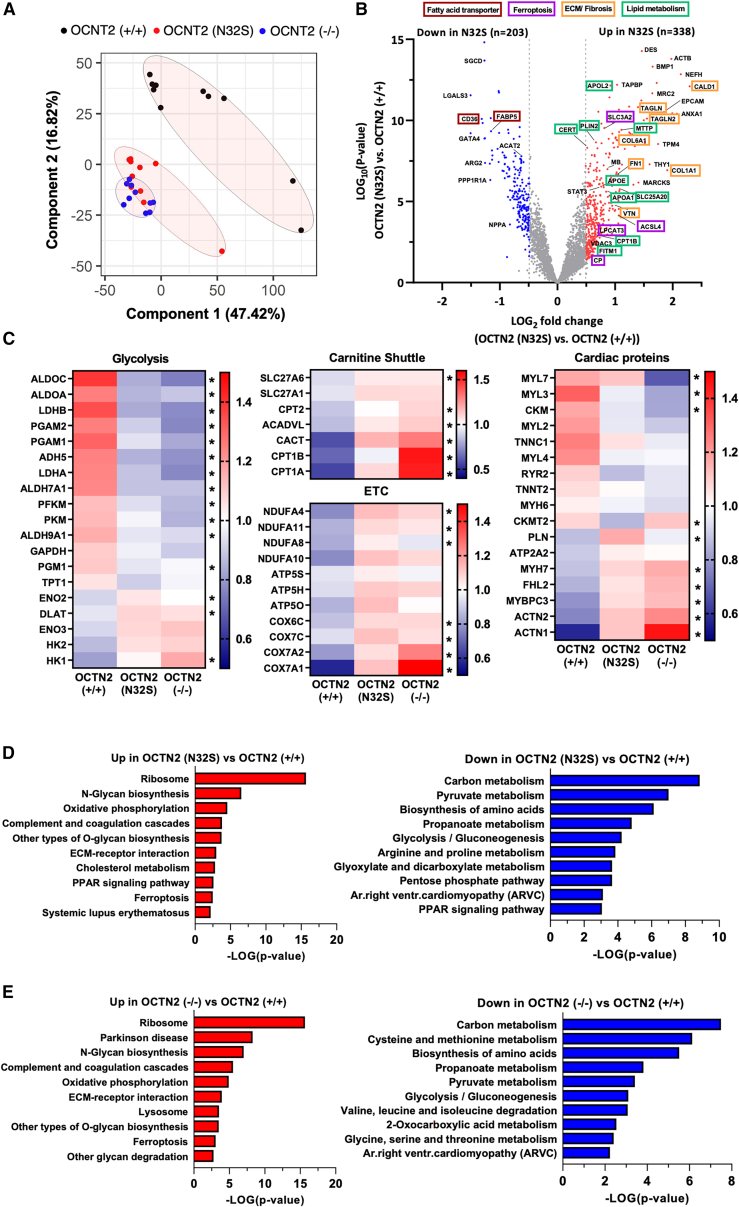
Tandem mass tag-based quantitative proteomic analysis of EHTs (A) Principal-component analysis (PCA) of OCNT2 (+/+) (black, n = 10) OCTN2 (N32S) (red, n = 10), and OCTN2 (−/−) (blue, n = 10) EHTs based on their proteomic profiles. Each dot represents one EHT. (B) Volcano plot of log2 fold changes of OCTN2 (N32S) vs. OCTN2 (+/+) and log10 of the p values with color-coded significance levels (p > 0.05) and fold change >1.4. (C) Clustering analysis of proteins related to metabolic pathways and the myocardium. Heatmaps display the relative abundance of proteins involved in glycolysis, carnitine shuttle, electron transport chain (ETC), and the myocardium. OCNT2 (+/+), mean of 10 EHTs from 1 batch; OCTN2 (N32S), mean of 10 EHTs from 1 batch; OCTN2 (−/−), mean of 10 EHTs from 1 batch. Protein levels are depicted as a color code ranging from blue (low abundance) to red (high abundance). Kruskal-Wallis test, ^∗^ indicates statistically significant difference of OCTN2 (+/+) against OCTN2 (N32S) or OCTN2 (−/−). (D and E) Pathway enrichment analysis of proteins identified by proteomic analysis. Depicted are KEGG pathways of significantly enriched proteins that were significantly higher (red) or lower (blue) abundant in (D) OCTN2 (N32S) vs. OCTN2 (+/+) and (E) OCTN2 (−/−) vs. OCTN2 (+/+), p < 0.05, fold change >1.4. See also Figure S4.

A total of 3,425 proteins was detected by tandem mass tag-based proteomic analysis, of which 1,772 proteins differed significantly between OCTN2 (+/+) and OCTN2 (N32S) and 2,050 differed significantly between OCTN2 (+/+) and OCTN2 (−/−), respectively (p < 0.05). A detailed summary of detected proteins is shown in Table S1, sheet 1. Due to their low abundance, OCTN2 or low-affinity transporters (SLC22A16, SLC16A9, and SLC6A14) were not detected. Principal-component analysis revealed separate clustering of OCTN2 (+/+) from OCTN2 (N32S) and OCTN2 (−/−) (Figure 3A). Volcano plot depiction highlights higher abundance of fibrosis/extracellular matrix proteins such as caldesmon1, collagen type I alpha 1 chain, transgelin 2, fibronectin 1, and vitronectin in OCTN2 (N32S) EHTs (Figure 3B). Moreover, ceramide transfer protein (CERT) was among the 10 most abundant proteins in OCTN2 (N32S). In contrast, the fatty acid transporters cluster of differentiation 36, fatty acid-binding protein 5, and cardiomyogenesis transcriptional regulator GATA binding protein 4 were among the top 10 lower abundant proteins in OCTN2 (N32S). Detailed grouping of proteins related to participation in pathways revealed concordant expression patterns for OCTN2 (N32S) and OCTN2 (−/−) vs. OCTN2 (+/+) (Figures 3C and S4C). KEGG pathway analysis revealed enrichment of proteins related to ribosome, ferroptosis, extracellular matrix, and N-glycan and O-glycan biosynthesis in OCTN2 (N32S) and OCTN2 (−/−). Conversely, enrichment analysis of lower abundant proteins highlighted the KEGG pyruvate- and propanoate metabolism, glycolysis, and pentose phosphate pathways. Furthermore, anti-ferroptotic cysteine metabolism was underrepresented in the OCTN2 (−/−) (Figures 3D and 3E). A detailed summary of proteins in the enriched pathways is depicted in Table S1, sheets 2 and 3. Mitochondrial DNA was lower in OCTN2 (N32S) (Figure S4A). Both OCTN2-defective lines showed lower oxygen consumption rate at baseline, as well as in response to oligomycin, carbonyl cyanide-p-trifluoromethoxyphenylhydrazone, and rotenone exposure in Seahorse experiments in glucose-based medium, suggesting lower glucose oxidation (Figure S4B).

### Carnitine supplementation: Acylcarnitine and ceramide content, force, lipid mass spectrometry, TEM

**Figure 4:**
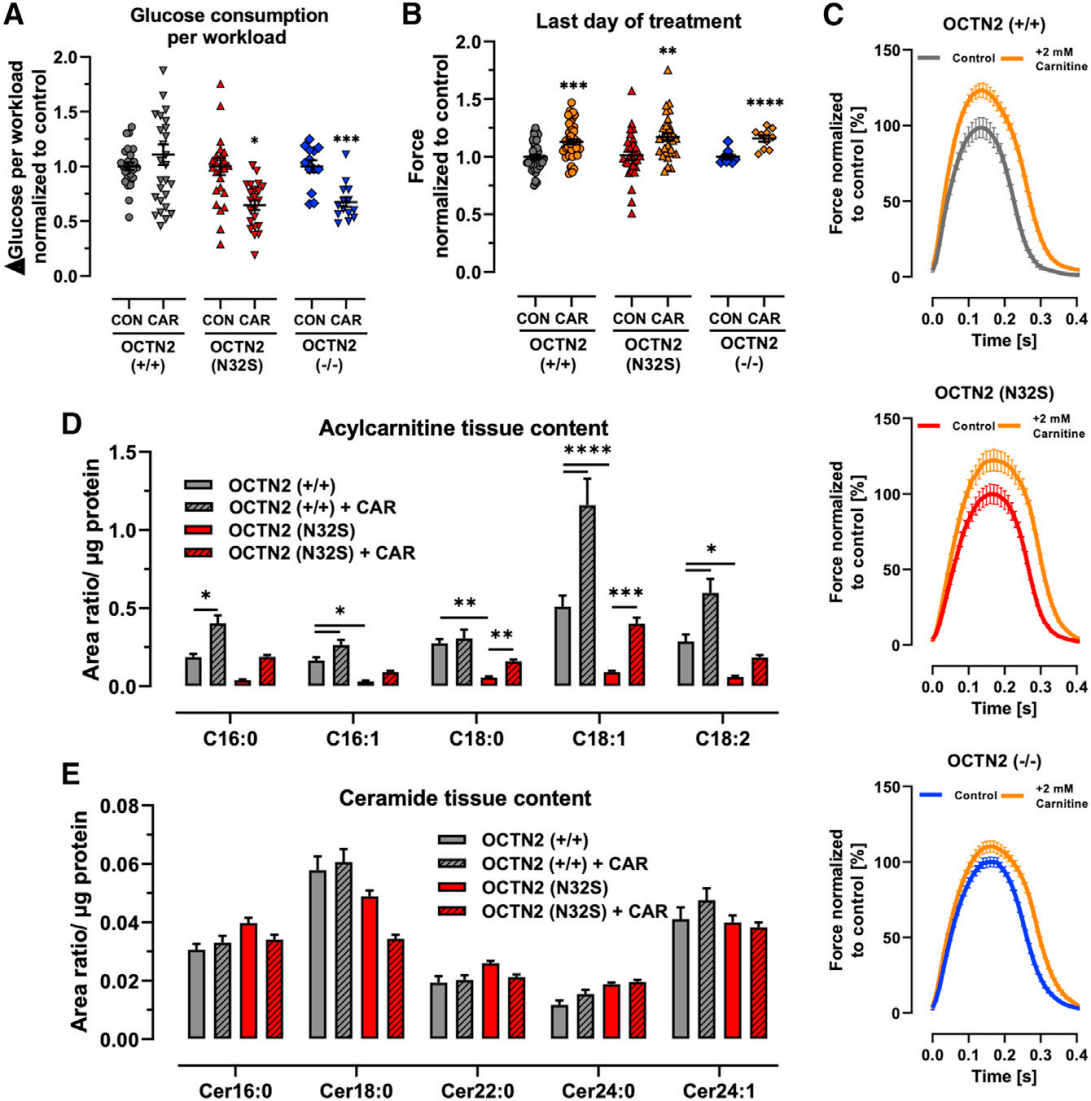
Effect of carnitine medium supplementation on EHT properties (A) Effect of carnitine supplementation ΔGlucose per workload (ΔGlucose = Glucose concentration at baseline minus glucose concentration after 24 h of incubation; workload = force × frequency). Nested t test vs. CON, ^∗∗^p < 0.01, ^∗∗∗^p < 0.001, ^∗∗∗∗^p < 0.0001. OCNT2 (+/+) control, n = 27 EHTs from 3 batches; OCNT2 (+/+) + carnitine (2 mM), n = 28 EHTs from 3 batches; OCTN2 (N32S) control, n = 23 EHTs from 3 batches; OCTN2 (N32S) + carnitine (2 mM), n = 23 EHTs from 3 batches; OCTN2 (−/−) control, n = 13 EHTs from 3 batches; OCTN2 (−/−) + carnitine (2 mM), n = 16 EHTs from 3 batches. Data are expressed as mean ± SEM. (B) Effect of carnitine supplementation on force of spontaneous beating EHTs at the last day of treatment (days 33-42). Values were normalized to last day of treatment of untreated control. Student’s t test vs. CON, ^∗∗^p < 0.01, ^∗∗∗^p < 0.001, ^∗∗∗∗^p < 0.001. OCNT2 (+/+) control, n = 54 EHTs from 4 batches; OCNT2 (+/+) + carnitine (2 mM), n = 49 EHTs from 4 batches; OCTN2 (N32S) control, n = 36 EHTs from 3 batches; OCTN2 (N32S) + carnitine (2 mM), n = 33 EHTs from 3 batches; OCTN2 (−/−) control, n = 9 EHTs from 1 batch; OCTN2 (−/−) + carnitine (2 mM), n = 9 EHTs from 1 batch. Data are expressed as mean ± SEM. (C) Effect of carnitine supplementation on average contraction peaks. Depicted are representative average EHT contraction peaks of OCTN2 (+/+), OCTN2 (N32S), and OCTN2 (−/−). EHTs were electrically paced at 1.5 Hz in standard EHT medium ± carnitine (2 mM). Values were normalized to untreated control. n = 9–16 EHTs per condition from 1 batch. (D and E) Liquid chromatography-mass spectrometry analysis of acylcarnitines and ceramides. Effect of carnitine supplementation on (D) acylcarnitine and (E) ceramide content of OCNT2 (+/+) and OCTN2 (N32S) EHTs after 33 days of culture and supplementation. Two-way ANOVA followed by Bonferroni’s post test for multiple comparisons, ^∗^p < 0.05, ^∗∗^p < 0.01, ^∗∗∗^p < 0.001, ^∗∗∗∗^p < 0.0001. Data are expressed as mean ± SEM. n = 4 EHT pools (containing 3 EHTs each) per genotype and carnitine supplementation from 1 batch. See also Figure S5.

Supraphysiological carnitine concentration (2 mM, replicating plasma concentrations during treatment) led to a reduction of glucose consumption and lactate production for all genotypes (Figures S5A–S5C). Notably, glucose consumption normalized to cardiac workload was reduced only for the OCTN2-defective cell lines (Figure 4A). This was associated with an increase in force and relaxation time for all lines (Figures 4B, 4C, and S5D–S5G). Transcript levels of PDK4, the inhibitor of PDH and important metabolic regulator, were higher in OCTN2 (N32S) and OCNT2 (−/−) and attenuated to the level of isogenic control by carnitine supplementation (Figures S6H and S6I). Liquid chromatography-mass spectrometry revealed 5-fold lower content for C16:1, C18:0, C18:1, and C18:2 acylcarnitines in OCTN2 (N32S). Carnitine supplementation resulted in a higher content of C16:0, C16:1, C18:1, and C18:2 acylcarnitines for OCTN2 (+/+) and C18:1 and C18:2 acylcarnitines for OCTN2 (N32S). Quantification of ceramide content, a metabolite of accumulated acyl-CoA (Cer16:0, Cer18:0, Cer22:0, Cer24:0, Cer24:1) revealed neither an effect of the genotype nor carnitine supplementation (Figures 4D and 4E).

**Figure 5:**
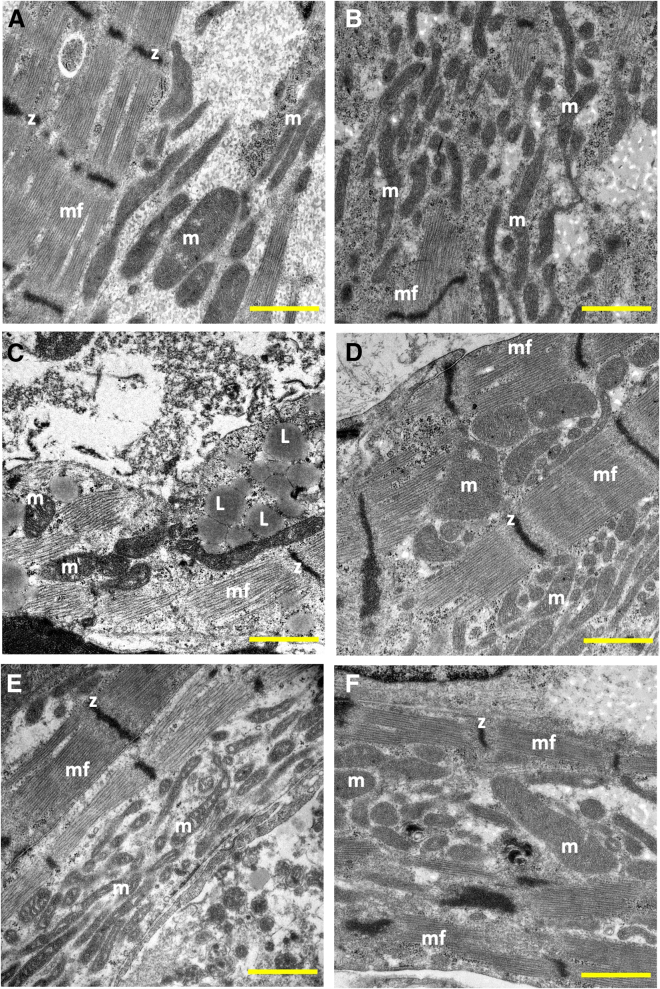
Transmission electron microscopy of OCTN2 EHTs (A and B) OCTN2 (+/+), (C and D) OCTN2 (N32S), and (E and F) OCTN2 (−/−). (A, C, and E) Untreated. (B, D, and F) Supplemented with carnitine (2 mM). mf, myofilaments; z, z-line; m, mitochondria; L, lipid droplet. Scale bars, 1 μm. See also Figure S4.

Transmission electron microscopy (TEM) (Figures 5A–5F) showed elongated myofilaments and structured mitochondria in OCTN2 (+/+) EHTs. OCTN2 (N32S) displayed lower abundance of mitochondria and moreover with structural defects, and high frequency of large lipid droplets in close association with mitochondria and sarcomeres. OCTN2 (−/−) also exhibited mitochondria with disturbed structures and increased membrane density but less aggregation of lipid droplets. Carnitine supplementation increased mitochondria for all genotypes and reduced the occurrence of lipid droplets. Further high-power TEM analysis revealed electron dense matrix (Dixon et al., 2012) and loss of cristae (Yagoda et al., 2007) and rupture of the outer mitochondrial membrane (Friedmann Angeli et al., 2014) but preserved nuclear membrane morphology (N) (Figure S4D–S4O) in the OCNT2-defective lines indicating hallmarks of ferroptosis.

### Single nuclear RNA sequencing

**Figure 6:**
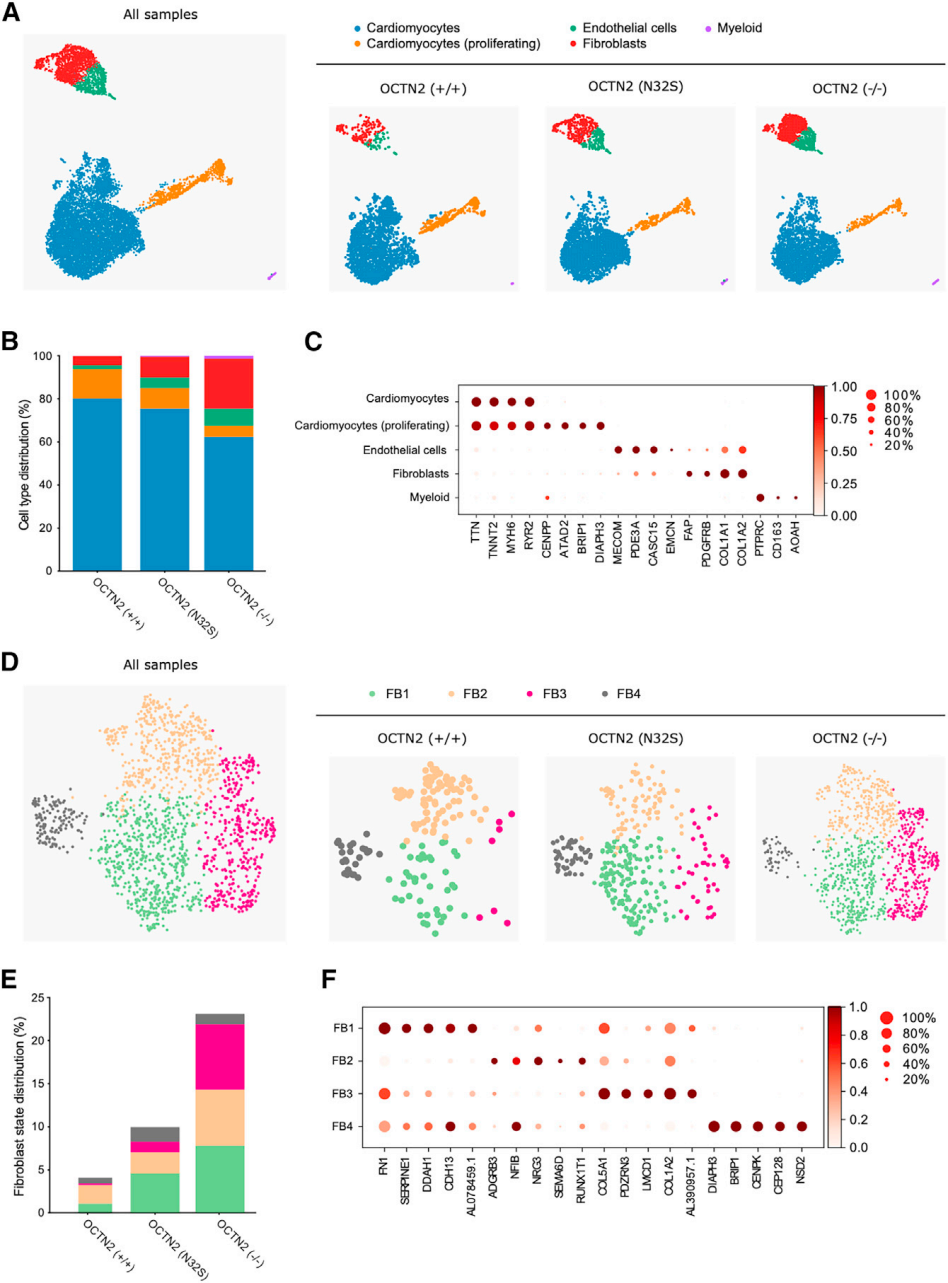
Cellular heterogeneity in OCTN2 genotypes in EHTs (A) Representative UMAP plot after snRNA-seq of all samples and individual genotypes, n = 1 EHT pool (4 EHTs) per genotype; OCTN2 (+/+) (3,674 cells), OCTN2 (N32S) (4,525 cells), OCTN2 (−/−) (5,108 cells). Five distinct cell clusters were identified: cardiomyocytes, cardiomyocytes (proliferating), endothelial cells, fibroblasts, and myeloid cells. (B) Percentage of cell types per genotype. (C) Dot plot graph showing the relative expression of cell-specific marker genes. Expression levels are depicted as a color code ranging from light red (low expression) to dark red (high expression) as mean of log2 fold of expression. The dot size indicates the percentage of cells expressing the gene. (D) Representative fibroblast subcluster FB1-4 UMAP plot of all samples and individual genotypes. (E) Percentage of fibroblast states per genotype. For each genotype, the total percentage of fibroblast states equals the percentage of fibroblast abundance identified in (B). (F) Dot plot graph showing the relative expression of fibroblast-specific marker genes in fibroblast states. Scaled expression levels are depicted as a color code ranging from light red (low expression) to dark red (high expression) as mean of log2 fold of expression. The dot size indicates the percentage of cells expressing the gene. See also Figures S6 and S7.

A pool of four EHTs per genotype was subjected to single nuclear RNA sequencing (snRNA-seq). OCTN2 (+/+), (N32S), and (−/−) samples were sequenced with average sequencing depth of 39,324, 28,771, and 26,374 reads per nucleus. Following quality control filtering, snRNA-seq data were pooled to a total number of 11,225 nuclei (OCTN2 (+/+) = 3,135, OCTN2 (N32S) = 3,761, OCTN2 (−/−) = 4,329 cells). Uniform Manifold Approximation and Projection (UMAP) and Leiden clustering revealed 5 main cell clusters. Marker genes distinguished: cardiomyocytes, proliferating cardiomyocytes, fibroblasts, and endothelial and myeloid cells (Figure 6A). In OCTN2 (+/+), cardiomyocytes represented 94% of all cells with 14% of these cells showing markers of proliferation (Figures 6B and 6C). Subclustering of cardiomyocytes revealed 10 subclusters (Figure S6). Subcluster CM4 was dominant in OCTN2 (+/+), while CM1 and 2 were more prominent in OCTN2-defective lines. Interestingly, KEGG analysis revealed enrichment of the GPR40 pathway in CM4, describing free fatty acid receptor 1 signaling (Figure S6C). A lower representation of CM4 in the OCTN2-defective lines is compatible with the lower abundance of fatty acid transporters in the proteomics analysis. In OCTN2-defective lines, cardiomyocytes represented a smaller fraction (OCTN2 (N32S), 85%; OCTN2 (−/−), 67%). Reversely, these lines showed a higher fraction of fibroblasts (OCTN2 (+/+), 4%; OCTN2 (N32S), 10%; OCTN2 (−/−), 23%) (Figures 6B and 6C). Subclustering of fibroblasts identified four states. Fibroblasts states with markers of TGF-β signaling, proliferation, and secretion (FB1, FB3, and FB4), were more prominent in OCTN2-defective lines (Figures 6D, 6E, 6F, and S7A). Genotype-specific analysis of significant KEGG pathway enrichment in all fibroblast subcluster revealed relaxin-, ECM-, and focal adhesion-related pathways (Figure S7B).

Endothelial and myeloid cells were almost absent in OCTN2 (+/+) and represented 5% and 1% in OCTN2 (N32S) and 8% and 1% in OCTN2 (−/−), respectively (Figure 6B). Endothelial cells expressed PDE3A, CASC15, and MECOM, and myeloid cells expressed PTPRC (CD45), CD163, and AOAH (Figure 6C). KEGG pathway analysis was not possible because significant pathways could only be detected for OCTN2 (−/−) endothelial cells (Figure S7B).

### Ferroptosis, fibroblast activation

**Figure 7:**
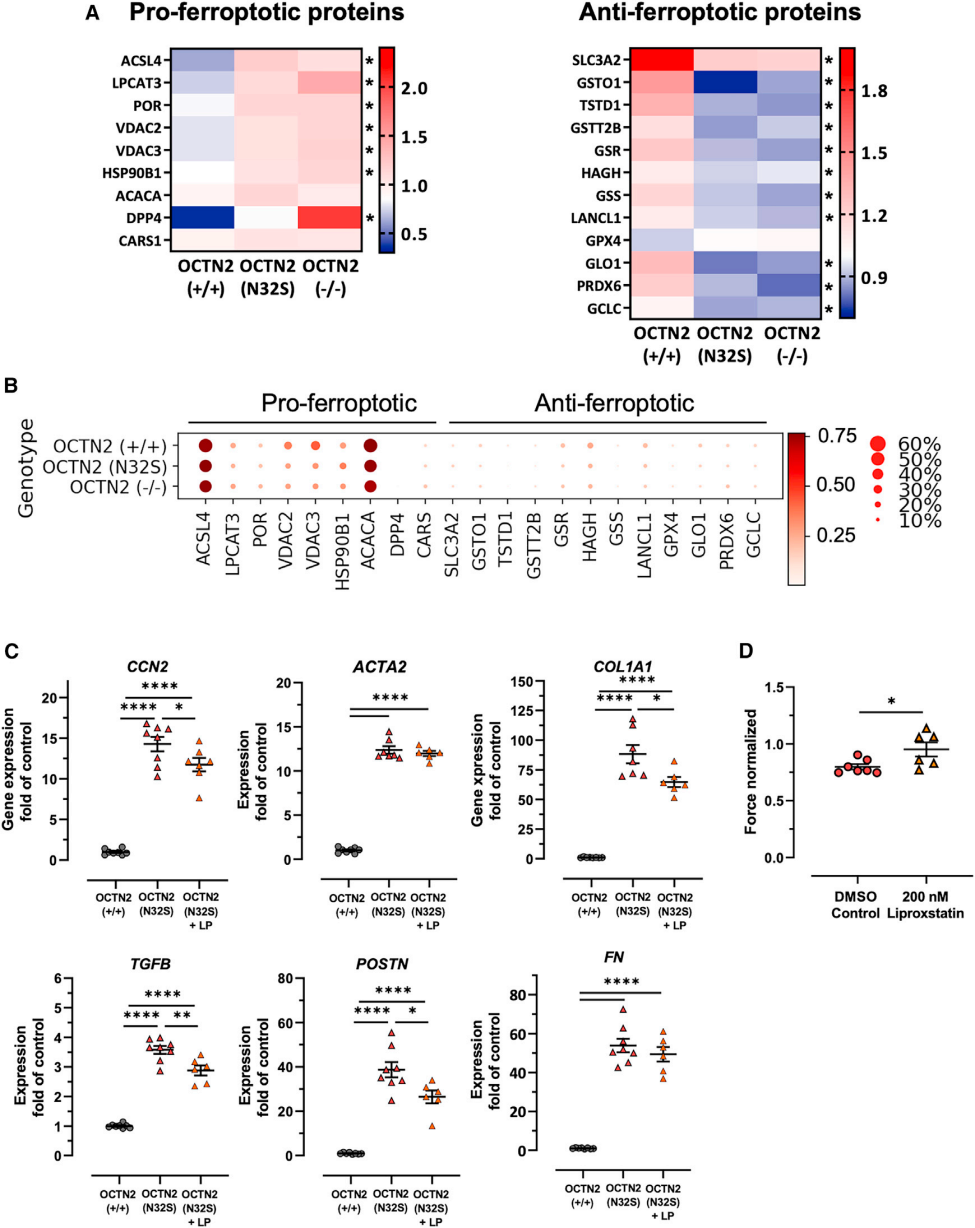
Evidence for ferroptosis pathway activation in tandem mass tag-based quantitative proteomic analysis and pharmacological inhibitor experiments (A) Proteomic analysis heatmaps display the relative abundance of pro- and anti-ferroptotic proteins of all genotypes. OCNT2 (+/+), mean of 10 EHTs from 1 batch; OCTN2 (N32S), mean of 10 EHTs from 1 batch; OCTN2 (−/−), mean of 10 EHTs from 1 batch. Protein levels are depicted as a color code ranging from blue (low abundance) to red (high abundance). (B) snRNA-seq dot plot graph showing the scaled relative expression of pro- and anti-ferroptotic markers across all genotypes for all cells. The dot size indicates the percentage of cells expressing the respective gene. (C) Effect of the ferroptosis inhibitor liproxstatin: qPCR analysis gene expression of genes related to fibroblast activation. Gene expression was normalized to GUSB over OCTN2 (+/+) control. OCNT2 (+/+), n = 8 EHTs from 1 batch; OCTN2 (N32S), n = 8 EHTs from 2 batches; OCTN2 (−/−), n = 6–8 EHTs from 1 batch. One-way ANOVA followed by Bonferroni’s post test for multiple comparisons, ^∗^p < 0.05, ^∗∗^p < 0.01, ^∗∗∗∗^p < 0.0001. Data are expressed as mean ± SEM. (D) Effect of liproxstatin (200 nM) on contractile force in OCTN2 (N32S) EHTs. Data are expressed as mean ± SEM, ^∗^p < 0.05, unpaired t test. See also Figure S7.

Proteomics analysis revealed enrichment of the KEGG pathway ferroptosis, an iron-dependent lipid peroxidation-mediated cell death mechanism. Extraction of an extended list of pro- and anti-ferroptotic proteins (Chen et al., 2021) from the proteomics data identified a surprisingly uniform regulation with higher abundance of pro- and lower abundance of anti-ferroptotic proteins in the OCTN2-defective genotypes (Figure 7A). Noteworthy among these were also the key regulators ACSL4 (Doll et al., 2017) and LPCAT3, which synergistically drive the accumulation of iron-dependent toxic lipid peroxides (Li and Li, 2020). On the other hand, pro- and anti-ferroptosis transcripts did not show a differential expression in snRNA-seq, suggesting post-transcriptional regulation (Figure 7B). Evidence for both ferroptosis and fibroblast activation in this study and previous reports that demonstrate a mechanistic link (Fang et al., 2019; Gong et al., 2019) provided the rationale to analyze the effect of the potent ferroptosis inhibitor liproxstatin on fibrosis markers. OCTN2 (N32S) EHTs revealed higher transcript levels of *ACTA2*, *COL1A1*, *POSTN*, *TGFB*, *FN*, and *CCN2* than OCTN2 (+/+). Liproxstatin induced a significant attenuation of fibrosis transcript levels in OCTN2 (N32S), which was associated with a moderate increase in force (Figures 7C and 7D).

## Discussion

This study describes the first hiPSC PCD DCM *in vitro* model. The main results are (1) successful genetic engineering of two hiPSC lines, a homozygous OCTN2 (N32S) mimicking the patient situation and an OCTN2 (–/–) knockout, (2) replication of the PCD DCM phenotype involving low acylcarnitine tissue content and force, complex metabolic remodeling, and ultrastructural alteration, (3) corroboration of the of OCTN2 (N32S) phenotype by high-level concordance with OCTN2 (−/−) across various assays, (4) discovery of ferroptosis linked to fibroblast activation as a novel PCD DCM mechanism.

The approach to engineer the human-relevant OCTN2 (N32S) loss of function point mutation in parallel with a complete OCTN2 knockout turned out to be insightful since concordant changes of several parameters (e.g., contractile parameters, proteomics profile, snRNA-seq clustering of (non)-cardiomyocyte subpopulations) were observed in both lines, thus validating lack of functional OCTN2 as the main pathomechanism governing disease origin.

Typical features of PCD DCM in both patients and animal models are reduced tissue contents of carnitine derivates, functional impairment of glucose and lipid metabolism, myocardial steatosis, severe hyperglycemia (Horiuchi et al., 1993; Asai et al., 2006), and short QT syndrome (Roussel et al., 2016). Several key features could be successfully replicated in this hiPSC-CM model such as lower force and higher sensitivity to fatty acid-based media, reduced acylcarnitine tissue content intracellular lipid droplet accumulation, and shorter action potential duration in OCTN2-defective EHT. Genome-wide analysis revealed complex metabolic remodeling and mitochondrial dysfunction compatible with (acyl)-carnitine deprivation (Asai et al., 2006). Noteworthy, dysregulation of several metabolic pathways (e.g., pyruvate metabolism, glycolysis, and the pentose phosphate pathway) were not as expected in a carnitine-deficient and fatty acid oxidation-impaired model. Likewise, the higher abundance of proteins related to carnitine metabolism (e.g., CPT1, CPT2, and CACT (SLC25A20)) was unexpected. This apparent discrepancy is related to the integration of primary changes and also compensatory metabolic adaptation in the proteomic analysis. Hence, the upregulation of carnitine shuttle proteins likely represents a compensatory effect as also described in secondary carnitine deficiency animal models (Liepinsh et al., 2008). The lower and higher abundance of proteins related to glucose metabolism and oxidative phosphorylation indicates dysregulation of pathways related to glucose and lipid metabolism. In the case of glucose, the Seahorse experiments and high PDK4 expression levels indicate a deficit in glucose oxidation, which is line with the established carnitine-mediated activation of glucose oxidation (Broderick et al., 1995).

Low cytoplasmic carnitine concentration leads to diminished acylcarnitine formation and subsequent beta-oxidation (Longo et al., 2006). Indeed, mass spectrometry analysis revealed lower tissue content of several long-chain acylcarnitines in OCTN2 (N32S) EHTs. In addition, declining force in LCFA media suggested a defect in LCFA metabolism. Surprisingly, mass spectrometry did not reveal a difference in ceramide content. However, ceramides do not represent the final product, but rather a metabolic intermediate that can be processed to sphingolipid derivates such as glucosylceramides and sphingomyelin (MacEyka and Spiegel, 2014). For this conversion, ceramides are transported from the endoplasmic reticulum into the trans-Golgi apparatus by ceramide transporter CERT (Bandet and Hajduch, 2021). Remarkably, CERT was among the 10 most significantly higher abundant proteins in OCTN2 (N32S) EHTs, suggesting that it belongs to the compensatory mechanisms and prevents the accumulation of toxic ceramides.

PCD carnitine supplementation therapy results in plasma concentrations in the millimolar range, enabling intracellular transport via the low-affinity transporter ATB^0,+^ (SLC6A14) (Longo et al., 2016). Carnitine supplementation (2 mM) in this study had a strong effect on metabolic aspects such as acylcarnitine tissue content, glucose consumption per cardiac work, PDK4 transcript level, and lipid droplet accumulation. Nevertheless, the force restoration was minor and carnitine-mediated increase in force was similar for all three genotypes, suggesting a non-specific effect potentially related to the induction of sodium current and subsequent inhibition of Na⁺/K⁺-ATPase as described previously (Wu and Corr, 1994). The discrepancy between strong metabolic and small force effects of carnitine supplementation suggests the relevance of additional mechanisms. The shorter EHT resting length, the positive correlation between cardiomyocyte purity with force and resting length, and the enrichment of the KEGG pathway extracellular matrix in the OCTN2-defective genotypes point to the relevance of fibroblast activation. In support of this, snRNA-seq revealed a more prominent fibroblast cluster in the OCTN2-defective gen-otypes, which expressed markers indicative of activated and secretory fibroblast state. Of note, markers of activated fibroblasts in this study (*POSTN*, *FN1*, *FAP*, and *NOX4*) overlap substantially with the fibroblast signatures in two failing heart snRNA-seq DCM studies (Chaffin et al., 2022; Nicin et al., 2021). Interestingly, the central role of fibroblasts in this hiPSC-CM PCD model is paralleled by clinical findings of overt myocardial fibrosis in PCD patients (Wang et al., 2014; Tomlinson et al., 2018). This study does not allow conclusions to be drawn as to whether fibroblast activation is an autonomic or a paracrine effect via cardiomyocytes. In any case, fibroblast activation is not specific to ferroptosis in cardiomyopathies since it was also described in non-cardiac conditions (Zhang et al., 2021; Gong et al., 2019).

Ferroptosis is well compatible with PCD DCM, since it is driven by the accumulation of polyunsaturated fatty acids (PUFAs) linked to CoA in cell membranes (Yang et al., 2016). PUFA accumulation is relevant for PCD as cytosolic carnitine deficiency impairs PUFA metabolization to acylcarnitine. ACSL4 catalyzes the esterification of long-chain PUFA to acyl-CoA, represents a central pro-ferroptotic regulator (Doll et al., 2017), and showed higher expression and protein abundance in this model. The abundance of the central anti-ferroptotic enzyme GPX4 was not lower in OCTN2-defective lines. Notably, glutathione is an important cofactor for GPX4 activity and proteins involved in glutathione metabolism such as glutamate-cysteine ligase, glutathione synthetase, and glutathione-disulfide reductase were of lower abundance in OCTN2-defective lines.

Taken together, the development of a human PCD *in vitro* model and the discovery of ferroptosis linked to fibroblast activation reveals novel insight into PCD-associated metabolic cardiomyopathy and is paving the way for the development of more specific treatment strategies.

## Experimental procedures

A detailed description of experimental procedures is available online.

### Resource availability

#### Corresponding author

Arne Hansen, University Medical Center Hamburg-Eppendorf, Department of Experimental Pharmacology and Toxicology, 20246 Hamburg, Germany. E-mail: ar.hansen@uke.de

#### Materials availability

This study did not create any unique reagents. The genetically engineered OCTN2-deficient hiPSC lines are available with a completed material transfer agreement.

### hiPSC cell culture conditions

An established hiPSC control cell line (hiPSCreg code UKEi001-A) derived from a healthy individual served as the starting point for the genetic engineering approach and as the isogenic control for the engineered hiPSC lines. This hiPSC line was generated by reprogramming dermal fibroblasts from a skin biopsy using the CytoTune (Life Technologies) 2.0 Sendai Reprogramming Kit under feeder-free conditions. All basic stem cell culture work was performed as recently described (Shibamiya et al., 2020). In brief, hiPSC culture was based on the expansion of a master cell bank at passage 25–35 on Geltrex-coated cell culture flasks in FTDA medium (Table S2) under hypoxic conditions (5% O_2_). Standard passaging was performed twice a week (3–4 days passaging interval) with Accutase solution (Sigma-Aldrich) with a plating density of 4.5–7.0 × 10^4^ hiPSC/cm^2^. Maximal expansion was for 40 passages with regular screening for mycoplasma contamination by PCR amplification. SSEA3 surface marker served as a pluripotency marker and was analyzed by flow cytometry. All procedures involving the generation and analysis of hiPSC lines were approved by the local ethics committee in Hamburg (Az PV4798, 28.10.2014).

### CRISPR-Cas9-mediated gene editing

#### OCTN2 (N32S) missense mutation

The *SLC22A5* gene locus was Sanger sequenced in the hiPSC OCTN2 (+/+) control line. CRISPR-Cas9 technology was used to engineer the c.95A>G (N32S) mutation into the *SLC22A5* wild type. IDT Custom Alt-R CRISPR-Cas9 gRNA software and CRISPOR (Concordet and Haeussler, 2018) were used to identify potential gRNA binding sites at the gene locus. The OCTN2 NCBI Reference (NG_008982.2) was provided as a target sequence. Targets for gRNAs were chosen based on the lowest cut-to-mutation distance under consideration of a high on-target potential and low off-target risk. A single-stranded oligodeoxynucleotide served as an exogenous donor template, containing the OCTN2 c.95A>G, p.N32S mutation. In addition, a silent mutation was introduced in the PAM sequence to prevent CRISPR-Cas9 re-cutting after successful genomic integration of the template by HDR. Edited clones were identified by PCR amplification and subsequent Sanger sequencing. A schematic overview of the HDR strategy is depicted in Figure S1A. SsODN and gRNA sequences are shown in Table S3, sheet 1.

#### OCTN2 (−/−) knockout

A combinatorial CRISPR strategy was used to engineer a knockout of the *SLC22A5* gene in the isogenic control hiPSC OCTN2 (+/+). Two gRNAs were designed to introduce a deletion of 17.3 kb spanning from the promotor region to exon 5 (NG_008982.2). For deletion validation, primer pairs were designed to amplify products inside the deletion region and the gRNA target sites. Also, primers flanking the two cutting sites were designed. Edited clones were identified by PCR amplification and subsequent Sanger sequencing. A schematic overview of the knockout strategy is displayed in Figure S1B. To distinguish between unedited, heterozygous, and homozygous edited clones, the PCR products were separated by agarose gel electrophoresis (1%, w/v), followed by Midori green staining. The target gRNA sequences are shown in Table S3, sheet 1.

### Cardiac differentiation

hiPSCs were differentiated into cardiomyocytes with an embryoid body (EB)- and growth factor-based three-stage protocol which was recently described (Breckwoldt et al., 2017). In brief, hiPSCs were expanded on Geltrex-coated T80-flasks to a confluency of 90%–100% and detached with EDTA. The formation of EBs was induced in 500 mL spinner flasks with a density of 30–35 × 10^6^ hiPSCs per 100 mL of EB formation medium (Table S2). hiPSC suspension was cultivated overnight at 40 rpm glass ball impeller rotation speed. Mesoderm induction was induced in mesoderm induction medium (Table S2) with a volume of 200–300 μL EB per pluronic-coated T175-flask for 3 days under hypoxic conditions (5% O_2_) with 50% medium exchange daily. After washing the EBs again, cardiac differentiation was induced in cardiac differentiation medium 1 (Table S2) with a volume of 250–300 μL EB per pluronic-coated T175-flask with 50% medium exchange daily for 3 days under normoxic conditions (21% O_2_). Then, medium was completely removed and exchanged for cardiac differentiation medium 2 (Table S2). After a daily 50% medium change for 4 days, culturing medium was exchanged with cardiac differentiation medium 3 (Table S2). After washing EBs in HBBS solution buffer, beating cardiomyocytes were dissociated with collagenase II solution (200 units/mL; Worthington) containing myosin II ATPase inhibitor N-benzyl-p-toluene sulfonamide for 2–3 h until dispersing single cells could be observed. Dissociated hiPSC-CM were frozen in freezing medium containing 90% FBS and 10% DMSO or resuspended in EHT casting medium for subsequent EHT generation. Differentiation efficiency (% cTNT-positive cells) was determined by fluorescent-labeled cTNT antibody (Miltenyi Biotech) using a flow cytometer FACSCanto II (BD). Adjustment of gates adjusted according to the isotype control and performed with FACSDiva software (BD). Differentiation runs with at least 75% cTNT-positive cells were used for further functional experiments. FACS reagents are shown in Table S4.

### EHTs

EHTs were generated as recently described (Mannhardt et al., 2016). In brief, dissociated hiPSC-CM were centrifuged (100 × *g*, 10 min) and resuspended in EHT casting medium containing DMEM, horse serum, and glutamine (Table S2). Polytetrafluorethylene (PTFE) spacers (EHT Technologies) were placed in a warm 2% (w/v) agarose/PBS solution in a 24-well plate. Agarose solidification at room temperature led to the formation of agarose molds. PTFE spacers were removed from the 24-well plates and flexible polydimethylsiloxane (PDMS) posts were placed on the 24-well plates so that pairs of elastic PDMS posts reached into each casting mold. Mastermix (100 μL) (Table S2) containing 2× DMEM, Y-27632, fibrinogen and 1.0 × 10^6^ hiPSC-CMs was resuspended in EHT casting medium, rapidly mixed with 3 μL thrombin and pipetted into one agarose casting mold. Afterward the preparation was placed in an incubator for 1.5 h at 37°C until a fibrin gel formed in the agarose molds around the PDMS posts. Pre-warmed EHT culture medium (200–300 μL) was added dropwise into each well to ameliorate the detachment of the fibrin gel from the agarose mold. After an additional 15–30 min of incubation, racks with fibrin gels attached to the PDMS posts were transferred into a new 24-well plate, filled with pre-warmed EHT culture medium, and incubated at 40% O_2_, 37°C, 7% CO_2_, and 98% humidity. EHTs were cultured for 28–42 days with medium changes 3 times per week. After 5–7 days of culture, EHTs started to develop spontaneous macroscopic contractions.

### Video-optical contraction analysis

EHT contractile analysis was performed as described previously (Mannhardt et al., 2016; Breckwoldt et al., 2017). EHT contraction parameters, e.g., force, frequency, and contraction kinetics, were monitored over time of EHT development 2 h after each medium change. EHTs were electrically stimulated as described previously by Hirt et al. (2014). PDMS racks with EHTs were mounted onto custom-made graphite pacing units and stimulated by using a Grass S88X Dual Output Square stimulator (Natus Neurology Incorporated). The pacing frequency was adjusted to a value of 1.5- to 2-fold of the spontaneous beating frequency of the EHT batch with an output voltage of 2 V in biphasic pulses of 4 ms. EHTs that were not able to follow the pacing frequency were excluded from the analysis. Average contraction peaks were calculated with an average of 10–15 peaks.

## Data Availability

The mass spectrometry proteomics data have been deposited to the ProteomeXchange Consortium via the PRIDE [1] partner repository with the dataset identifier PXD036026. The snRNA-seq datasets are available in the Gene Expression Omnibus repository under accession no. GSE211650 (https://www.ncbi.nlm.nih.gov/geo/query/acc.cgi?acc=GSE211650). The acylcarnitine and ceramide mass spectrometry data are available under https://uni-koeln.sciebo.de/s/4ebUQhDuFH1UPoA.

## Supplemental information

Document S1. Figures S1–S7 and Tables S2 and S4

Table S1. Identified proteins and KEGG pathways by proteomic analysis Sheet 1. Detected proteins proteomic analysis. Sheet 2. KEGG enrichment analysis of proteins with significant higher abundance in OCTN2 (N32) and OCTN2 (––) vs. OCTN2 (++). Sheet 3. KEGG enrichment analysis of proteins with significant lower abundance in OCTN2 (N32) and OCTN2 (––) vs. OCTN2 (++).

Table S3. Nucleotide sequences for reprogramming and PCR Sheet 1. SsODN and gRNA sequences. Sheet 2. Primer pairs for PCR and Sanger sequencing. Sheet 3. Primer pairs for qPCR.

Document S2. Article plus supplemental information
