# Supplementary material for: Human model of primary carnitine deficiency cardiomyopathy reveals ferroptosis as a novel mechanism
Source: Stem Cell Reports. 2023 Oct 5;18(11):2123–37. doi: 10.1016/j.stemcr.2023.09.002 (PMC10679537; doi:10.1016/j.stemcr.2023.09.002)
Supplement: Document S1. Figures S1–S7 and Tables S2 and S4 [file mmc1.pdf]

Stem Cell Reports, Volume *18*

Supplemental Information

Human model of primary carnitine deficiency cardiomyopathy reveals ferroptosis as a novel mechanism

Malte Loos, Birgit Klampe, Thomas Schulze, Xiaoke Yin, Konstantinos Theofilatos, Bärbel Maria Ulmer, Carl Schulz, Charlotta S. Behrens, Tessa Diana van Bergen, Eleonora Adami, Henrike Maatz, Michaela Schweizer, Susanne Brodesser, Boris V. Skryabin, Timofey S. Rozhdestvensky, Sara Bodbin, Konstantina Stathopoulou, Torsten Christ, Chris Denning, Norbert Hübner, Manuel Mayr, Friederike Cuello, Thomas Eschenhagen, and Arne Hansen

Supplementary data file

Supplementary figures

Supplementary Figure 1

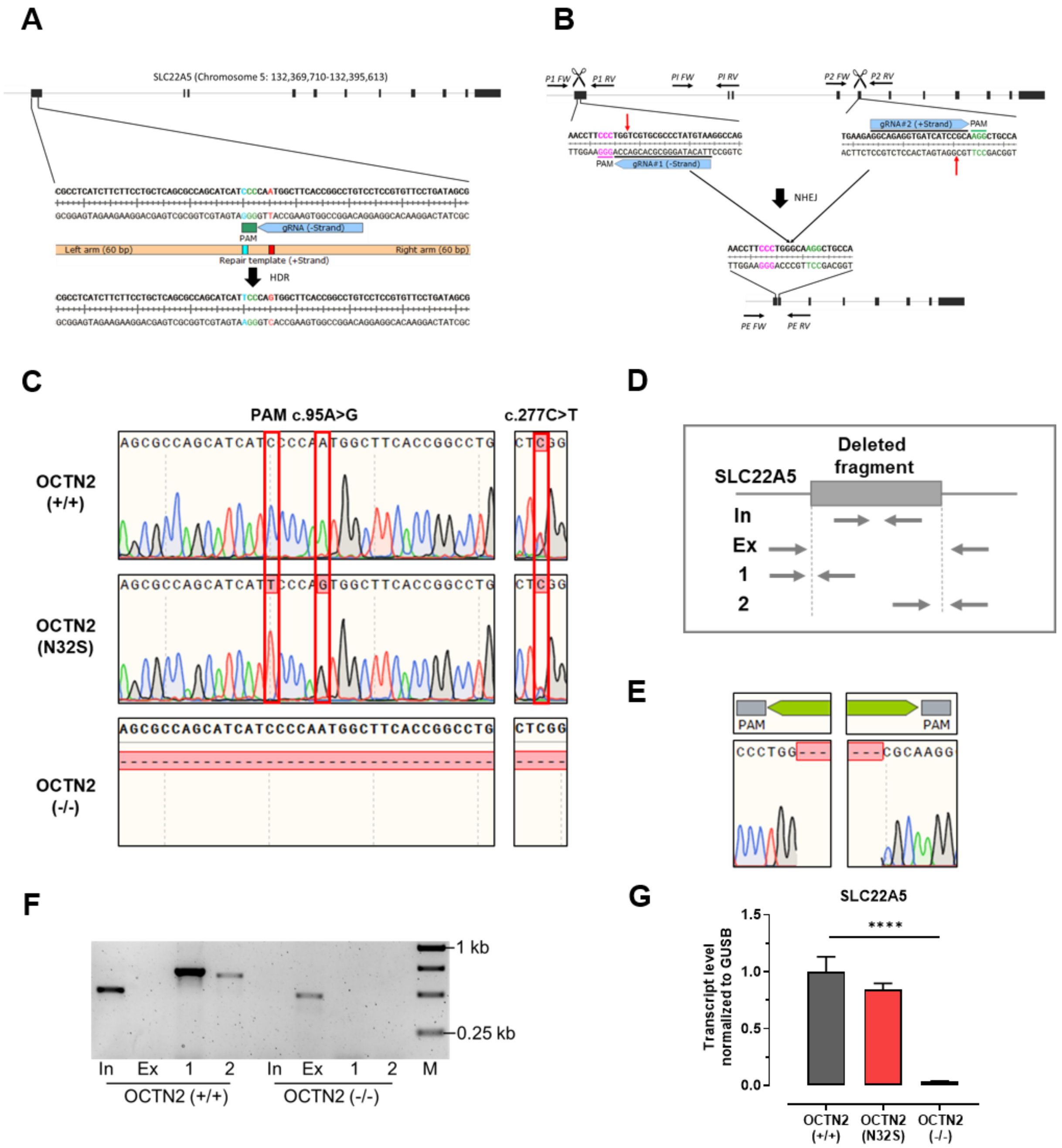

Supplementary Figure 1: Genotype characterization of CRISPR/Cas9-edited hiPSCs. A: Schematic overview of CRISPR/Cas9 strategy for OCTN2 (N32S) generation. A ssODN containing the desired point mutation was co-transfected with CRISPR components to introduce the mutation c.95A>G, p.N32S in exon1 of the SLC22A5 gene. Depicted is the gRNA target site and the predictive DNA sequence after successful integration of the repair template. **B**: Schematic overview of the CRISPR/Cas9 strategy for OCTN2 (-/-) generation. Two gRNA were co-transfected to induce simultaneous cutting and a large deletion in the SLC22A5 gene.

Depicted are the gRNA target sites and the predicted DNA sequence after successful editing. Red arrows indicate the predicted Cas9 cutting sites. Black arrows indicate the primer binding sites for PCR validation. P1: Primer target region gRNA1; P2: Primer target region gRNA2; PI: Primer internal; PE: Primer external; Pink: PAM1; Green: PAM2. **C**: Sanger sequencing traces of OCTN2 (+/+) and derived single cell clones for OCTN2 (N32S) and OCTN2 (-/-) genotypes. The red boxes indicate the silent PAM- and c.95A>G mutation and the heterozygous silent c277C>T mutation in the OCTN2 (N32S) clone. **D**: Schematic overview of primer localization in relation to the deleted fragment in the *SLC22A5* gene. **E**: Sanger sequencing trace of OCTN2 (-/-) PCR product amplified with external primers. **F**: Gel electrophoresis of PCR products of OCTN2 (+/+) and OCTN2 (-/-) hiPSC clones; M = 1 kb DNA standard marker. Depicted are the binding sites of both gRNA. **G**: qPCR analysis of *SLC22A5* transcripts for all genotypes.

Supplementary Figure 2

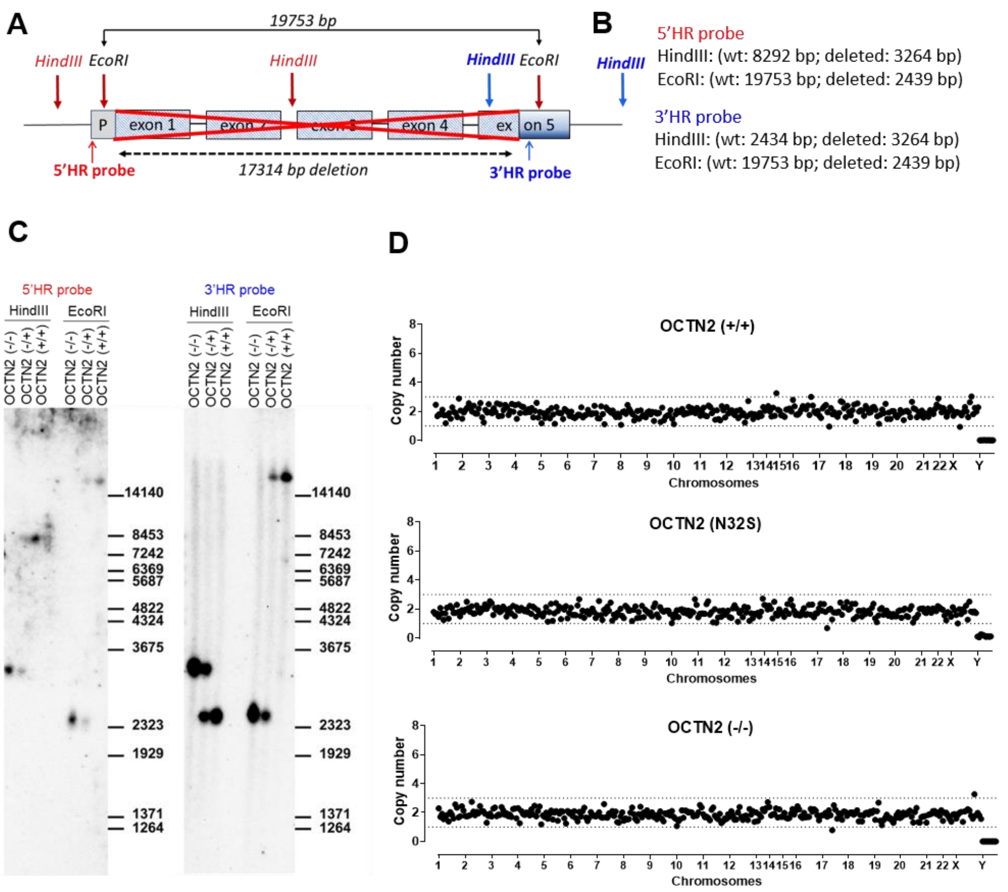

Supplementary Figure 2. **Southern blot and Nanostring analysis of genomic DNA from OCTN2 (+/+), OCTN2 (-/+) and OCTN2 (-/-) hiPSC. A:** Schematic presentation of Southern blot probes hybridization position. HindIII and EcoRI enzymes were used for enzymatic digestion of genomic DNA samples. **B**: Fragment size prediction of 5' HR and 3'HR hybridization after HindIII or EcoRI restriction enzyme digest. **C**: Southern blot analysis of OCTN2 (+/+), OCTN2 (-/+) and OCTN2 (-/-) hiPSC. **D:** Nanostring karyotype analysis of hiPSC master cell bank samples. OCTN2 (+/+), OCTN2 (N32S) and OCTN2 (-/-).

Supplementary Figure 3

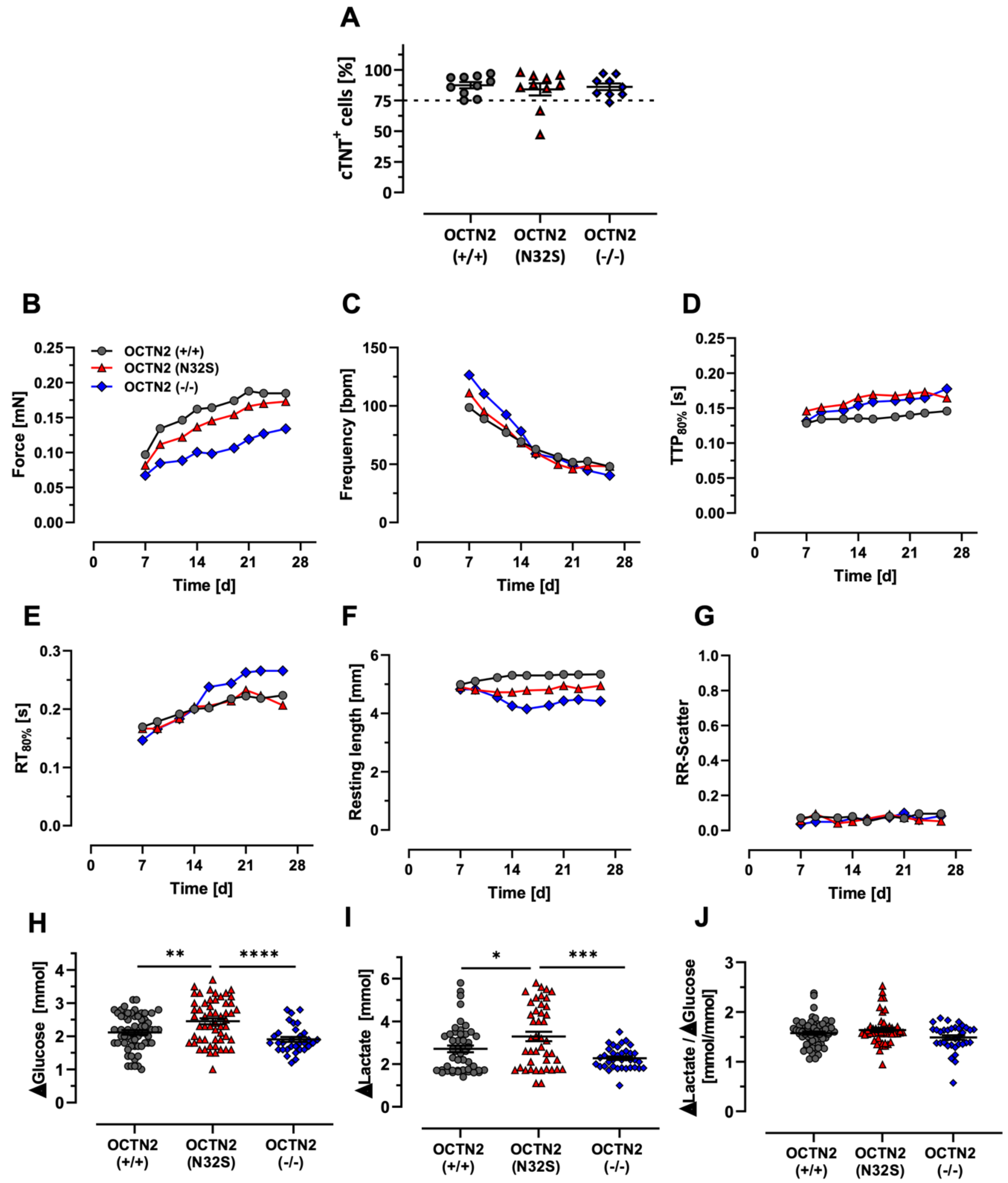

Supplementary Figure 3. Characterization of cardiomyocyte differentiations, contractile and metabolic parameters. A: FACS analysis of cardiac troponin T positive cells in the differentiated cell population. Dashed line (75%) indicates threshold value for EHT generation. OCNT2 (+/+): n=10, OCTN2 (N32S): n=10, OCTN2 (-/-): n=9 differentiation batches, data are expressed as mean±SEM, 1-way ANOVA followed by Bonferroni's post-test for multiple comparisons, ****$p<0.0001$. **B-G:** Effect of OCTN2 genotype on force, frequency, contraction time (TTP80), relaxation time (RT80), resting length and RR-Scatter (parameter of irregularity) of spontaneous beating EHTs between day 7 and day 26. OCNT2 (+/+): n=152 EHTs from 9 batches, OCTN2 (N32S): n=108 EHTs from 7 batches, OCTN2 (-/-): n=91 EHTs from 5 batches, data are expressed as EHT batch mean±SEM. **H:** Difference in Glucose- and **I:** Lactate concentration of EHT culture media (Δglucose = glucose concentration at baseline minus

glucose concentration after 24 h of incubation, Δlactate = lactate concentration at baseline minus lactate concentration after 24 h of incubation). **J:** ΔLactate of EHT culture media divided by Δglucose. OCNT2 (+/+): n=59 EHTs from 5 batches, OCTN2 (N32S): n=51 EHTs from 4 batches, OCTN2 (-/-): n=28 EHTs from 4 batches. 1-way ANOVA followed by Bonferroni's post-test for multiple comparisons, *$p<0.05$, **$p<0.01$, ***$p<0.001$, ****$p<0.0001$. One data point represents one independent EHT. Data are expressed as mean±SEM. Related to Figure 1 and 2.

Supplementary Figure 4

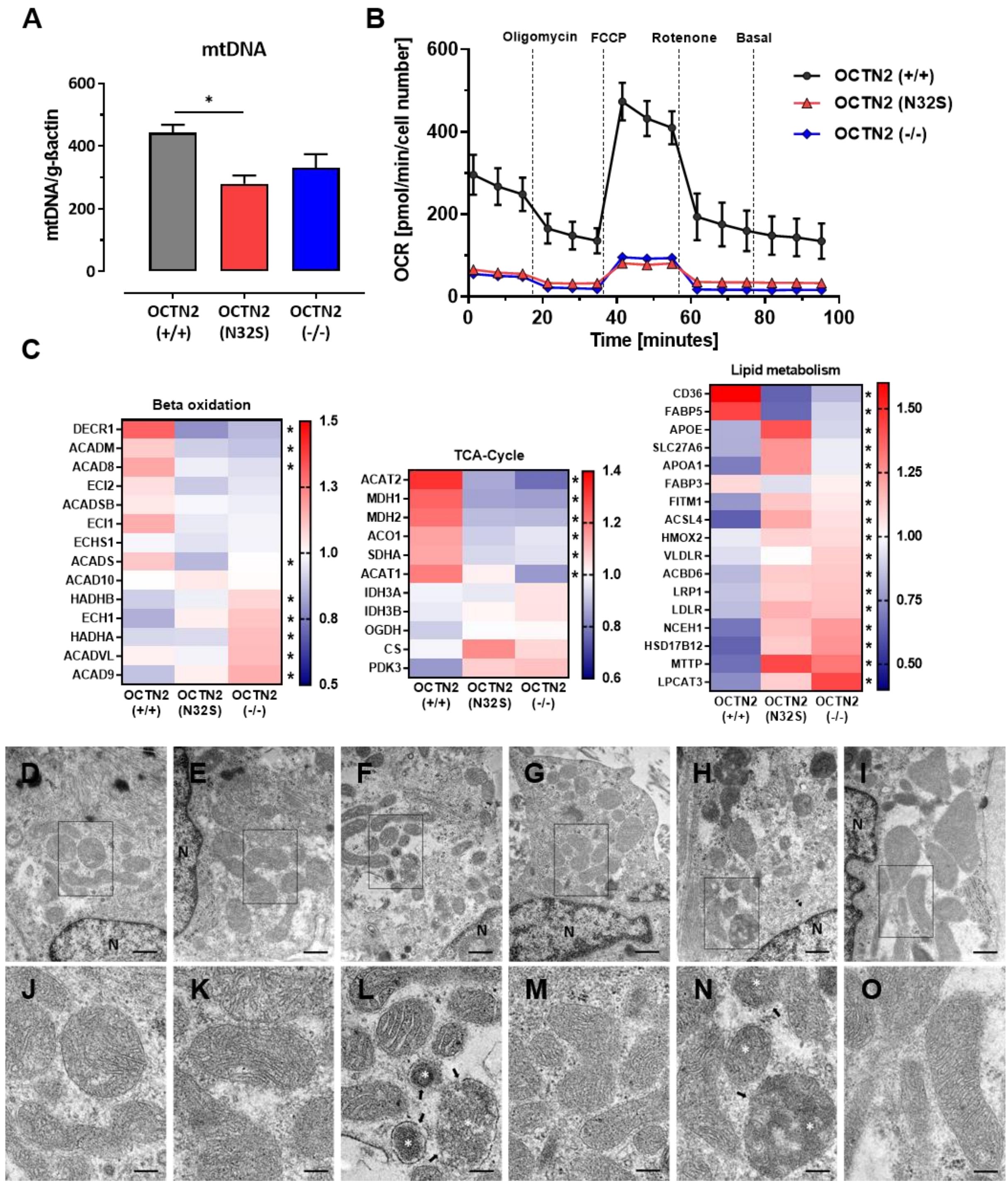

Supplementary Figure 4. A: Effect of OCTN2 genotype on mitochondrial DNA analyzed by quantitative PCR. MtDNA was normalized to nuclear encoded globular actin (g-actin). OCNT2 (+/+): n=5 EHTs from 1 batch, OCTN2 (N32S): n=7 EHTs from 1 batch, OCTN2 (-/-): n=7 EHTs from 1 batch. 1-way ANOVA followed by Bonferroni's post-test for multiple comparisons, *p<0.05. Data are expressed as mean±SEM. **B**: Oxygen consumption rate in OCNT2 (+/+), (N32S) and (-/-). Mean ± SEM, n=2 biological replicates (each biological replicate represents the average of 12 wells of a 96 well Seahorse plate), Mann-Whitney U test, *p<0.05. **C:** Clustering analysis of proteins related to metabolic pathways. Heatmaps display the relative abundance of proteins involved in the electron transport chain (ETC), beta-oxidation and lipid metabolism. OCNT2 (+/+): mean of 10 EHTs from 1 batch; OCTN2 (N32S): mean of 10 EHTs from 1 batch; OCTN2 (-/-): mean of 10 EHTs from 1 batch. Protein levels are depicted as a

color code ranging from blue (low abundance) to red (high abundance). Kruskal-Wallis test, * indicates statistically significant difference of OCTN2 (+/+) against OCTN2 (N32S) or OCTN2 (-/-). **Signs of Ferroptosis EM Transmission electron microscopy.** D + E: (+/+), F + G: OCTN2 (N32S), H + I: OCTN2 (-/-). D, F and H untreated, E, G and I supplemented with carnitine (2mM). J to O: enlargements of D to I, respectively. Mitochondria of OCTN2 mutants (F, L) and knock-out cells (H, N) show morphological features indicating ferroptosis like increased membrane matrix density and absent cristae (* in L, N), rupture and fragmentation of the outer membrane (arrows in L, N). Nuclear morphology did not display any changes (N). Scale bars 500nm D to I and 200nm J to L. Related to Figure 3 and 5.

Supplementary Figure 5

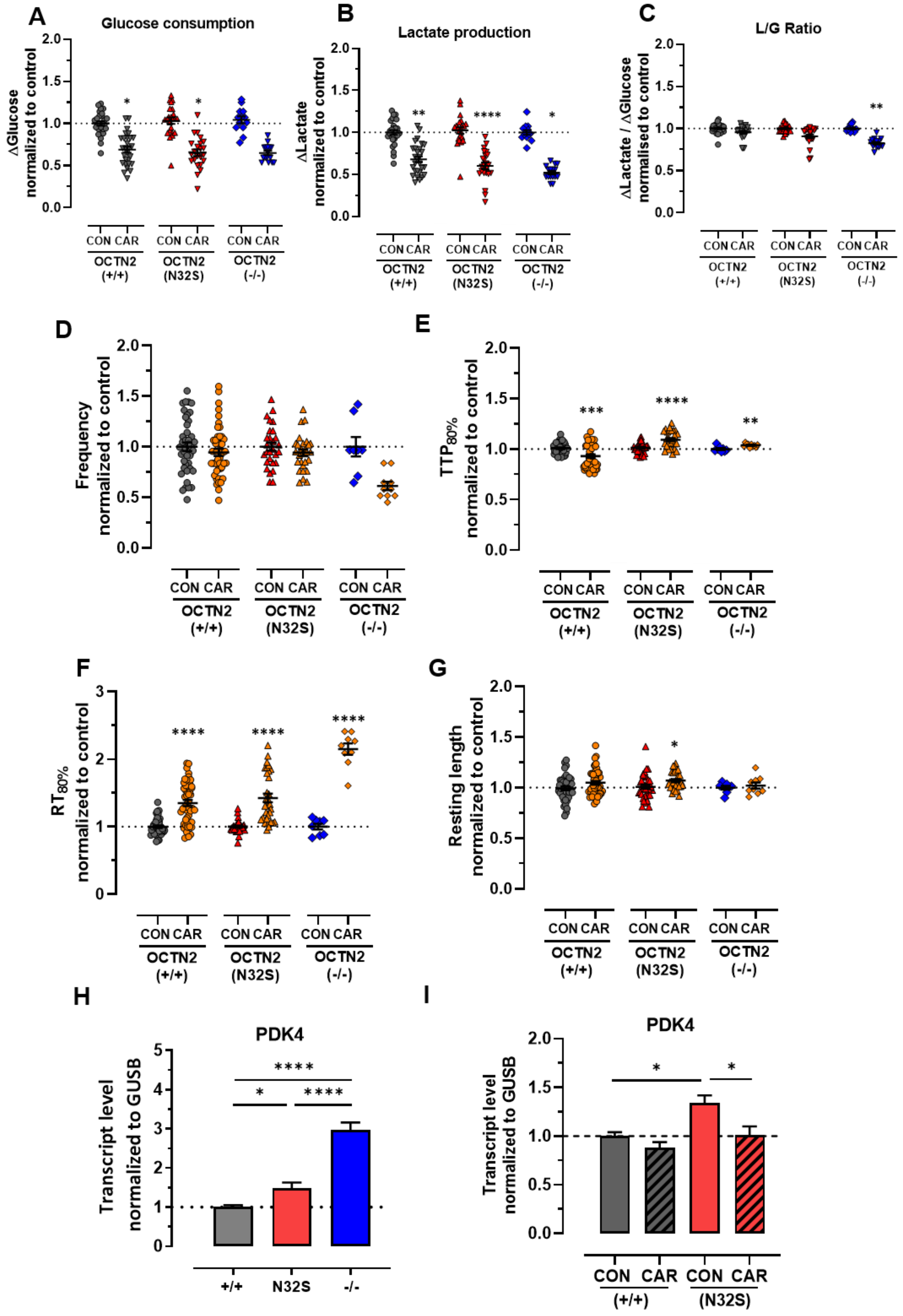

Supplementary Figure 5. A-C: Effect of carnitine supplementation on glucose consumption and lactate production, contractile parameters and PDK4 mRNA expression. A: ΔGlucose (ΔGlucose= Glucose concentration at baseline minus glucose concentration after 24 hours of incubation); **B**: ΔLactate (ΔLactate= Lactate concentration after 24 hours of incubation minus lactate concentration at baseline). Nested t-test vs CON, **p<0.01, ***p<0.001, ****p<0.0001. OCNT2 (+/+) control: n=27 EHTs from 3 batches. OCNT2 (+/+) + carnitine (2 mM): n=28 EHTs from 3 batches. OCTN2 (N32S) control: n=23 EHTs from 3 batches. OCTN2 (N32S) + carnitine (2 mM): n=23 EHTs from 3 batches, OCTN2 (-/-) control: n=13 EHTs from 3 batches, OCTN2 (-/-) + carnitine (2 mM): n=16 EHTs from 3 batches. Data are expressed as mean±SEM. **D-G: Effect of carnitine supplementation on spontaneous beating EHTs at the last day of treatment (Day 33-42). D**: Frequency, **E**: Time to peak, **F**: Relaxation time and **G**: Resting length. Values were normalized to last day of treatment of untreated control. Student's t-test vs CON, **p<0.01, ***p<0.001, ****p<0.001. OCNT2 (+/+) control: n=54 EHTs from 4 batches. OCNT2 (+/+) + carnitine (2 mM): n=49 EHTs from 4 batches. OCTN2 (N32S) control: n=36 EHTs from 3 batches, OCTN2 (N32S) + carnitine (2 mM): n=33 EHTs from 3 batches, OCTN2 (-/-) control: n=9 EHTs from 1 batch, OCTN2 (-/-) + carnitine (2 mM): n=9 EHTs from 1 batch. Data are expressed as mean±SEM. **H: Effect of OCTN2 genotype on PDK4 mRNA expression by quantitative RT-PCR analysis. Gene expression was normalized to GUSB over OCTN2 (+/+) control.** OCNT2 (+/+): n=8 EHTs from 2 batches, OCTN2 (N32S): n=8 EHTs from 2 batches; OCTN2 (-/-): n=6 EHTs from 2 batches. 1-way ANOVA plus Bonferroni's post-test for multiple comparisons, *p<0.05, ****p<0.0001. Data are expressed as mean±SEM. **I: Effect of carnitine supplementation on PDK4 mRNA expression.** OCNT2 (+/+) and OCTN2 (N32S) EHTs were treated over the entire culture time harvested on day 42. Gene expression was normalized to GUSB over OCTN2 (+/+) control. n=7 EHTs per genotype and carnitine treatment from 1 batch. 2-way ANOVA followed by Bonferroni's post-test for multiple comparisons, *p<0.05. Data are expressed as mean±SEM. Related to Figure 4.

Supplementary Figure 6

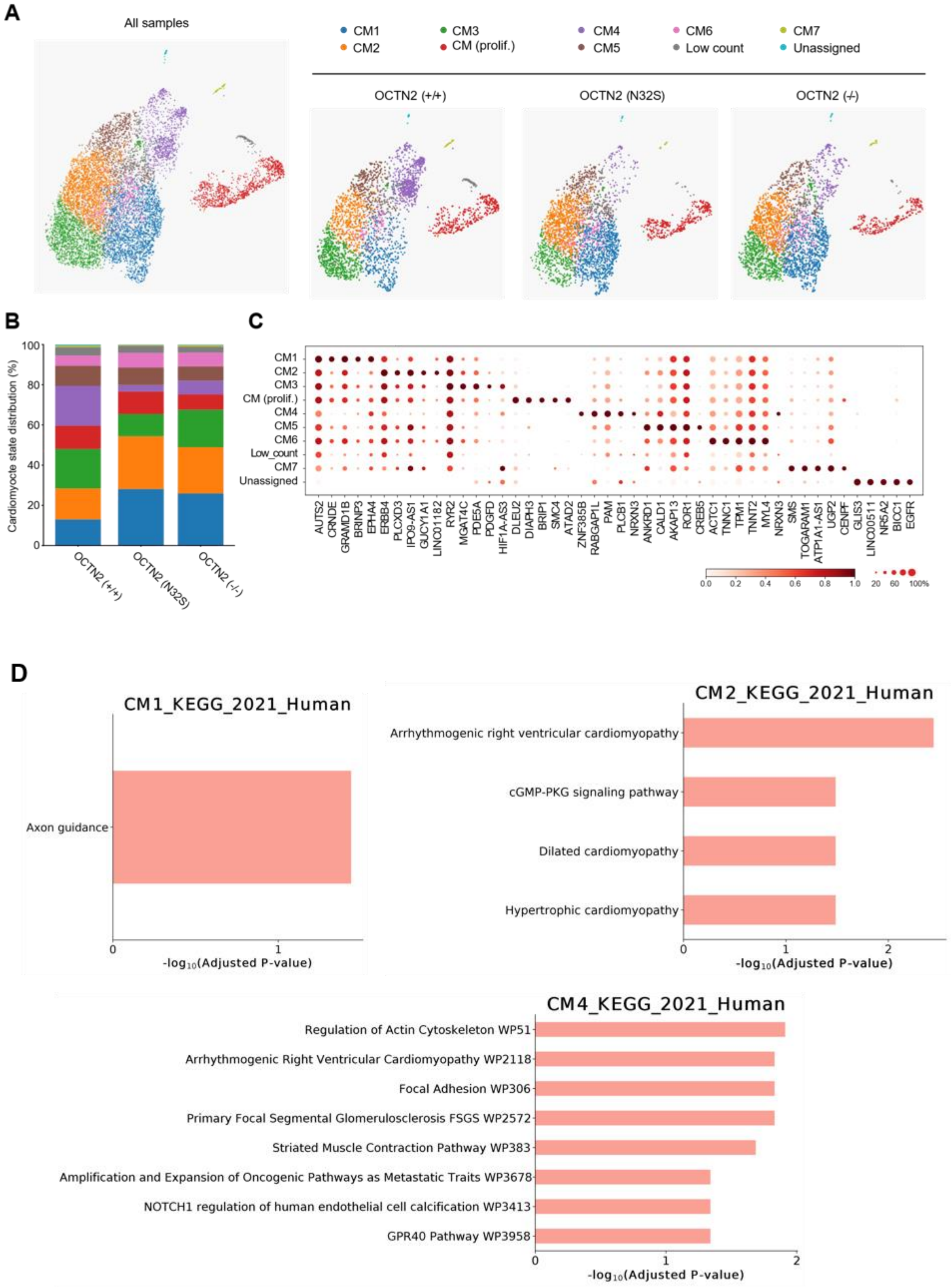

Supplementary Figure 6. Cardiomyocyte and fibroblast subcluster analysis in OCTN2 genotypes. A: Representative UMAP plot after single-nucleus RNA sequencing of all samples and individual

genotypes. **B:** Percentage of cardiomyocyte subcluster per genotype. **C:** Dot plot graph showing the relative expression of upregulated genes per cell cluster. Expression levels are depicted as a color code ranging from light red (low expression) to dark red (high expression) as mean of log2 fold of expression. **D:** Enrichment analysis of significantly upregulated genes from cardiomyocyte subclusters CM1, CM2, CM4. Related to Figure 6.

Supplementary Figure 7

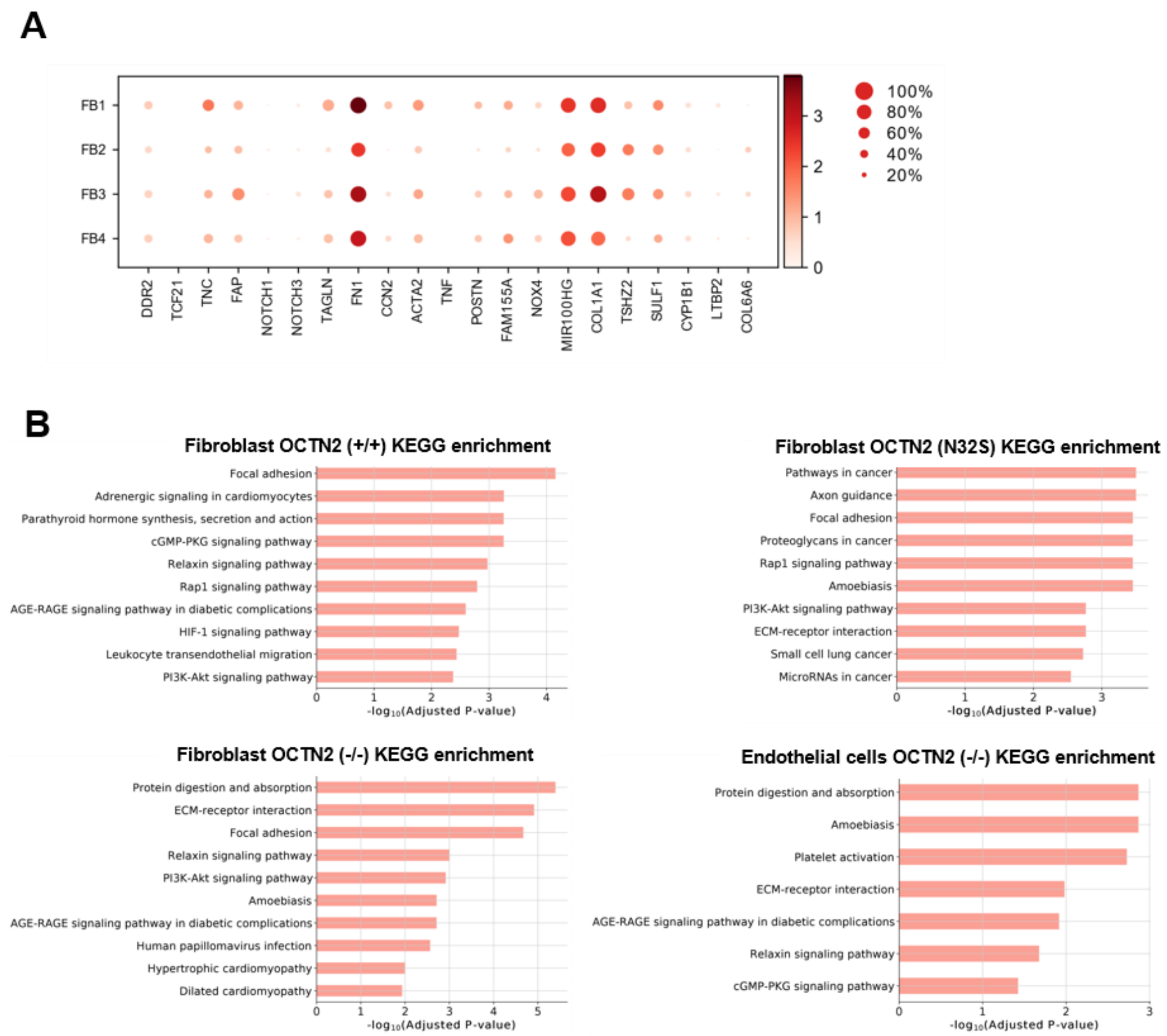

Supplementary Figure 7. Fibroblast and endothelial cell subcluster analysis in OCTN2 genotypes. A: Dot plot graph showing the relative expression of specific marker genes related fibroblast activation in fibroblast states. Unscaled expression levels are depicted as a color code ranging from light red (low expression) to dark red (high expression) as mean of log2 fold of expression. **B:** Enrichment analysis of significantly upregulated genes from fibroblast and endothelial cell subclusters. OCTN2 (+/+) and OCTN2 (N32S) endothelial cell and all myeloid subclusters did not reveal significant enriched pathways. Related to Figure 6 and 7.

Supplementary tables

Supplementary table 2. Composition of stem cell related culture media, related to Figure 1

**Medium**	**Components**	
Conditioned Medium (CDM)	DMEM/F12 without glutamine<br>1% (v/v) Non-essential amino acids<br>1% (v/v) L-glutamine<br>0.5% (v/v) Penicillin/streptomycin<br>3.5 µL/500 mL 2-Mercaptoethanol<br>20% (v/v) Knockout serum replacement<br>10 ng/mL Basic fibroblast growth factor (bFGF)	After incubation on mitotically inactivated mouse embryonic fibroblasts (strain CF-1) for 24 hours, CDM medium was collected and sterile filtered (0.1 µm filter). Fresh bFGF (30 ng/mL) was supplemented directly before use.
FTDA	DMEM/F-12 without glutamine<br>2 mM L-glutamine<br>0.1% (v/v) Lipid mix<br>5 mg/L Transferrin<br>5 µg/L Selenium<br>0.1% (v/v) Human serum albumin	5 µg/mL Insulin<br>2.5 ng/mL Activin-A<br>30 ng/mL bFGF<br>50 nM Dorsomorphin<br>0.5 ng/mL TGFß1
Mesoderm induction medium	RPMI 1640<br>4 mg/mL Polyvinyl alcohol<br>10 mM HEPES<br>0.05% (v/v) Human serum albumin<br>250 µM Phosphoascorbate<br>5 mg/l Transferrin	5 µg/l Selenium<br>0.1% (v/v) Lipid mix<br>10 µM Y-27632<br>3 ng/mL Activin-A<br>10 ng/mL BMP4<br>5 ng/mL bFGF
EB formation medium	FTDA<br>4 mg/mL Polyvinyl alcohol<br>10 µM Y-27632	
Cardiac differentiation medium I	RPMI 1640<br>10 mM HEPES<br>0.5% (v/v) Penicillin/streptomycin<br>0.05% (v/v) Human serum albumin<br>250 µM Phosphoascorbate	5 mg/L Transferrin<br>5 µg/L Selenium<br>0.1% (v/v) Lipid mix<br>1 µM Y-27632<br>1 µM XAV-939
Cardiac differentiation medium II	RPMI 1640<br>2% (v/v) B27 plus insulin<br>10 mM HEPES<br>0.5% (v/v) Penicillin/streptomycin	500 µM 1-Thioglycerol<br>1 µM Y-27632<br>1 µM XAV-939
Cardiac differentiation medium III	RPMI 1640,<br>2% (v/v) B27 plus insulin<br>10 mM HEPES	0.5% (v/v) Penicillin/streptomycin<br>500 µM 1-Thioglycerol<br>1 µM Y-27632

Supplementary Table 2ff. Composition of stem cell related culture media

Medium	Components	
EHT casting medium	DMEM<br>1% (v/v) Penicillin/streptomycin	2 mM L-Glutamine<br>10% (v/v) Horse serum (heat inactivated)
EHT culture medium	DMEM<br>1% (v/v) Penicillin/streptomycin<br>10% (v/v) Horse serum	10 µg/mL Insulin<br>33 µg/mL Aprotinin<br>0.2 mM Transexamic acid
Fatty acid medium	DMEM (without Glucose)<br>1% (v/v) Penicillin/streptomycin<br>10 µg/mL Insulin<br>33 µg/mL Aprotinin<br>0.2 mM Transexamic acid	50 ng/mL Hydrocortisone<br>0.5 ng/mL T_3<br>0.2 mM L-Glutamine<br>50 µM L-Carnitine hydrochloride<br>1 mg/mL Linoleic Acid-Oleic Acid-Albumin
Tyrode's solution	120 mM NaCl<br>5.4 mM KCl<br>1 mM $MgCl_2$ x 6·H2O<br>0.4 mM NaH_2PO_4	22.6 mM $NaHCO_3$<br>5 mM Glucose<br>0.05 mM Na_2EDTA<br>25 mM HEPES (pH 7.4, in PBS)
Mastermix EHT casting	hiPSC-CM<br>EHT casting medium<br>2x DMEM<br>0.1% Y-27632<br>Fibrinogen	$1.0x10^6$ per EHT<br>97.68 µL per EHT<br>6.125 µL per EHT<br>0.11 µL per EHT<br>2.78 µL per EHT

Supplementary Table 4. Primary antibodies used for flow cytometry/FACS, related to Supplementary Figure 3

Antibody	Dilution	Supplier and Cat#
Anti-cardiac troponin T-FITC	1:50	Miltenyi Biotec, 130-119-674
REA Control (I)-FITC	1:50	Miltenyi Biotec, 130-120-709
Rat Anti-Human SSEA-3 Antibody, PE Conjugated	1:50	BD Biosciences, 560237
Rat IgM, κ Isotype Control Antibody, PE Conjugated	1:50	BD Biosciences, 553943

Experimental procedures

Nucleofection

The AmaxaTM P3 Primary Cell 4D-Nucleofector X Kit L (Lonza) was used for delivery of the CRISPR/Cas9 ribonucleoprotein (RNP) complex into hiPSCs. A working cell bank aliquot of the control hiPSC (passage 25-30) was cultured for at least 2 passages on a 6-well plate to reach 60-70% confluency on the day of nucleofection. HiPSCs were incubated with the apoptosis inhibitor Y-27632 (10 µM) two hours prior to nucleofection. The fluorescence-labeled tracrRNA-ATTO 550 (IDT) was used to monitor the electroporation efficiency. The tracrRNA oligos and the CRISPR-Cas9 crRNA (IDT) oligos were resuspended in RNAse-free IDTE Buffer (IDT) to a final stock concentration of 100 µM. For gRNA duplex formation, 5 µL of crRNA (100 µM) were annealed with 5 µL tracrRNA (100 µM), incubated for 5 min at 95 °C and cooled down to room temperature. For formation of the RNP- complex, 5 µL of the gRNA duplex were mixed with 5 µL Cas9 protein (61 µM, IDT) and incubated for 1.5 hours at room temperature under light protection. For the knockout approach 5 µL of the second gRNA duplex were added to the suspension in addition. To prepare the nucleofector solution, 82 µL P3 reagent and 18 µL supplement reagent (Lonza) were mixed per reaction according to the instruction of the AmaxaTM P3 Primary Cell 4D-Nucleofector X Kit L (Lonza). HiPSCs were washed twice with PBS buffer and dissociated into single cells with 1 mL accutase solution (Sigma) per well at 37 °C. The dissociation was stopped by adding 1 mL FTDA medium per 6-well. The hiPSCs were resuspended in the media by gentle pipetting and centrifuged for 2 min at 200xg. $8x10^5$ hiPSCs were used in 100 µL nucleofector solution per electroporation reaction.

Single-stranded DNA oligonucleotide (ssODN) repair template oligos were resuspended in IDTE Buffer (IDT) to a stock concentration of 100 µM. 1 µL of ssODN repair template (100 µM) and 4 µL of RNP-complex were mixed with the hiPSC solution by gently pipetting, incubated for 5 min at room temperature and transferred to the nucleofection cuvette. Additionally, 1 µL Alt-R Cas9 Enhancer (100 µM, IDT) was added to the solution to promote transfection efficiency. The nucleofection cuvette was placed in the 4D-Nucleofector (Lonza) and hiPSCs were nucleofected by using the program CA137. After nucleofection, the cuvette was incubated for 5 min under cell culture conditions. Subsequently, hiPSCs were seeded in conditioned medium supplemented with Y-27632 (10 µM) and bFGF (30 ng/ml) (Supplementary Table 2) on a Matrigel-coated 24-well plate for 72 hours at 37 °C.

Subcloning and off-target analysis

72 hours after nucleofection, hiPSC were dissociated with Accutase and seeded in conditioned medium with Y-27632 (10 µM) and bFGF (30 ng/mL) at low seeding densities of 100, 250, 750, 1000 hiPSCs per well (10 cm^2) in a Matrigel-coated 6-well plate. In addition, the remaining nucleofected hiPSCs were seeded at a higher density of $5x10^5$ cells per well (10 cm^2) in a Matrigel-coated 6-well plate. Low-density hiPSC seedings were expanded for 9 to 10 days under daily conditioned medium change until clonal hiPSC colonies reached a size appropriate to pick. HiPSC cultures were incubated with conditioned medium with Y-27632 (10 µM) for 2 hours and sterile 100 µL-pipette tips were used to carefully scrape individual colonies from the 6-well plate and transfer them to Matrigel-coated 48-well plates into individual wells. 30 to 50 clones were picked per transfection approach and were sub-cultivated for 3-4 more days before they reached confluency and were splitted with a ratio of 1:2 into two 48-well copy plates. Colonies were again expanded with daily medium change until they reached confluency. One of the copy plates was used for cryo-preservation, while the second plate was used for DNA isolation. Cryopreservation was performed in 90% FBS and 10% DMSO.

QIAcube HT System (Qiagen) and QIAamp 96 DNA QIAcube HT kit (Qiagen) were used for DNA isolation according to the manufacturer's instructions. Cryotubes of successfully edited hiPSC clones were thawed and expanded for master cell bank and working cell bank. Ten most likely off-targets were predicted (*in silico* tool IDT, Custom Alt R CRISPR-Cas9 gRNA software and CRISPOR software). Corresponding PCR primers were designed and PCR products were analysed by 1% (w/v) agarose electrophoresis and Sanger sequencing. Off-target primer sequences can be found in Supplementary Table 3, sheet 2.

Karyotyping

Karyotype analysis was performed using the nCounter Human Karyotype Panel (Nanostring Technologies) according to the manufacturer's instructions with 250 µg DNA as starting material. The nCounter CNV Collector Tool software (Nanostring) was used for analysis.

Glucose- and lactate measurement

Glucose- and lactate concentrations were measured in EHT cell culture media by blood gas analysis. Samples were collected at baseline and after 24 hours of incubation in EHT medium on day 21 of culture and stored at -20°C. The blood gas analysis instrument ABL90 FLEX Analysator (Radiometer) was used to determine the metabolite concentrations by injecting the supernatant (0.5 mL) into the instrument with a 1 mL syringe.

Fatty acid- and carnitine supplementation

EHTs were cultured in serum-containing EHT culture medium until force values reached their plateau phase approximately at day 21-28 after generation. Then, EHTs were transferred to a serum- and glucose-free DMEM medium containing linoleic acid- and oleic acid-albumin (Sigma). The detailed serum-free fatty acid medium composition is shown in Supplementary Table 2. Contraction analysis was done daily after 2 hours of medium incubation for 4 days. Supplementation with carnitine was conducted by adding 2 mM L-carnitine hydrochloride (Sigma) to the EHT medium over the entire time of tissue cultivation.

Transmission electron microscopy

EHTs were transferred into a 24-well plate containing Tyrode's solution (Supplementary Table 2) with 1.8 mM Ca^{2+} and 30 mM butanedione monoxime (BDM) for 3-4 hours until EHTs completely stopped contraction. For fixation, EHTs were shifted into 4% paraformaldehyde (PFA, Thermo Scientific) in 0.1 M phosphate buffer containing 1% glutaraldehyde (Science Services) overnight at 4 °C. Samples were rinsed three times in 0.1 M sodium cacodylate buffer (pH 7.2–7.4) and osmicated using 1% osmium tetroxide in cacodylate buffer. Following osmication, the samples were dehydrated using ascending ethanol concentrations, followed by two rinses in propylene oxide. Infiltration of the embedding medium was performed by immersion in a 1:1 mixture of propylene oxide and Epon (Science Services, Germany), followed by neat Epon and hardening at 60 °C for 48 hours. For light microscopy, semi-thin sections (0.5 µm) with longitudinal orientation were mounted on glass slides and stained for 1 minute with 1% toluidine blue. For electron microscopy, ultra-thin sections (60 nm) were cut and mounted on copper grids and stained using uranyl acetate and lead citrate. Sections were examined and photographed using an EM902 (Zeiss) electron microscope equipped with a TRS 2K digital camera (A. Tröndle, Moorenweis, Germany).

Analysis of acylcarnitines and ceramides

EHT cells were homogenized in Milli-Q water (approximately $1.0x10^6$ hiPSC-CM per 100 µl) using the Precellys 24 Homogenisator (Peqlab). The protein content of the homogenate was routinely determined using bicinchoninic acid. Levels of acylcarnitines and ceramides in the cell homogenates were determined by Liquid Chromatography coupled to Electrospray Ionization Tandem Mass Spectrometry (LC-ESI-MS/MS).

Acylcarnitines

Acylcarnitines were derivatized to butyl esters and using a procedure previously described (Giesbertz *et al*, 2015) with several modifications: To 100 µL of cell homogenate 750 µL of extraction solution (methanol (containing 0.005% 3,5-di-*tert.*-4-butylhydroxytoluol)/Milli-Q water/chloroform 4:1:1 (v/v/v)) and 20 µL of an internal standard mixture, containing deuterated acylcarnitines (Lyophilized Internal Standard MassChrom Amino Acids and Acylcarnitines from Dried Blood, Chromsystems, reconstituted in 2.5 ml, then 1:5 diluted), were added. After thorough mixing and centrifugation (16,100 RCF, 10 min, 4 °C), the supernatant was transferred to a new tube. The residue was re-extracted with 750 µL of extraction solution. The supernatants were pooled and dried under a stream of nitrogen. The evaporated extracts were treated with 200 µL of freshly prepared derivatization solution (*n*-butanol/acetyl chloride 95:5 (v/v)). After incubation for 20 min at 60 °C in a ThermoMixer (Eppendorf) at 800 rpm, the samples were again dried under nitrogen. After the addition of 100 µL of methanol/water 3:1 (v/v) and centrifugation (16,100 RCF, 10 min, 4 °C), 80 µL of supernatant were transferred to autoinjector vials. LC-ESI-MS/MS analysis was performed as previously described (Aravamudhan *et al*, 2021). The LC chromatogram peaks of butyl esters of endogenous acylcarnitines and internal standards were integrated using the MultiQuant 3.0.2 software (SCIEX). Endogenous acylcarnitine species were quantified by normalizing their peak areas to the peak area of the internal standards. These normalized peak areas were normalized to the protein content of the sample.

Ceramides

To 50 µL of cell homogenate 50 µL of Milli-Q water, 750 µL of methanol/chloroform 2:1 (v/v), and internal standard (127 pmol ceramide 12:0, Avanti Polar Lipids) were added. Lipid extraction and LC-ESI-MS/MS analysis were performed as previously described (Schwamb *et*

al, 2012). The LC chromatogram peaks of endogenous ceramide species and the internal standard ceramide 12:0 were integrated using the MultiQuant 3.0.2 software (SCIEX). Endogenous ceramide species were quantified by normalizing their peak areas to the peak area of the internal standard ceramide 12:0. These normalized peak areas were normalized to the protein content of the sample.

Actions potential

Actions potential (AP) measurements in EHT were performed with standard sharp microelectrode as described previously (Lemoine *et al*, 2018). The EHTs were transferred from the 24-well EHT culture plate into the AP measuring chamber by cutting the silicone posts and were fixed with needles in recording chamber. All measurements were done with tissues continuously superfused with Tyrode´s solution (NaCl 127 mM, KCl 5.4 mM, $MgCl_2$ 1.05 mM, $CaCl_2$ 1.8 mM, glucose 10 mM, $NaHCO_3$ 22 mM, $NaHPO_4$ 0.42 mM, balanced with O_2-CO_2 [95:5] at 36 °C, pH 7.4). Microelectrodes had a resistance between 25 - 55 MΩ when filled with 2 M KCl. The signals were amplified by a BA-1s npi amplifier (npi electronic GmbH, Tamm, Germany). APs were recorded and analyzed using the Lab-Chart software (version 5, AD Instruments Pty Ltd., Castle Hill NSW, Australia). Take-of potential (TOP) was defined as the diastolic membrane potential directly before the upstroke.

Analysis of mitochondrial respiration

The Seahorse™ XF96 extracellular flux analyzer was used to assess mitochondrial respiration as previously described (Mosqueira et al, 2019), using the Mito Stress Kit (Agilent Technologies). Briefly, cryopreserved isogenic sets of hiPSC-CMs were seeded into Matrigel™-coated (BD #356235) XF96 well plates at a density of approximately 5000 cells/mm^2. HiPSC-CMs were cultured for 2 days in RPMI1640 (USBiological Life Sciences #R9010-01) supplemented with B-27 with insulin (LifeTechnologies #0080085-SA), 2 mM L-glutamine (Life Technologies #25030-081), 10% Fetal Bovine Serum (Gibco #16000044) and 0.6 mM CaCl2. After 2 weeks, medium was exchanged for XF basal medium (Agilent Technologies #102353), supplemented with 10 mM glucose (Sigma #G7528), 1 mM sodium pyruvate (Sigma #S8636) and 2 mM L-glutamine (Life Technologies #25030-081) 1h before the conduction of the assay. Selective inhibitors were sequentially injected during the measurements (1.5 µM oligomycin, 0.4 µM FCCP, 1 µM rotenone; Agilent Technologies), following the manufacturer's

instructions. The measured Oxygen Consumption Rate (OCR) values were normalized to the number of cells in each well, quantified by 1:400 Hoechst33342 staining (Sigma #B2261) in PBS (Gibco #14190-094) using fluorescence at 355 nm excitation and 460 nm emission in an automated imaging platform (CellaVista, Synentec).

Tandem Mass Tag (TMT)-based quantitative proteomic analysis

EHT harvesting for quantitative mass spectrometry

EHTs from hiPSC-CMs were cultured in EHT medium for 28 days before proteome analysis. EHTs were washed twice in warm PBS buffer and incubated with collagenase II solution (collagenase II (200 units per mL), HBSS minus Ca^{2+}/Mg^{2+}, HEPES (10 mM), Y-27632 (10 µM), and BTS (30 µM)) in a falcon tube for 3 hours. Dissociated hiPSC-CMs were gently titrated with a 1000 µL-pipette (Eppendorf) until the last cluster of cells was disaggregated, spun down for 5 min at 200xg and supernatant was removed. The pellet was frozen in liquid nitrogen and stored at -80 °C before subjection to quantitative proteome analysis.

Tissue protein extraction and digestion for mass spectrometry

Cell pellets were lysed in 50 mM Tris, 0.1% SDS, pH=8.8, with protease inhibitors. After centrifugation at 4 °C at 16,000xg for 10 min, the supernatant was transferred to new 1.5 mL tubes and protein concentration was measured using a BCA protein assay kit (Thermo Fisher Scientific). For each sample, 23 µg of proteins were denatured by the addition of 9 M urea, 3 M thiourea (final conc. 6 M urea, 2 M thiourea) and reduced by the addition of 100 mM DTT (final conc. 10 mM) followed by incubation at 37 °C for 1 hour, 240 rpm. The samples were then alkylated by the addition of 500 mM iodoacetamide (final conc. 50 mM) followed by incubation in the dark for 1h at room temperature. Pre-chilled (-20 °C) acetone (1:9 volume ratio) was used to precipitate the samples overnight at -20 °C. Samples were centrifuged at 16,000xg for 30 min at 4 °C and the supernatant was subsequently discarded. Protein pellets were dried using a vacuum centrifuge (Thermo Fisher Scientific, Savant SPD131DDA), re-suspended in 0.1M TEAB buffer, pH 8.2 (Sigma T7408), and 0.6 µg trypsin was added. The digestion was performed overnight at 37 °C, 240 rpm, and stopped by adding 10% TFA (final conc. 1%). C18 clean-up was performed using Agilent Bravo AssayMAP and the eluted peptides were dried using a vacuum centrifuge.

Sample labeling for mass spectrometry

The samples were resuspended in 0.1 M TEAB and a pooled sample was made by taking the same amount of proteins from each individual sample. Samples were labeled with Tandem Mass Tag (TMT) 11-plex reagent (Thermo Fisher Scientific) following the manufacturer's instructions. The pooled sample labeled with TMT-126 was used as an internal standard. The samples labeled with different tags of the 11-plex TMT were combined, dried, and resuspended in 300 µl of 0.1% TEA. Samples were further fractionated using high pH RP HPLC (Agilent 300Extend-C18 3.5um 4.6x150mm P/N 763973-902) and 16 fractions were collected for each TMT mixture. All fractions were dried and resuspended in LC solution (2% acetonitrile (ACN), 0.05% TFA).

Mass spectrometry analysis

Samples were injected and separated by a nanoflow HPLC (Ultimate 3000 RSLCnano, Thermo Fisher Scientific) on an EASY-Spray column (C18, 75 µm x 50 cm, 2 µm) using 2 hour LC gradient: 0-10 min, 4%-10%B; 10-75 min, 10%-30%B; 75-80 min, 30%-40%B; 80-85 min, 40%-99%B; 85-90 min, 99%B; 90-120 min, 4%B; A=0.1% FA in H2O and B= 0.1% FA, 80% ACN in H2O. The flow rate was 250 nl/min and column temperature was set at 45°C. The separated peptides were directly injected to an Orbitrap Fusion Lumos MS (Thermo Fisher Scientific) and analyzed using a synchronous precursor selection (SPS)-based MS3 method for TMT-labeled sample. Full MS spectra were collected on the Orbitrap with a resolution of 120,000 and scan range 375-1500 m/z. The most abundant ions were fragmented using CID and MS2 spectra were collected on a linear ion trap, with dynamic exclusion enabled. The 5 most abundant ions from every MS2 spectrum were selected and fragmented at the same time using HCD with collision energy 65% and MS3 spectra were collected on the Orbitrap with a resolution of 60,000 and a scan range of 110-500 m/z to measure the TMT reporter ions. The cycle time was set at 3 seconds.

Raw data were analyzed using Proteome Discoverer 2.4. The 16 fractions of each TMT mixtures were loaded as fractions and analyzed together. Each TMT tag was assigned to the correct sample and group. Data were searched against the human UniProt/SwissProt database (version 2020_01, 20365 protein entries). Trypsin was used as an enzyme and maximum 2 missed cleavage was allowed. The precursor mass tolerance was set at 10 ppm and fragment mass tolerance was set at 0.8 Da. Carbamidomethylation on cysteine and TMT 6plex on N-

terminal and lysine were used as static modifications. Oxidation on methionine was used as a dynamic modification. Reporter ions S/N were used for quantification. The quantification values were normalized to total peptide amount and scaled on controls (pooled sample labeled with TMT-126). The scaled abundance was exported for further analysis.

The dataset was first imputed to replace missing relative quantities to zeros when these were consistent among any of the examined phenotypes. In specific, when the percent of missing values in one examined phenotype exceeded 90% and the percentage of missing values for the other phenotypes was below 10% then the missing values of the examined phenotype were imputed to zeros. The relative quantities of the proteins were then scaled using log2 transformation. Next, the dataset was filtered to keep only proteins with less than 30% missing values, without considering the previously imputed missing values as missing. The remaining missing values were imputed using KNN-Impute method with k equal to 3. The limma package has been used to compare different phenotypes using the Ebayes algorithm and correcting for selected covariates. The initial p-values were adjusted for multiple testing using Benjamini-Hochberg method and a threshold of 0.05 was used for the adjusted p-values to infer statistically significant changes.

Pathway enrichment analysis of significant proteins

The bioinformatic webtool Webgestalt (Liao *et al*, 2019) was used for pathway enrichment analysis of KEGG terms. KEGG pathways of significantly enriched proteins (fold change ≥ 1.4) were inferred by Benjamini-Hochberg testing with a p-value threshold of 0.05 and a maximum number of 150 proteins per category. To visualize the samples based on their proteomic profiles, principal component analysis (PCA) was conducted, and samples were projected in a 2D space based on their 2 most significant principal components. Samples were color-coded based on their phenotype. Volcano plots for the visualization of differentially expressed proteins were generated in GraphPad PRISM.

Gene expression analysis, mitochondrial DNA quantification

Total RNA was extracted from samples and complementary DNA (cDNA) was generated by reverse transcription using the TRIzol and high-capacity cDNA reverse transcription kit (Applied Biosystems) according to the manufacturer's instructions. All experiments were performed using technical triplicates. The ΔΔCt method was used for calculation of relative

transcript expression levels. Primer sequences are listed in Supplementary Table 3, sheet 3. Gene expression of target genes was normalized to the reference transcripts of the housekeeping gene glucuronidase-beta (GUSB). The qPCR experiments were conducted with the AbiPrism 7900HT Fast Real-Time PCR System (Applied Biosystems) using HOT FIREPol EvaGreen qPCR Mix Plus (Solis BioDyne).

Quantification of mitochondrial DNA (mtDNA) was performed according to a qPCR protocol recently described by Ulmer et al. (Ulmer *et al*, 2018). In brief, genomic DNA (including mitochondrial DNA) was isolated by TRIzol extraction according to the manufacturer's instruction. DNA concentrations of each sample was adjusted to 16.5 µg/µL prior to the experiment by dilution. The mt-DNA content was quantified by normalizing gene expression values of the mitochondrially encoded NADH dehydrogenase- 1 (mt-ND1) and -2 (mt-ND2) to the nuclear-encoded globular actin (g-actin). Primer sequences were used as described (Ulmer *et al*, 2018; Burkart *et al*, 2016) and are listed in Supplementary Table 3, sheet 3.

Southern blot

Southern blot probe design.

The 5'HR probe was cloned using PCR primer pairs SLC_5HR1 (Supplementary Table 3, sheet 4), and PCR amplified using pairs SLC_5HR2. The 3'HR probe was cloned using PCR primer pairs SLC_3HR1, and PCR amplified using pairs SLC_3HR2 (Supplementary Table 3, sheet 4).

The Southern blot procedure was performed according to Skryabin et al .(Skryabin *et al*, 2020). HiPSC were thawed from an MCB aliquot and expanded to T25 flask format with 100% confluency. HiPSCs were washed with 5 mL PBS per flask and lysed in 1 mL standard lysis buffer containing 100 mM Tris-HCl (pH 8.5), 5 mM EDTA, 0.2% SDS, 200 mM NaCl, 100 g/mL Proteinase K (Roche) incubated overnight at 37 °C. DNA was extracted by phenol, phenol/chloroform extraction, precipitated in isopropanol and washed in 80% ethanol. DNA samples were dissolved in TE buffer. Approximately 10-15 µg of genomic DNA was digested with *Eco*RI, and *Hind*III restriction endonucleases, fractionated on 0.8% agarose gels and transferred to GeneScreen nylon membranes (NEN DuPont, USA). The membranes were hybridized with a ^{32}P-labeled 0,5-kb 5'HR probe containing sequences 5' to the deleted region (5'HR probe, Supplementary Figure 3) and washed with SSPE buffer (0.09 M NaCl, 5 mM NaH_2PO4, and 0.5 mM EDTA [pH 7.7]) and 0.5% sodium dodecyl sulfate at 65°C. Similarly,

membranes were hybridized and washed with a ^{32}P-labeled 1,2-kb 3'HR probe containing sequences 3' to the deleted region (3'HR probe, Supplementary Figure 3).

Single-nucleus RNA sequencing

EHTs were washed with PBS, detached from the PDMS posts, frozen in liquid nitrogen, and stored at -150 °C. Single-nucleus RNA sequencing (snRNA seq) was performed according to Litviňuková et al. (Litviňuková *et al*, 2020). In brief, single nuclei were isolated from frozen EHTs and purified by fluorescent-activated cell sorting (FACS). Nuclei were further processed using the Chromium Controller (10X Genomics) according to the manufacturer's protocol with a targeted nuclei recovery of 5,000 per reaction. 3' gene expression libraries were prepared according to the manufacturer's instructions of v3 Chromium Single Cell Reagent Kits (10X Genomics).

Sequencing data analysis

Bcl files were converted to Fastq files by using bcl2fastq. Each sample was mapped to the human reference genome GRCh38 (release Ens84) using the CellRanger suite (v.3.0.1). Mapping quality was assessed using the cellranger summary statistics; Empty droplets were identified by Emptydrops, implemented in the CellRanger workflow, and subsequently removed, while doublets were identified and filtered using Solo (Bernstein *et al*, 2020).

Downstream analysis was performed using the Python Scanpy v1.5.1 toolkit. Single nuclei were filtered for counts (300 ≤ n_counts ≤20,000), genes (500 ≤ n_genes ≤5,500), mitochondrial genes (percent_mito ≤1%), ribosomal genes (percent_ribo ≤1%), and soft max score detected by Solo (solo_softmax_scores ≤0.5). After read count normalization and log-transformation, highly variable genes were selected. Principal components were computed, and elbow plots were used to define the appropriate number of principal components for neighbor graph construction. Prior to manifold construction using UMAP, selected principal components were harmonized by using Python Harmonypy. Finally, nuclei were clustered using the network-based Leiden algorithm.

Statistical analysis

GraphPad Prism software 8.4.3 was used to perform statistical analysis. All data was depicted as mean±SEM either as scatterplots or bar graphs. Where possible, data sets were tested for

normal distribution and the appropriate statistical test was chosen accordingly. Either the unpaired or nested Student's t-test, a nested-, a classical one-way ANOVA or a two-way ANOVA (plus Bonferroni's post-test) was used to determine whether the difference between groups was statistically significant. A p-value lower than 0.05 was statistically significant.

References

Aravamudhan S, Türk C, Bock T, Keufgens L, Nolte H, Lang F, Krishnan RK, König T, Hammerschmidt P, Schindler N, *et al* (2021) Phosphoproteomics of the developing heart identifies PERM1 - An outer mitochondrial membrane protein. *J Mol Cell Cardiol* 154: 41–59

Bernstein NJ, Fong NL, Lam I, Roy MA, Hendrickson DG & Kelley DR (2020) Solo: Doublet Identification in Single-Cell RNA-Seq via Semi-Supervised Deep Learning. *Cell Syst* 11: 95-101.e5

Burkart AM, Tan K, Warren L, Iovino S, Hughes KJ, Kahn CR & Patti ME (2016) Insulin Resistance in Human iPS Cells Reduces Mitochondrial Size and Function. *Sci Rep* 6: 1–12

Giesbertz P, Ecker J, Haag A, Spanier B & Daniel H (2015) An LC-MS/MS method to quantify acylcarnitine species including isomeric and odd-numbered forms in plasma and tissues. *J Lipid Res* 56: 2029–2039

Lemoine MD, Krause T, Koivumäki JT, Prondzynski M, Schulze ML, Girdauskas E, Willems S, Hansen A, Eschenhagen T & Christ T (2018) Human Induced Pluripotent Stem Cell-Derived Engineered Heart Tissue as a Sensitive Test System for QT Prolongation and Arrhythmic Triggers. *Circ Arrhythmia Electrophysiol* 11: 1–15

Liao Y, Wang J, Jaehnig EJ, Shi Z & Zhang B (2019) WebGestalt 2019: gene set analysis toolkit with revamped UIs and APIs. *Nucleic Acids Res* 47: W199–W205

Litviňuková M, Talavera-López C, Maatz H, Reichart D, Worth CL, Lindberg EL, Kanda M, Polanski K, Heinig M, Lee M, *et al* (2020) Cells of the adult human heart. *Nature* 588: 466–472

Schwamb J, Feldhaus V, Baumann M, Patz M, Brodesser S, Brinker R, Claasen J, Pallasch CP, Hallek M, Wendtner CM, *et al* (2012) B-cell receptor triggers drug sensitivity of primary

CLL cells by controlling glucosylation of ceramides. *Blood* 120: 3978–3985

Skryabin B V., Gubar L, Seeger B, Kaiser H, Stegemann A, Roth J, Meuth SG, Pavenstädt H, Sherwood J, Pap T, *et al* (2020) Pervasive head-to-tail insertions of DNA templates mask desired CRISPR-Cas9-mediated genome editing events. *Sci Adv* 6

Ulmer BM, Stoehr A, Schulze ML, Patel S, Gucek M, Mannhardt I, Funcke S, Murphy E, Eschenhagen T & Hansen A (2018) Contractile Work Contributes to Maturation of Energy Metabolism in hiPSC-Derived Cardiomyocytes. *Stem Cell Reports* 10: 834–847
