# Supplementary material for: Human model of primary carnitine deficiency cardiomyopathy reveals ferroptosis as a novel mechanism
Source: Stem Cell Reports. 2023 Oct 5;18(11):2123–37. doi: 10.1016/j.stemcr.2023.09.002 (PMC10679537; doi:10.1016/j.stemcr.2023.09.002)
Supplement: Document S2. Article plus supplemental information [file mmc7.pdf]

Stem Cell Reports
Article
OPEN ACCESS

Human model of primary carnitine deficiency cardiomyopathy reveals ferroptosis as a novel mechanism

Malte Loos,[1,2,*] Birgit Klampe,[1] Thomas Schulze,[1] Xiaoke Yin,[3] Konstantinos Theofilatos,[3] Bärbel Maria Ulmer,[1,2] Carl Schulz,[1,2] Charlotta S. Behrens,[1,2] Tessa Diana van Bergen,[4] Eleonora Adami,[4] Henrike Maatz,[4] Michaela Schweizer,[5] Susanne Brodesser,[6] Boris V. Skryabin,[7] Timofey S. Rozhdestvensky,[7] Sara Bodbin,[8] Konstantina Stathopoulou,[1,2] Torsten Christ,[1,2] Chris Denning,[8] Norbert Hübner,[4,9,10] Manuel Mayr,[3] Friederike Cuello,[1,2] Thomas Eschenhagen,[1,2] and Arne Hansen[1,2,*]

[1]University Medical Center Hamburg-Eppendorf, Department of Experimental Pharmacology and Toxicology, 20246 Hamburg, Germany
[2]German Center for Heart Research (DZHK), Partner site Hamburg/Lübeck/Kiel, 20246 Hamburg, Germany
[3]King's British Heart Foundation Centre of Research Excellence, King's College London, London, UK
[4]Cardiovascular and Metabolic Sciences, Max Delbrück Center for Molecular Medicine in the Helmholtz Association (MDC), 13125 Berlin, Germany
[5]Electron Microscopy Unit, Center for Molecular Neurobiology Hamburg, University Medical Center Hamburg-Eppendorf, 20251 Hamburg, Germany
[6]Cluster of Excellence Cellular Stress Responses in Aging-associated Diseases (CECAD), Faculty of Medicine and University Hospital of Cologne, 50931 Cologne, Germany
[7]Transgenic animal and genetic engineering Models (TRAM), Faculty of Medicine of the Westfalian Wilhelms-University, 48149 Muenster, Germany
[8]Division of Cancer & Stem Cells, Biodiscovery Institute, University of Nottingham, NG7 2RD Nottingham, UK
[9]DZHK (German Centre for Cardiovascular Research), Partner Site Berlin, 13347 Berlin, Germany
[10]Charité-Universitätsmedizin, 10117 Berlin, Germany
*Correspondence: m.loos@uke.de (M.L.), ar.hansen@uke.de (A.H.)
https://doi.org/10.1016/j.stemcr.2023.09.002

SUMMARY

Primary carnitine deficiency (PCD) is an autosomal recessive monogenic disorder caused by mutations in *SLC22A5*. This gene encodes for OCTN2, which transports the essential metabolite carnitine into the cell. PCD patients suffer from muscular weakness and dilated cardiomyopathy. Two OCTN2-defective human induced pluripotent stem cell lines were generated, carrying a full OCTN2 knockout and a homozygous OCTN2 (N32S) loss-of-function mutation. OCTN2-defective genotypes showed lower force development and resting length in engineered heart tissue format compared with isogenic control. Force was sensitive to fatty acid-based media and associated with lipid accumulation, mitochondrial alteration, higher glucose uptake, and metabolic remodeling, replicating findings in animal models. The concordant results of OCTN2 (N32S) and -knockout emphasizes the relevance of OCTN2 for these findings. Importantly, genome-wide analysis and pharmacological inhibitor experiments identified ferroptosis, an iron- and lipid-dependent cell death pathway associated with fibroblast activation as a novel PCD cardiomyopathy disease mechanism.

INTRODUCTION

Primary carnitine deficiency (PCD) is an autosomal recessive disorder resulting in insufficient cellular carnitine (β-hydroxy-γ-trimethylammonium butyrate) uptake and low cytoplasmic concentrations (Longo et al., 2016). PCD is caused by pathogenic variants of *SLC22A5* (NC_000005.10), leading to loss of function of the encoded organic cation transporter novel family member 2 (OCTN2). OCTN2 is strongly expressed in the myocardium, skeletal muscle, fibroblasts, renal tubules, placenta, and intestine (Wagner et al., 2000). OCTN2 transports carnitine in a sodium-dependent manner and maintains intracellular concentrations 20- to 50-fold higher than extracellular (Tamai et al., 1998). Cytoplasmic carnitine palmitoyltransferase 1 (CPT1) catalyzes acylcarnitine formation. Carnitine-acylcarnitine translocase (CACT) transports acylcarnitine into the mitochondria, where carnitine palmitoyltransferase 2 (CPT2) reconverts acylcarnitine to acyl-CoA, which subsequently enters beta-oxidation. Low cytoplasmic carnitine concentration impairs fatty acid beta-oxidation and promotes cytoplasmic lipid accumulation. This results in a glucose-dependent energy metabolism, inhibited gluconeogenesis via pyruvate carboxylase inhibition, and diminished ketogenesis via lack of acetyl-CoA (Longo et al., 2016). Dependence on fatty acids renders cardiomyocytes particularly susceptible to PCD pathomechanisms. Carnitine conjugation is also important to reduce coenzyme A (CoA) bound to acyl residues. Cytoplasmic acyl-CoA accumulation results in ceramide, triglycerides, and cholesteryl-ester formation triggering inflammation and apoptosis (Cooper et al., 2015).

PCD symptoms are fasting-mediated hypoglycemia and hypoketonemia, liver and muscular dysfunction, and dilated cardiomyopathy (DCM) (Wang et al., 2014). PCD patients have low plasma carnitine concentrations (0–5 vs. 25–50 μmol/L) (Longo et al., 2006) because carnitine reabsorption in the renal proximal tubules system is impaired (Rasmussen et al., 2014). Patients receive a

This is an open access article under the CC BY license (http://creativecommons.org/licenses/by/4.0/).

lifetime oral high dose carnitine supplementation (100–200 mg/kg/day) (Wang et al., 2014). Side effects are vomiting, abdominal cramps, diarrhea, a fishy body odor, and accumulation of atherogenic trimethylamine N-oxide (Alesci et al., 2004; Koeth et al., 2013; Rebouche, 2004). Asymptomatic untreated PCD patients reach adulthood but have an increased risk for sudden cardiac death (Rasmussen et al., 2020). Detailed mechanisms of the PCD DCM remain poorly understood.

PCD is a rare disease with a prevalence of 1:20,000–1:70,000 (United States) (Magoulas and El-Hattab, 2012). The Faroe Islands, a Northern Atlantic archipelago, has a higher prevalence with 1:300 (Rasmussen et al., 2014). The *SLC22A5* c.95A>G (N32S) mutation is the characteristic PCD mutation on the Faroe Islands (Rasmussen et al., 2014). The overall approximate allelic frequency of *SLC22A5* pathogenic variants in the population is 0.5%–1% (Tan et al., 2020).

Juvenile visceral steatosis (JVS) mice represent an animal model of carnitine deficiency (Tomomura et al., 1992). This strain was discovered by coincidence to carry an OCTN2 p.L352R missense mutation (Koizumi et al., 1988). JVS mice exhibit high renal carnitine excretion and tissue lipid accumulation, hypoglycemia, hepatic steatosis, and growth retardation (Horiuchi et al., 1993). Also, JVS mice develop cardiac hypertrophy with accumulation of diacylglycerols and triglycerides (Saburi et al., 2003), lower myocardial ATP content (Asai et al., 2006), and high expression of the pyruvate dehydrogenase (PDH) inhibitor PDH kinase 4 (*PDK4*) (Horiuchi et al., 1999). A pharmacological carnitine deficiency animal model was established by administering the competitive OCTN2 and BBOX1 (γ butyrobetaine hydroxylase) inhibitor N-trimethyl-hydrazine-3-propionate to wild-type rats for 3 weeks. The rats developed increased renal carnitine excretion and hepatic steatosis, upregulation of carnitine shuttle proteins, but no apparent cardiac or skeletal phenotype (Spaniol et al., 2001; Degrace et al., 2004). No human induced pluripotent stem cell (hiPSC) model of PCD has been published so far.

The aim of this study was the development of a hiPSC model of PCD DCM. Two hiPSC lines were generated by CRISPR-Cas9 technology. The lines carry a full OCTN2-knockout (OCTN2 (−/−)) or the homozygous missense Faroe Islands founder mutation (OCTN2 (N32S), *SLC22A5* c.95A>G) (Rasmussen et al., 2014). Cardiomyocytes were differentiated, and the PCD disease phenotype was analyzed in genome-wide, molecular, functional, and morphological assays. Overall, this hiPSC PCD model replicates a wide range of PCD DCM characteristics and unveils ferroptosis linked to fibroblast activation as a novel disease mechanism of PCD.

RESULTS

CRISPR-Cas9

A control hiPSC line (OCTN2 (+/+)) was used for the CRISPR-Cas9 engineering and served as isogenic control. The CRISPR-Cas9 strategy is presented in Figures S1A and S1B. Representative Sanger sequencing traces of edited clones are depicted in Figure S1C and show the OCTN2 wild-type sequence (upper lane) with a heterozygous silent point mutation (c.277C>T) in the isogenic control. The middle lane shows introduction of the homozygous OCTN2 (N32S) c.95A>G point mutation in exon 1. Due to the large deletion of OCTN2 (−/−), Sanger sequencing could not be aligned (lower lane). PCR products with internal and flanking primers are shown in Figures S1D–S1F. The knockout was confirmed by quantitative reverse-transcription PCR (qPCR) of the *SLC22A5* transcript (Figure S1G). Southern blots validated the integrity of the edited locus. Figure S2A displays the predicted cutting sites for restriction endonucleases HindIII and EcoRI. Predicted fragment size and Southern blot results are shown in Figures S2B and S2C. NanoString analysis revealed normal karyotypes (Figure S2D).

Functional analysis

Cardiomyocytes were differentiated from all three hiPSC lines. Figure S3A shows no difference in the percentage of cardiac troponin T (cTnT)-positive cells (OCTN2 (+/+), 87.5% ± 2.5%, n = 10; OCTN2 (N32S), 84.1% ± 5.0%, n = 10; OCTN2 (−/−), 86.2% ± 8.2%, n = 9).

Spontaneously beating engineered heart tissues (EHTs) were subjected to video-optical force analysis starting on day 7 (Figures S3B–S3G). Contractile parameters reached a plateau after day 21. OCTN2 (−/−) showed lower force, higher contraction time, and shorter resting length (length of the EHT at relaxed state) for the entire culture time. Contractile values of day 21 were compared (Figures 1A–1F) and showed lower force and a shorter resting length for OCTN2 (−/−) compared with OCTN2 (+/+) (OCTN2 (+/+), 0.194 ± 0.004 mN, n = 153 EHTs; OCTN2 (N32S), 0.16 ± 0.01 mN, n = 108 EHTs; OCTN2 (−/−), 0.11 ± 0.01 mN, n = 91 EHTs). Both OCTN2-defective genotypes exhibited longer contraction time, and additionally OCTN2 (−/−) a longer relaxation time. No difference for the RR scatter, a surrogate for arrhythmic beating, could be detected. The effect of proarrhythmic triggers were not studied. Figures 1G and 1H and Videos S1, S2, and S3 depict representative average contraction peaks, images, and videos. Sharp microelectrode experiments revealed shorter APD_{90} of 211.0 ± 13.6 (SEM, n = 7) in OCTN2 (N32S) vs. 288.2 ± 15.5 (SEM, n = 9) in OCTN2 (+/+) (Figures 1I and 1J). Importantly, OCTN2 (+/+) showed no correlation between cardiomyocyte purity (% $cTNT^+$

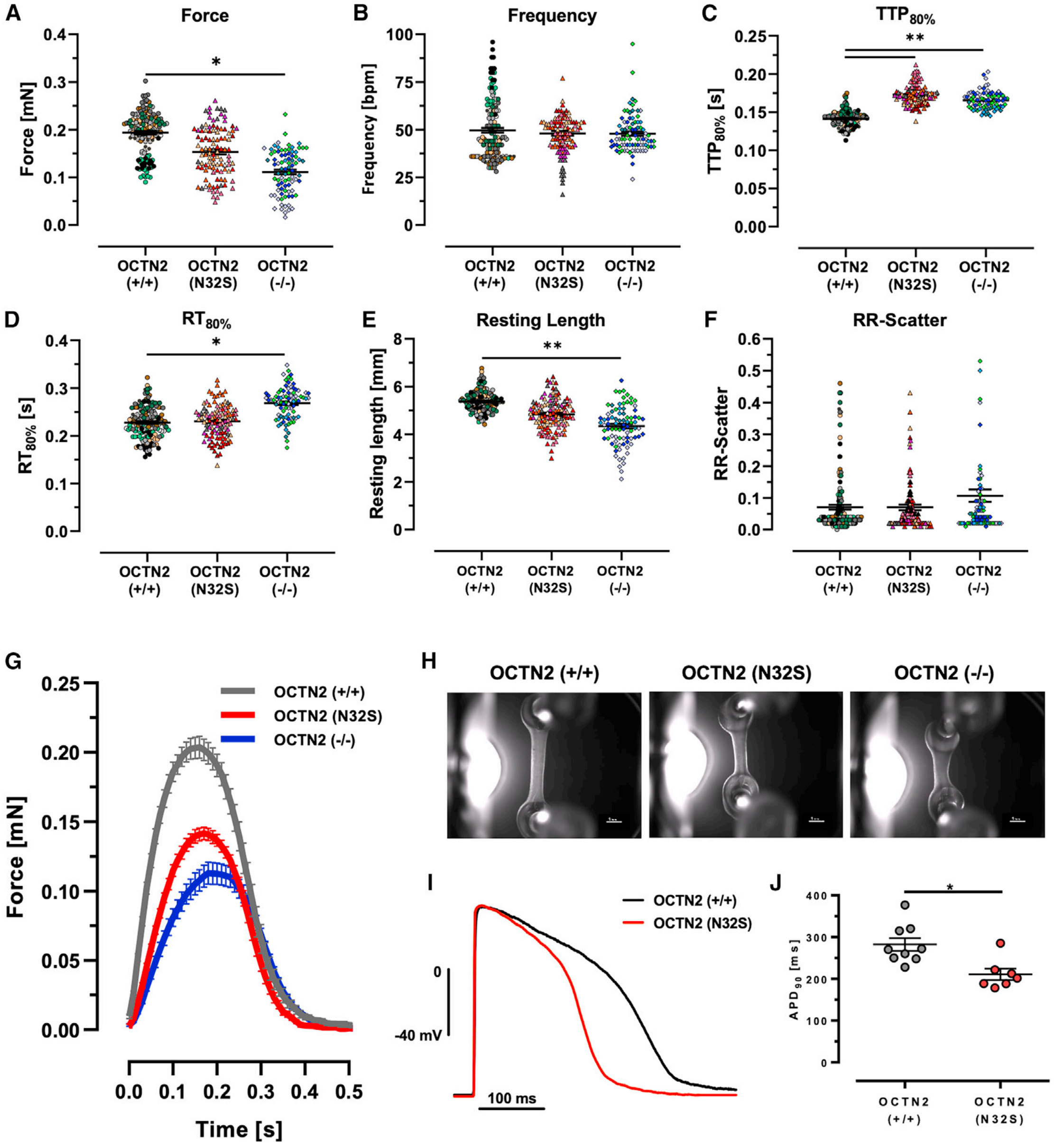

Figure 1. Contractile phenotype of OCTN2 genotypes

(A–H) Effect of OCTN2 genotype on contractile parameters of spontaneous beating EHTs on day 21. OCNT2 (+/+), n = 153 EHTs from 9 batches; OCTN2 (N32S), n = 108 EHTs from 7 batches; OCTN2 (−/−), n = 91 EHTs from 5 batches. Nested one-way ANOVA followed by Bonferroni's post test for multiple comparisons, *$p < 0.05$, **$p < 0.01$, ***$p < 0.001$, ****$p < 0.0001$. Each data point represents one EHT. Each color represents one independent differentiation batch. Data are expressed as mean ± SEM. (G) Representative average EHT contraction peaks of OCTN2 (+/+), OCTN2 (N32S), and OCTN2 (−/−). EHTs were electrically paced at 1.5 Hz in standard EHT medium, n = 9–14 EHTs from one batch. (H) Representative video-optical EHT images. Scale bars, 1 mm.

(I and J) (I) Action potential measurement: representative action potential for OCTN2 (+/+) and OCTN2 (N32S). (J) Action potential duration (APD90) of OCTN2 (+/+) and OCTN2 (N32S) by sharp microelectrode measurement at 1.5 Hz. Student's t test vs. OCTN2 (+/+), *$p < 0.05$. Data are expressed as mean ± SEM. Each data point represents one EHT. See also Figure S3.

input cells) and force as recently described (Mannhardt et al., 2020) and resting length in contrast to the OCTN2-defective genotypes (Figures 2A and 2B). This could suggest a relevant impact of non-cardiomyocytes on the phenotype.

In medium containing long-chain fatty acids (LCFA) plus carnitine [50 μM], force remained stable in OCTN2 (+/+) EHTs but declined in OCTN2-defective EHTs (Figure 2C), indicating a reduced ability to metabolize LCFA. In glucose

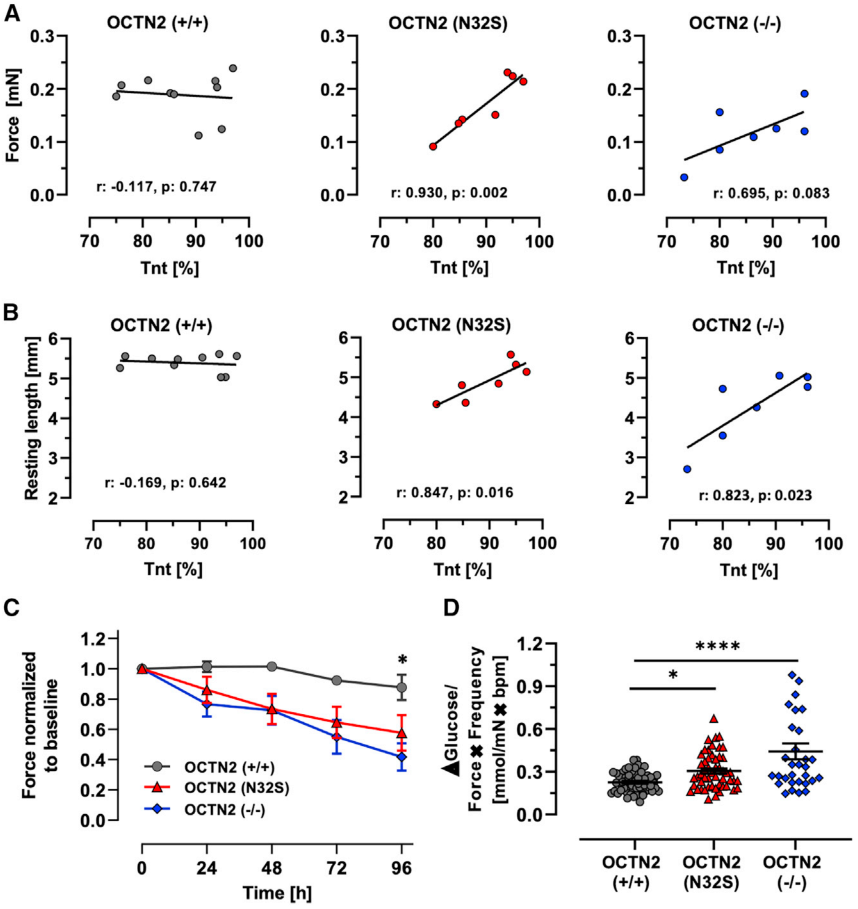

Figure 2. Effect of input cell compostion and cell culture media on EHT properties

(A and B) Pearson correlation of (A) force and (B) resting length of EHTs with percentage of cTNT-positive input cells for EHT generation. OCTN2 (+/+), n = 10; OCTN2 (N32S), n = 7; OCTN2 (−/−), n = 7 differentiation batches. Each replicate represents the mean value of 7–20 EHTs for the specific differentiation batch.

(C) EHT force development in fatty acid medium. Serum-free cell culture medium was supplemented with 50 μM carnitine, linoleic acid- and oleic acid-albumin. Data are normalized to baseline force. OCNT2 (+/+), n = 11 EHTs from 2 batches; OCTN2 (N32S), n = 11 EHTs from 2 batches; OCTN2 (−/−), n = 12 EHTs from 2 batches. Two-way ANOVA vs. OCNT2 (+/+) followed by Bonferroni's post test for multiple comparisons, *p < 0.05. Data are expressed as mean ± SEM.

(D) Difference in ΔGlucose medium concentration divided by product of individual spontaneous beating frequency × force. ΔGlucose = glucose concentration at baseline minus glucose concentration after 24 h of incubation in medium containing 5.5 mM glucose and 10% horse serum. OCNT2 (+/+), n = 59 EHTs from 5 batches; OCTN2 (N32S), n = 51 EHTs from 4 batches; OCTN2 (−/−), n = 28 EHTs from 4 batches. One-way ANOVA followed by Bonferroni's post test for multiple comparisons, *p < 0.05, **p < 0.01, ***p < 0.001, ****p < 0.0001. One data point represents one EHT. Data are expressed as mean ± SEM. See also Figure S3.

and serum-containing EHT medium, delta-glucose and lactate values were higher for OCTN2 (N32S), but not OCTN2 (−/−) compared with OCTN2 (+/+). The delta-lactate/delta-glucose ratio as a surrogate for anaerobic glucose metabolism showed no difference (Figures S3H–S3J). Higher glucose consumption for OCTN2 (N32S) and OCTN2 (−/−) became evident when normalized to workload (force × beating frequency) (OCTN2 (+/+), 0.23 ± 0.01 mM/bpm × mN; OCTN2 (N32S), 0.3 ± 0.1 mM; OCTN2 (−/−), 0.4 ± 0.1 mM) (Figure 2D).

Proteomics, Seahorse

A total of 3,425 proteins was detected by tandem mass tag-based proteomic analysis, of which 1,772 proteins differed significantly between OCTN2 (+/+) and OCTN2 (N32S) and 2,050 differed significantly between OCTN2 (+/+) and OCTN2 (−/−), respectively (p < 0.05). A detailed summary of detected proteins is shown in Table S1, sheet 1. Due to their low abundance, OCTN2 or low-affinity transporters (SLC22A16, SLC16A9, and SLC6A14) were not detected. Principal-component analysis revealed separate clustering of OCTN2 (+/+) from OCTN2 (N32S) and OCTN2 (−/−) (Figure 3A). Volcano plot depiction highlights higher abundance of fibrosis/extracellular matrix proteins such as caldesmon1, collagen type I alpha 1 chain, transgelin 2, fibronectin 1, and vitronectin in OCTN2 (N32S) EHTs (Figure 3B). Moreover, ceramide transfer protein (CERT) was among the 10 most abundant proteins in OCTN2 (N32S). In contrast, the fatty acid transporters cluster of differentiation 36, fatty acid-binding protein 5, and cardiomyogenesis transcriptional regulator GATA binding protein 4 were among the top 10 lower abundant proteins in OCTN2 (N32S). Detailed grouping of proteins related to participation in pathways revealed concordant expression patterns for OCTN2 (N32S) and OCTN2 (−/−) vs. OCTN2 (+/+) (Figures 3C and S4C). KEGG pathway analysis revealed enrichment of proteins related to ribosome, ferroptosis, extracellular matrix, and N-glycan and O-glycan

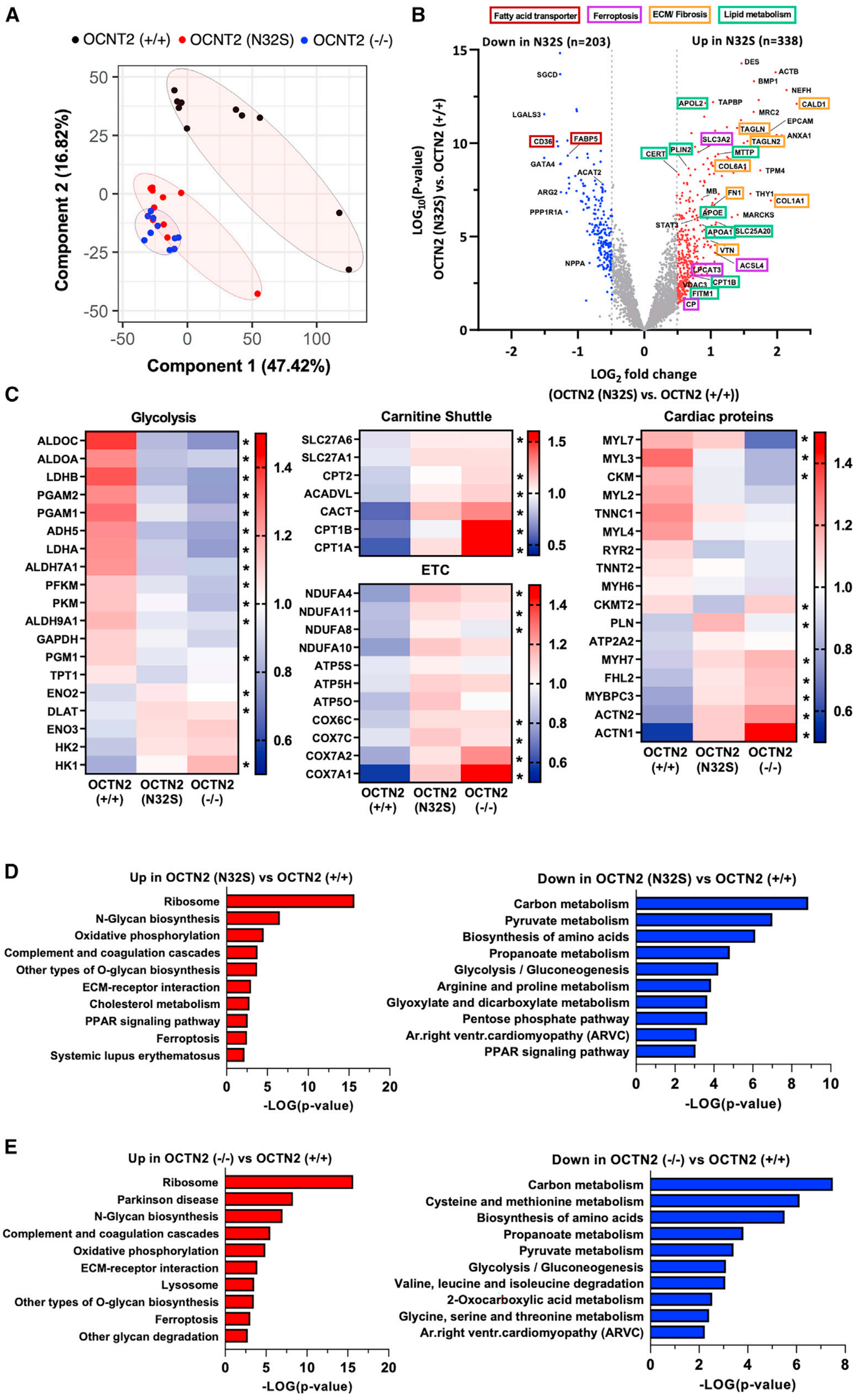

(legend on next page)

biosynthesis in OCTN2 (N32S) and OCTN2 (−/−). Conversely, enrichment analysis of lower abundant proteins highlighted the KEGG pyruvate- and propanoate metabolism, glycolysis, and pentose phosphate pathways. Furthermore, anti-ferroptotic cysteine metabolism was underrepresented in the OCTN2 (−/−) (Figures 3D and 3E). A detailed summary of proteins in the enriched pathways is depicted in Table S1, sheets 2 and 3. Mitochondrial DNA was lower in OCTN2 (N32S) (Figure S4A). Both OCTN2-defective lines showed lower oxygen consumption rate at baseline, as well as in response to oligomycin, carbonyl cyanide-p-trifluoromethoxyphenylhydrazone, and rotenone exposure in Seahorse experiments in glucose-based medium, suggesting lower glucose oxidation (Figure S4B).

Carnitine supplementation: Acylcarnitine and ceramide content, force, lipid mass spectrometry, TEM

Supraphysiological carnitine concentration (2 mM, replicating plasma concentrations during treatment) led to a reduction of glucose consumption and lactate production for all genotypes (Figures S5A–S5C). Notably, glucose consumption normalized to cardiac workload was reduced only for the OCTN2-defective cell lines (Figure 4A). This was associated with an increase in force and relaxation time for all lines (Figures 4B, 4C, and S5D–S5G). Transcript levels of PDK4, the inhibitor of PDH and important metabolic regulator, were higher in OCTN2 (N32S) and OCNT2 (−/−) and attenuated to the level of isogenic control by carnitine supplementation (Figures S6H and S6I). Liquid chromatography-mass spectrometry revealed 5-fold lower content for C16:1, C18:0, C18:1, and C18:2 acylcarnitines in OCTN2 (N32S). Carnitine supplementation resulted in a higher content of C16:0, C16:1, C18:1, and C18:2 acylcarnitines for OCTN2 (+/+) and C18:1 and C18:2 acylcarnitines for OCTN2 (N32S). Quantification of ceramide content, a metabolite of accumulated acyl-CoA (Cer16:0, Cer18:0, Cer22:0, Cer24:0, Cer24:1) revealed neither an effect of the genotype nor carnitine supplementation (Figures 4D and 4E).

Transmission electron microscopy (TEM) (Figures 5A–5F) showed elongated myofilaments and structured mitochondria in OCTN2 (+/+) EHTs. OCTN2 (N32S) displayed lower abundance of mitochondria and moreover with structural defects, and high frequency of large lipid droplets in close association with mitochondria and sarcomeres. OCTN2 (−/−) also exhibited mitochondria with disturbed structures and increased membrane density but less aggregation of lipid droplets. Carnitine supplementation increased mitochondria for all genotypes and reduced the occurrence of lipid droplets. Further high-power TEM analysis revealed electron dense matrix (Dixon et al., 2012) and loss of cristae (Yagoda et al., 2007) and rupture of the outer mitochondrial membrane (Friedmann Angeli et al., 2014) but preserved nuclear membrane morphology (N) (Figure S4D–S4O) in the OCNT2-defective lines indicating hallmarks of ferroptosis.

Single nuclear RNA sequencing

A pool of four EHTs per genotype was subjected to single nuclear RNA sequencing (snRNA-seq). OCTN2 (+/+), (N32S), and (−/−) samples were sequenced with average sequencing depth of 39,324, 28,771, and 26,374 reads per nucleus. Following quality control filtering, snRNA-seq data were pooled to a total number of 11,225 nuclei (OCTN2 (+/+) = 3,135, OCTN2 (N32S) = 3,761, OCTN2 (−/−) = 4,329 cells). Uniform Manifold Approximation and Projection (UMAP) and Leiden clustering revealed 5 main cell clusters. Marker genes distinguished: cardiomyocytes, proliferating cardiomyocytes, fibroblasts, and endothelial and myeloid cells (Figure 6A). In OCTN2 (+/+), cardiomyocytes represented 94% of all cells with 14% of these cells showing markers of proliferation (Figures 6B and 6C). Subclustering of cardiomyocytes revealed 10 subclusters (Figure S6). Subcluster CM4 was dominant in OCTN2 (+/+), while CM1 and 2 were more prominent in OCTN2-defective lines. Interestingly, KEGG analysis revealed enrichment of the GPR40 pathway in CM4, describing free fatty acid receptor 1 signaling (Figure S6C).

Figure 3. Tandem mass tag-based quantitative proteomic analysis of EHTs

(A) Principal-component analysis (PCA) of OCNT2 (+/+) (black, n = 10) OCTN2 (N32S) (red, n = 10), and OCTN2 (−/−) (blue, n = 10) EHTs based on their proteomic profiles. Each dot represents one EHT.

(B) Volcano plot of log2 fold changes of OCTN2 (N32S) vs. OCTN2 (+/+) and log10 of the p values with color-coded significance levels ($p > 0.05$) and fold change >1.4.

(C) Clustering analysis of proteins related to metabolic pathways and the myocardium. Heatmaps display the relative abundance of proteins involved in glycolysis, carnitine shuttle, electron transport chain (ETC), and the myocardium. OCNT2 (+/+), mean of 10 EHTs from 1 batch; OCTN2 (N32S), mean of 10 EHTs from 1 batch; OCTN2 (−/−), mean of 10 EHTs from 1 batch. Protein levels are depicted as a color code ranging from blue (low abundance) to red (high abundance). Kruskal-Wallis test, * indicates statistically significant difference of OCTN2 (+/+) against OCTN2 (N32S) or OCTN2 (−/−).

(D and E) Pathway enrichment analysis of proteins identified by proteomic analysis. Depicted are KEGG pathways of significantly enriched proteins that were significantly higher (red) or lower (blue) abundant in (D) OCTN2 (N32S) vs. OCTN2 (+/+) and (E) OCTN2 (−/−) vs. OCTN2 (+/+), $p < 0.05$, fold change >1.4. See also Figure S4.

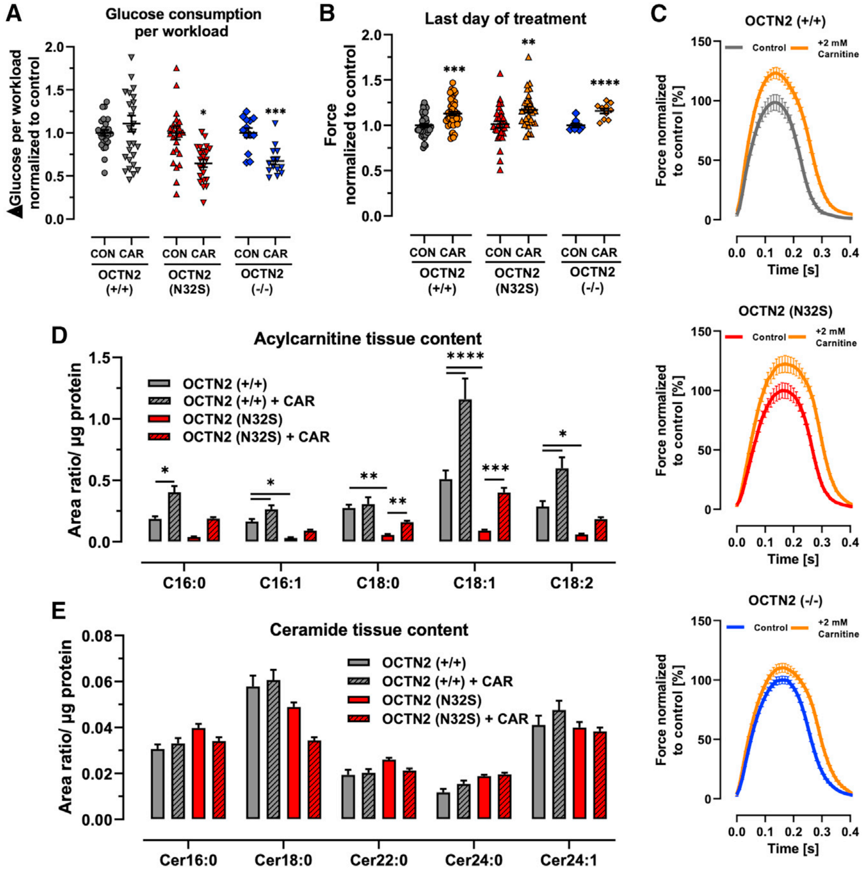

Figure 4. Effect of carnitine medium supplementation on EHT properties

(A) Effect of carnitine supplementation ΔGlucose per workload (ΔGlucose = Glucose concentration at baseline minus glucose concentration after 24 h of incubation; workload = force × frequency). Nested t test vs. CON, $^{**}p < 0.01$, $^{***}p < 0.001$, $^{****}p < 0.0001$. OCNT2 (+/+) control, n = 27 EHTs from 3 batches; OCNT2 (+/+) + carnitine (2 mM), n = 28 EHTs from 3 batches; OCTN2 (N32S) control, n = 23 EHTs from 3 batches; OCTN2 (N32S) + carnitine (2 mM), n = 23 EHTs from 3 batches; OCTN2 (−/−) control, n = 13 EHTs from 3 batches; OCTN2 (−/−) + carnitine (2 mM), n = 16 EHTs from 3 batches. Data are expressed as mean ± SEM.

(B) Effect of carnitine supplementation on force of spontaneous beating EHTs at the last day of treatment (days 33-42). Values were normalized to last day of treatment of untreated control. Student's t test vs. CON, $^{**}p < 0.01$, $^{***}p < 0.001$, $^{****}p < 0.001$. OCNT2 (+/+) control, n = 54 EHTs from 4 batches; OCNT2 (+/+) + carnitine (2 mM), n = 49 EHTs from 4 batches; OCTN2 (N32S) control, n = 36 EHTs from 3 batches; OCTN2 (N32S) + carnitine (2 mM), n = 33 EHTs from 3 batches; OCTN2 (−/−) control, n = 9 EHTs from 1 batch; OCTN2 (−/−) + carnitine (2 mM), n = 9 EHTs from 1 batch. Data are expressed as mean ± SEM.

(C) Effect of carnitine supplementation on average contraction peaks. Depicted are representative average EHT contraction peaks of OCTN2 (+/+), OCTN2 (N32S), and OCTN2 (−/−). EHTs were electrically paced at 1.5 Hz in standard EHT medium ± carnitine (2 mM). Values were normalized to untreated control. n = 9–16 EHTs per condition from 1 batch.

(D and E) Liquid chromatography-mass spectrometry analysis of acylcarnitines and ceramides. Effect of carnitine supplementation on (D) acylcarnitine and (E) ceramide content of OCNT2 (+/+) and OCTN2 (N32S) EHTs after 33 days of culture and supplementation. Two-way ANOVA followed by Bonferroni's post test for multiple comparisons, $^{*}p < 0.05$, $^{**}p < 0.01$, $^{***}p < 0.001$, $^{****}p < 0.0001$. Data are expressed as mean ± SEM. n = 4 EHT pools (containing 3 EHTs each) per genotype and carnitine supplementation from 1 batch. See also Figure S5.

A lower representation of CM4 in the OCTN2-defective lines is compatible with the lower abundance of fatty acid transporters in the proteomics analysis. In OCTN2-defective lines, cardiomyocytes represented a smaller fraction (OCTN2 (N32S), 85%; OCTN2 (−/−), 67%). Reversely, these lines showed a higher fraction of fibroblasts (OCTN2 (+/+), 4%; OCTN2 (N32S), 10%; OCTN2 (−/−), 23%) (Figures 6B and 6C). Subclustering of fibroblasts identified four states. Fibroblasts states with markers of TGF-β signaling, proliferation, and secretion (FB1, FB3, and FB4), were more prominent in OCTN2-defective lines (Figures 6D, 6E, 6F, and S7A). Genotype-specific analysis of significant KEGG pathway enrichment in all fibroblast subcluster revealed relaxin-, ECM-, and focal adhesion-related pathways (Figure S7B).

Endothelial and myeloid cells were almost absent in OCTN2 (+/+) and represented 5% and 1% in OCTN2 (N32S) and 8% and 1% in OCTN2 (−/−), respectively (Figure 6B). Endothelial cells expressed PDE3A, CASC15, and MECOM, and myeloid cells expressed PTPRC (CD45), CD163, and AOAH (Figure 6C). KEGG pathway analysis was not possible because significant pathways could only be detected for OCTN2 (−/−) endothelial cells (Figure S7B).

Ferroptosis, fibroblast activation

Proteomics analysis revealed enrichment of the KEGG pathway ferroptosis, an iron-dependent lipid peroxidation-mediated cell death mechanism. Extraction of an extended list of pro- and anti-ferroptotic proteins (Chen et al., 2021) from the proteomics data identified a

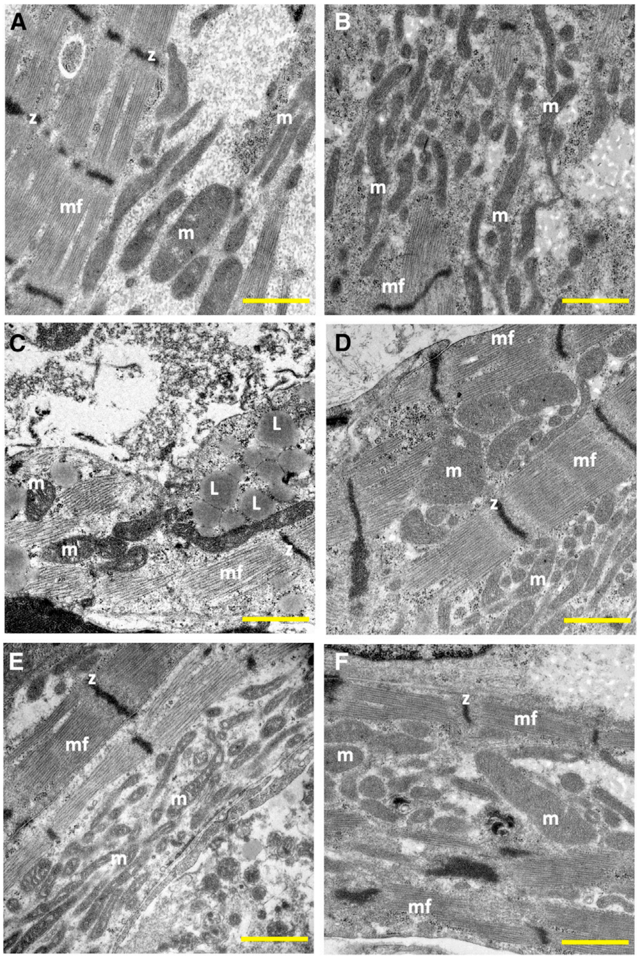

Figure 5. Transmission electron microscopy of OCTN2 EHTs
(A and B) OCTN2 (+/+), (C and D) OCTN2 (N32S), and (E and F) OCTN2 (−/−). (A, C, and E) Untreated. (B, D, and F) Supplemented with carnitine (2 mM). mf, myofilaments; z, z-line; m, mitochondria; L, lipid droplet. Scale bars, 1 μm. See also Figure S4.

surprisingly uniform regulation with higher abundance of pro- and lower abundance of anti-ferroptotic proteins in the OCTN2-defective genotypes (Figure 7A). Noteworthy among these were also the key regulators ACSL4 (Doll et al., 2017) and LPCAT3, which synergistically drive the accumulation of iron-dependent toxic lipid peroxides (Li and Li, 2020). On the other hand, pro- and anti-ferroptosis transcripts did not show a differential expression in snRNA-seq, suggesting post-transcriptional regulation (Figure 7B). Evidence for both ferroptosis and fibroblast activation in this study and previous reports that demonstrate a mechanistic link (Fang et al., 2019; Gong et al., 2019) provided the rationale to analyze the effect of the potent ferroptosis inhibitor liproxstatin on fibrosis markers. OCTN2 (N32S) EHTs revealed higher transcript levels of *ACTA2*, *COL1A1*, *POSTN*, *TGFB*, *FN*, and *CCN2* than OCTN2 (+/+). Liproxstatin induced a significant attenuation of fibrosis transcript levels in OCTN2 (N32S), which was associated with a moderate increase in force (Figures 7C and 7D).

DISCUSSION

This study describes the first hiPSC PCD DCM *in vitro* model. The main results are (1) successful genetic engineering of two hiPSC lines, a homozygous OCTN2 (N32S) mimicking the patient situation and an OCTN2 (–/–) knockout, (2) replication of the PCD DCM phenotype involving low acylcarnitine tissue content and force, complex metabolic remodeling, and ultrastructural alteration, (3) corroboration of the of OCTN2 (N32S) phenotype by high-level concordance with OCTN2 (−/−) across various assays, (4) discovery of ferroptosis linked to fibroblast activation as a novel PCD DCM mechanism.

The approach to engineer the human-relevant OCTN2 (N32S) loss of function point mutation in parallel with a complete OCTN2 knockout turned out to be insightful since concordant changes of several parameters (e.g., contractile parameters, proteomics profile, snRNA-seq clustering of (non)-cardiomyocyte subpopulations) were observed in both lines, thus validating lack of functional OCTN2 as the main pathomechanism governing disease origin.

Typical features of PCD DCM in both patients and animal models are reduced tissue contents of carnitine derivates, functional impairment of glucose and lipid metabolism, myocardial steatosis, severe hyperglycemia (Horiuchi et al., 1993; Asai et al., 2006), and short QT syndrome (Roussel et al., 2016). Several key features could be successfully replicated in this hiPSC-CM model such as lower force and higher sensitivity to fatty acid-based media, reduced acylcarnitine tissue content intracellular lipid droplet accumulation, and shorter action potential duration in OCTN2-defective EHT. Genome-wide analysis revealed complex metabolic remodeling and mitochondrial dysfunction compatible with (acyl)-carnitine deprivation (Asai et al., 2006). Noteworthy, dysregulation of several metabolic pathways (e.g., pyruvate metabolism, glycolysis, and the pentose phosphate pathway) were not as expected in a carnitine-deficient and fatty acid oxidation-impaired model. Likewise, the higher abundance of proteins related to carnitine metabolism (e.g., CPT1, CPT2, and CACT (SLC25A20)) was unexpected. This apparent discrepancy is related to the integration of primary changes and also compensatory metabolic adaptation in the proteomic analysis. Hence, the upregulation of carnitine shuttle proteins likely represents a compensatory effect as also described in secondary carnitine deficiency animal models (Liepinsh et al., 2008). The lower and higher abundance of proteins

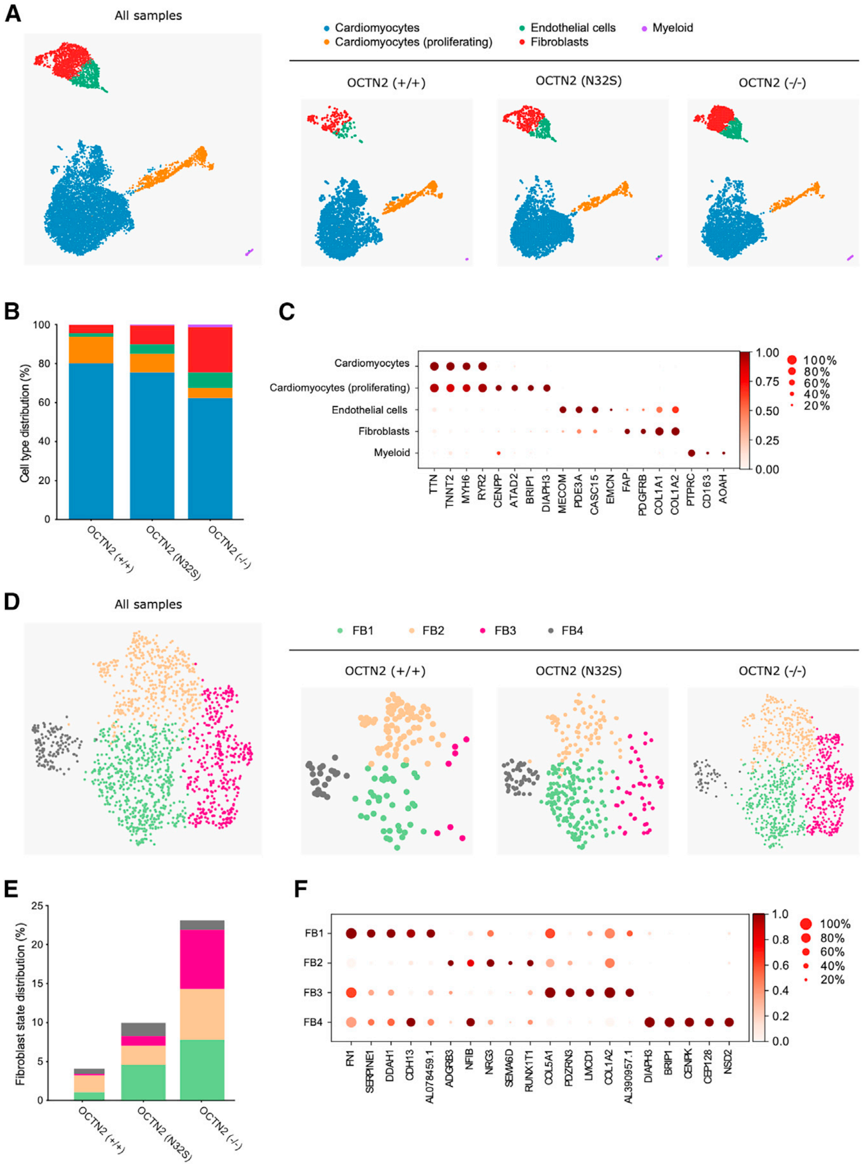

Figure 6. Cellular heterogeneity in OCTN2 genotypes in EHTs

(A) Representative UMAP plot after snRNA-seq of all samples and individual genotypes, n = 1 EHT pool (4 EHTs) per genotype; OCTN2 (+/+) (3,674 cells), OCTN2 (N32S) (4,525 cells), OCTN2 (−/−) (5,108 cells). Five distinct cell clusters were identified: cardiomyocytes, cardiomyocytes (proliferating), endothelial cells, fibroblasts, and myeloid cells.

(B) Percentage of cell types per genotype.

(C) Dot plot graph showing the relative expression of cell-specific marker genes. Expression levels are depicted as a color code ranging from light red (low expression) to dark red (high expression) as mean of log2 fold of expression. The dot size indicates the percentage of cells expressing the gene.

(D) Representative fibroblast subcluster FB1-4 UMAP plot of all samples and individual genotypes.

(E) Percentage of fibroblast states per genotype. For each genotype, the total percentage of fibroblast states equals the percentage of fibroblast abundance identified in (B).

(F) Dot plot graph showing the relative expression of fibroblast-specific marker genes in fibroblast states. Scaled expression levels are depicted as a color code ranging from light red (low expression) to dark red (high expression) as mean of log2 fold of expression. The dot size indicates the percentage of cells expressing the gene. See also Figures S6 and S7.

related to glucose metabolism and oxidative phosphorylation indicates dysregulation of pathways related to glucose and lipid metabolism. In the case of glucose, the Seahorse experiments and high PDK4 expression levels indicate a deficit in glucose oxidation, which is line with the established carnitine-mediated activation of glucose oxidation (Broderick et al., 1995).

Low cytoplasmic carnitine concentration leads to diminished acylcarnitine formation and subsequent beta-oxidation (Longo et al., 2006). Indeed, mass spectrometry analysis revealed lower tissue content of several long-chain acylcarnitines in OCTN2 (N32S) EHTs. In addition, declining force in LCFA media suggested a defect in LCFA metabolism. Surprisingly, mass spectrometry did not reveal a difference in ceramide content. However, ceramides do not represent the final product, but rather a metabolic intermediate that can be processed to sphingolipid derivates such as glucosylceramides and sphingomyelin (MacEyka and Spiegel, 2014). For this conversion, ceramides are transported from the endoplasmic reticulum into the trans-Golgi apparatus by ceramide transporter CERT (Bandet and Hajduch, 2021). Remarkably, CERT was among the 10 most significantly higher abundant proteins in OCTN2 (N32S) EHTs, suggesting that it belongs to the compensatory mechanisms and prevents the accumulation of toxic ceramides.

PCD carnitine supplementation therapy results in plasma concentrations in the millimolar range, enabling

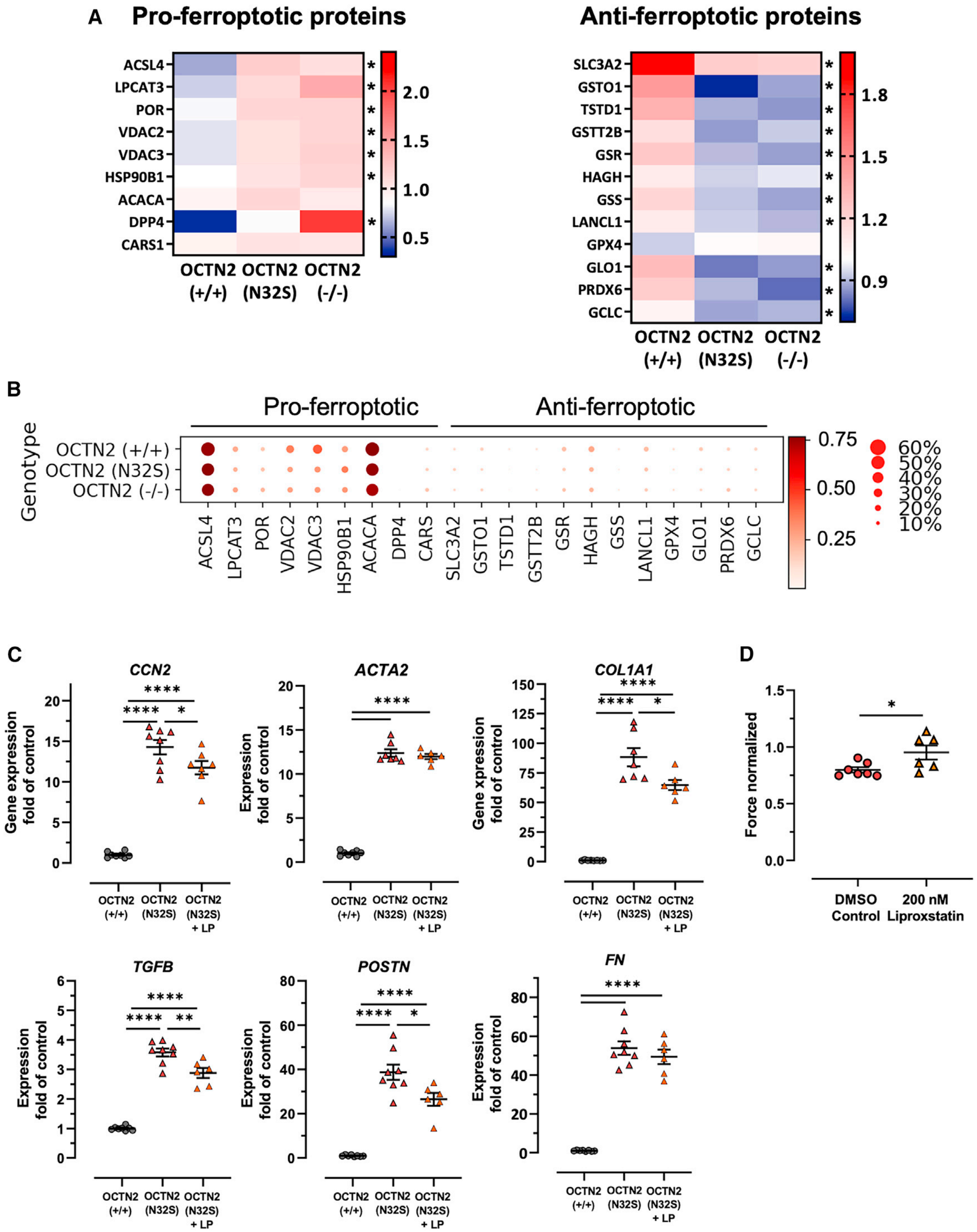

Figure 7. Evidence for ferroptosis pathway activation in tandem mass tag-based quantitative proteomic analysis and pharmacological inhibitor experiments

(A) Proteomic analysis heatmaps display the relative abundance of pro- and anti-ferroptotic proteins of all genotypes. OCNT2 (+/+), mean of 10 EHTs from 1 batch; OCTN2 (N32S), mean of 10 EHTs from 1 batch; OCTN2 (−/−), mean of 10 EHTs from 1 batch. Protein levels are depicted as a color code ranging from blue (low abundance) to red (high abundance).

(B) snRNA-seq dot plot graph showing the scaled relative expression of pro- and anti-ferroptotic markers across all genotypes for all cells. The dot size indicates the percentage of cells expressing the respective gene.

(legend continued on next page)

intracellular transport via the low-affinity transporter $ATB^{0,+}$ (SLC6A14) (Longo et al., 2016). Carnitine supplementation (2 mM) in this study had a strong effect on metabolic aspects such as acylcarnitine tissue content, glucose consumption per cardiac work, PDK4 transcript level, and lipid droplet accumulation. Nevertheless, the force restoration was minor and carnitine-mediated increase in force was similar for all three genotypes, suggesting a non-specific effect potentially related to the induction of sodium current and subsequent inhibition of Na^+/K^+-ATPase as described previously (Wu and Corr, 1994). The discrepancy between strong metabolic and small force effects of carnitine supplementation suggests the relevance of additional mechanisms. The shorter EHT resting length, the positive correlation between cardiomyocyte purity with force and resting length, and the enrichment of the KEGG pathway extracellular matrix in the OCTN2-defective genotypes point to the relevance of fibroblast activation. In support of this, snRNA-seq revealed a more prominent fibroblast cluster in the OCTN2-defective gen-otypes, which expressed markers indicative of activated and secretory fibroblast state. Of note, markers of activated fibroblasts in this study (*POSTN*, *FN1*, *FAP*, and *NOX4*) overlap substantially with the fibroblast signatures in two failing heart snRNA-seq DCM studies (Chaffin et al., 2022; Nicin et al., 2021). Interestingly, the central role of fibroblasts in this hiPSC-CM PCD model is paralleled by clinical findings of overt myocardial fibrosis in PCD patients (Wang et al., 2014; Tomlinson et al., 2018). This study does not allow conclusions to be drawn as to whether fibroblast activation is an autonomic or a paracrine effect via cardiomyocytes. In any case, fibroblast activation is not specific to ferroptosis in cardiomyopathies since it was also described in non-cardiac conditions (Zhang et al., 2021; Gong et al., 2019).

Ferroptosis is well compatible with PCD DCM, since it is driven by the accumulation of polyunsaturated fatty acids (PUFAs) linked to CoA in cell membranes (Yang et al., 2016). PUFA accumulation is relevant for PCD as cytosolic carnitine deficiency impairs PUFA metabolization to acylcarnitine. ACSL4 catalyzes the esterification of long-chain PUFA to acyl-CoA, represents a central pro-ferroptotic regulator (Doll et al., 2017), and showed higher expression and protein abundance in this model. The abundance of the central anti-ferroptotic enzyme GPX4 was not lower in OCTN2-defective lines. Notably, glutathione is an important cofactor for GPX4 activity and proteins involved in glutathione metabolism such as glutamate-cysteine ligase, glutathione synthetase, and glutathione-disulfide reductase were of lower abundance in OCTN2-defective lines.

Taken together, the development of a human PCD *in vitro* model and the discovery of ferroptosis linked to fibroblast activation reveals novel insight into PCD-associated metabolic cardiomyopathy and is paving the way for the development of more specific treatment strategies.

EXPERIMENTAL PROCEDURES

A detailed description of experimental procedures is available online.

Resource availability

Corresponding author

Arne Hansen, University Medical Center Hamburg-Eppendorf, Department of Experimental Pharmacology and Toxicology, 20246 Hamburg, Germany. E-mail: ar.hansen@uke.de

Materials availability

This study did not create any unique reagents. The genetically engineered OCTN2-deficient hiPSC lines are available with a completed material transfer agreement.

Data and code availability

The mass spectrometry proteomics data have been deposited to the ProteomeXchange Consortium via the PRIDE [1] partner repository with the dataset identifier PXD036026. The snRNA-seq datasets are available in the Gene Expression Omnibus repository under accession no. GSE211650 (https://www.ncbi.nlm.nih.gov/geo/query/acc.cgi?acc=GSE211650). The acylcarnitine and ceramide mass spectrometry data are available under https://uni-koeln.sciebo.de/s/4ebUQhDuFH1UPoA.

hiPSC cell culture conditions

An established hiPSC control cell line (hiPSCreg code UKEi001-A) derived from a healthy individual served as the starting point for the genetic engineering approach and as the isogenic control for the engineered hiPSC lines. This hiPSC line was generated by reprogramming dermal fibroblasts from a skin biopsy using the CytoTune (Life Technologies) 2.0 Sendai Reprogramming Kit under feeder-free conditions. All basic stem cell culture work was performed as recently described (Shibamiya et al., 2020). In brief, hiPSC culture

(C) Effect of the ferroptosis inhibitor liproxstatin: qPCR analysis gene expression of genes related to fibroblast activation. Gene expression was normalized to GUSB over OCTN2 (+/+) control. OCNT2 (+/+), n = 8 EHTs from 1 batch; OCTN2 (N32S), n = 8 EHTs from 2 batches; OCTN2 (−/−), n = 6–8 EHTs from 1 batch. One-way ANOVA followed by Bonferroni's post test for multiple comparisons, *$p < 0.05$, **$p < 0.01$, ****$p < 0.0001$. Data are expressed as mean ± SEM.
(D) Effect of liproxstatin (200 nM) on contractile force in OCTN2 (N32S) EHTs. Data are expressed as mean ± SEM, *$p < 0.05$, unpaired t test. See also Figure S7.

was based on the expansion of a master cell bank at passage 25–35 on Geltrex-coated cell culture flasks in FTDA medium (Table S2) under hypoxic conditions (5% O_2). Standard passaging was performed twice a week (3–4 days passaging interval) with Accutase solution (Sigma-Aldrich) with a plating density of 4.5–7.0 × 10^4 hiPSC/cm^2. Maximal expansion was for 40 passages with regular screening for mycoplasma contamination by PCR amplification. SSEA3 surface marker served as a pluripotency marker and was analyzed by flow cytometry. All procedures involving the generation and analysis of hiPSC lines were approved by the local ethics committee in Hamburg (Az PV4798, 28.10.2014).

CRISPR-Cas9-mediated gene editing

OCTN2 (N32S) missense mutation

The *SLC22A5* gene locus was Sanger sequenced in the hiPSC OCTN2 (+/+) control line. CRISPR-Cas9 technology was used to engineer the c.95A>G (N32S) mutation into the *SLC22A5* wild type. IDT Custom Alt-R CRISPR-Cas9 gRNA software and CRISPOR (Concordet and Haeussler, 2018) were used to identify potential gRNA binding sites at the gene locus. The OCTN2 NCBI Reference (NG_008982.2) was provided as a target sequence. Targets for gRNAs were chosen based on the lowest cut-to-mutation distance under consideration of a high on-target potential and low off-target risk. A single-stranded oligodeoxynucleotide served as an exogenous donor template, containing the OCTN2 c.95A>G, p.N32S mutation. In addition, a silent mutation was introduced in the PAM sequence to prevent CRISPR-Cas9 re-cutting after successful genomic integration of the template by HDR. Edited clones were identified by PCR amplification and subsequent Sanger sequencing. A schematic overview of the HDR strategy is depicted in Figure S1A. SsODN and gRNA sequences are shown in Table S3, sheet 1.

OCTN2 (−/−) knockout

A combinatorial CRISPR strategy was used to engineer a knockout of the *SLC22A5* gene in the isogenic control hiPSC OCTN2 (+/+). Two gRNAs were designed to introduce a deletion of 17.3 kb spanning from the promotor region to exon 5 (NG_008982.2). For deletion validation, primer pairs were designed to amplify products inside the deletion region and the gRNA target sites. Also, primers flanking the two cutting sites were designed. Edited clones were identified by PCR amplification and subsequent Sanger sequencing. A schematic overview of the knockout strategy is displayed in Figure S1B. To distinguish between unedited, heterozygous, and homozygous edited clones, the PCR products were separated by agarose gel electrophoresis (1%, w/v), followed by Midori green staining. The target gRNA sequences are shown in Table S3, sheet 1.

Cardiac differentiation

hiPSCs were differentiated into cardiomyocytes with an embryoid body (EB)- and growth factor-based three-stage protocol which was recently described (Breckwoldt et al., 2017). In brief, hiPSCs were expanded on Geltrex-coated T80-flasks to a confluency of 90%–100% and detached with EDTA. The formation of EBs was induced in 500 mL spinner flasks with a density of 30–35 × 10^6 hiPSCs per 100 mL of EB formation medium (Table S2). hiPSC suspension was cultivated overnight at 40 rpm glass ball impeller rotation speed. Mesoderm induction was induced in mesoderm induction medium (Table S2) with a volume of 200–300 μL EB per pluronic-coated T175-flask for 3 days under hypoxic conditions (5% O_2) with 50% medium exchange daily. After washing the EBs again, cardiac differentiation was induced in cardiac differentiation medium 1 (Table S2) with a volume of 250–300 μL EB per pluronic-coated T175-flask with 50% medium exchange daily for 3 days under normoxic conditions (21% O_2). Then, medium was completely removed and exchanged for cardiac differentiation medium 2 (Table S2). After a daily 50% medium change for 4 days, culturing medium was exchanged with cardiac differentiation medium 3 (Table S2). After washing EBs in HBBS solution buffer, beating cardiomyocytes were dissociated with collagenase II solution (200 units/mL; Worthington) containing myosin II ATPase inhibitor N-benzyl-p-toluene sulfonamide for 2–3 h until dispersing single cells could be observed. Dissociated hiPSC-CM were frozen in freezing medium containing 90% FBS and 10% DMSO or resuspended in EHT casting medium for subsequent EHT generation. Differentiation efficiency (% cTNT-positive cells) was determined by fluorescent-labeled cTNT antibody (Miltenyi Biotech) using a flow cytometer FACSCanto II (BD). Adjustment of gates adjusted according to the isotype control and performed with FACSDiva software (BD). Differentiation runs with at least 75% cTNT-positive cells were used for further functional experiments. FACS reagents are shown in Table S4.

EHTs

EHTs were generated as recently described (Mannhardt et al., 2016). In brief, dissociated hiPSC-CM were centrifuged (100 × *g*, 10 min) and resuspended in EHT casting medium containing DMEM, horse serum, and glutamine (Table S2). Polytetrafluorethylene (PTFE) spacers (EHT Technologies) were placed in a warm 2% (w/v) agarose/PBS solution in a 24-well plate. Agarose solidification at room temperature led to the formation of agarose molds. PTFE spacers were removed from the 24-well plates and flexible polydimethylsiloxane (PDMS) posts were placed on the 24-well plates so that pairs of elastic PDMS posts reached into each casting mold. Mastermix (100 μL) (Table S2) containing 2× DMEM, Y-27632, fibrinogen and 1.0 × 10^6 hiPSC-CMs was resuspended in EHT casting medium, rapidly mixed with 3 μL thrombin and pipetted into one agarose casting mold. Afterward the preparation was placed in an incubator for 1.5 h at 37°C until a fibrin gel formed in the agarose molds around the PDMS posts. Pre-warmed EHT culture medium (200–300 μL) was added dropwise into each well to ameliorate the detachment of the fibrin gel from the agarose mold. After an additional 15–30 min of incubation, racks with fibrin gels attached to the PDMS posts were transferred into a new 24-well plate, filled with pre-warmed EHT culture medium, and incubated at 40% O_2, 37°C, 7% CO_2, and 98% humidity. EHTs were cultured for 28–42 days with medium changes 3 times per week. After 5–7 days of culture, EHTs started to develop spontaneous macroscopic contractions.

Video-optical contraction analysis

EHT contractile analysis was performed as described previously (Mannhardt et al., 2016; Breckwoldt et al., 2017). EHT contraction parameters, e.g., force, frequency, and contraction kinetics, were monitored over time of EHT development 2 h after each medium change. EHTs were electrically stimulated as described previously by Hirt et al. (2014). PDMS racks with EHTs were mounted onto custom-made

graphite pacing units and stimulated by using a Grass S88X Dual Output Square stimulator (Natus Neurology Incorporated). The pacing frequency was adjusted to a value of 1.5- to 2-fold of the spontaneous beating frequency of the EHT batch with an output voltage of 2 V in biphasic pulses of 4 ms. EHTs that were not able to follow the pacing frequency were excluded from the analysis. Average contraction peaks were calculated with an average of 10–15 peaks.

SUPPLEMENTAL INFORMATION

Supplemental information can be found online at https://doi.org/10.1016/j.stemcr.2023.09.002.

ACKNOWLEDGMENTS

We greatly appreciate the assistance of the UKE FACS Core unit, the team approach of the hiPSC and CRISPR-Cas9 group at IEPT/UKE and the technical assistance of Anna Steenpaβ. This study was supported by the European Research Council (ERC-AG IndivuHeart), Deutsche Forschungsgemeinschaft (DFG Es 88/12-1, DFG HA 3423/5-1), the German Ministry of Education and Research (BMBF, PRAEDIKARD FKZ031L0236), the Centre for Cardiovascular Research (DZHK 81Z0710101, 81Z0710115, 81X2710176), and the Freie und Hansestadt Hamburg. M.M. is a British Heart Foundation (BHF) Chair Holder (CH/16/3/32406) with BHF program grant support (RG/16/14/32397) and is part of the Marie Skłodowska-Curie Innovative Training Network TRAIN-HEART (http://train-heart.eu).

AUTHOR CONTRIBUTIONS

CRISPR-Cas9, M.L., C.S.B., and B.M.U.; Southern blot, B.V.S. and T.S.R.; PCR, M.L.; cardiac differentiation, M.L., B.K., and T.S.; EHT generation and maintenance, M.L., B.K., and T.S.; force measurement, M.L.; action potential, C.S. and T.C.; proteomics/bioinformatics, M.M., K.T., X.Y., and M.L.; snRNA-seq, T.D.v.B., E.A., H.M., and N.H.; lipid analysis, S.B.; mtDNA-quantitative PCR, M.L. and K.S.; TEM, M.S.; Seahorse, S.B. and C.D.; concept, A.H., M.L., F.C., and T.E.; funding T.E. and A.H.; writing, M.L., A.H., and F.C.

DECLARATION OF INTERESTS

T.E. is a member of the DiNAQOR Scientific Advisory Board and holds shares in DiNAQOR.

Received: March 30, 2023
Revised: September 3, 2023
Accepted: September 4, 2023
Published: October 5, 2023

REFERENCES

Alesci, S., Manoli, I., Costello, R., Coates, P., Gold, P.W., Chrousos, G.P., and Blackman, M.R. (2004). Carnitine: Lessons from one hundred years of research. Ann. N. Y. Acad. Sci. *1033*.

Asai, T., Okumura, K., Takahashi, R., Matsui, H., Numaguchi, Y., Murakami, H., Murakami, R., and Murohara, T. (2006). Combined therapy with PPARα agonist and l-carnitine rescues lipotoxic cardiomyopathy due to systemic carnitine deficiency. Cardiovasc. Res. *70*, 566–577.

Bandet, C.L., and Hajduch, E. (2021). CERT-Dependent Ceramide Transport, A Crucial Process in Cells. J Diabetes Clin Res *3*, 40–45.

Breckwoldt, K., Letuffe-Brenière, D., Mannhardt, I., Schulze, T., Ulmer, B., Werner, T., Benzin, A., Klampe, B., Reinsch, M.C., Laufer, S., et al. (2017). Differentiation of cardiomyocytes and generation of human engineered heart tissue. Nat. Protoc. *12*, 1177–1197.

Broderick, T.L., Panagakis, G., DiDomenico, D., Gamble, J., Lopaschuk, G.D., Shug, A.L., and Paulson, D.J. (1995). L-carnitine improvement of cardiac function is associated with a stimulation in glucose but not fatty acid metabolism in carnitine-deficient hearts. Cardiovasc. Res. *30*, 815–820.

Chaffin, M., Papangeli, I., Simonson, B., Akkad, A.D., Hill, M.C., Arduini, A., Fleming, S.J., Melanson, M., Hayat, S., Kost-Alimova, M., et al. (2022). Single-nucleus profiling of human dilated and hypertrophic cardiomyopathy. Nature *608*, 174–180.

Chen, X., Kang, R., Kroemer, G., and Tang, D. (2021). Broadening horizons: the role of ferroptosis in cancer. Nat. Rev. Clin. Oncol. *18*, 280–296.

Concordet, J.P., and Haeussler, M. (2018). CRISPOR: Intuitive guide selection for CRISPR/Cas9 genome editing experiments and screens. Nucleic Acids Res. *46*, W242–W245.

Cooper, D.E., Young, P.A., Klett, E.L., and Coleman, R.A. (2015). Physiological consequences of compartmentalized acyl-CoA metabolism. J. Biol. Chem. *290*, 20023–20031.

Degrace, P., Demizieux, L., Gresti, J., Tsoko, M., André, A., Demaison, L., and Clouet, P. (2004). Fatty acid oxidation and related gene expression in heart depleted of carnitine by mildronate treatment in the rat. Mol. Cell. Biochem. *258*, 171–182.

Dixon, S.J., Lemberg, K.M., Lamprecht, M.R., Skouta, R., Zaitsev, E.M., Gleason, C.E., Patel, D.N., Bauer, A.J., Cantley, A.M., Yang, W.S., et al. (2012). Ferroptosis: An iron-dependent form of nonapoptotic cell death. Cell *149*, 1060–1072.

Doll, S., Proneth, B., Tyurina, Y.Y., Panzilius, E., Kobayashi, S., Ingold, I., Irmler, M., Beckers, J., Aichler, M., Walch, A., et al. (2017). ACSL4 dictates ferroptosis sensitivity by shaping cellular lipid composition. Nat. Chem. Biol. *13*, 91–98.

Fang, X., Wang, H., Han, D., Xie, E., Yang, X., Wei, J., Gu, S., Gao, F., Zhu, N., Yin, X., et al. (2019). Ferroptosis as a target for protection against cardiomyopathy. Proc. Natl. Acad. Sci. USA *116*, 2672–2680.

Friedmann Angeli, J.P., Schneider, M., Proneth, B., Tyurina, Y.Y., Tyurin, V.A., Hammond, V.J., Herbach, N., Aichler, M., Walch, A., Eggenhofer, E., et al. (2014). Inactivation of the ferroptosis regulator Gpx4 triggers acute renal failure in mice. Nat. Cell Biol. *16*, 1180–1191.

Gong, Y., Wang, N., Liu, N., and Dong, H. (2019). Lipid Peroxidation and GPX4 Inhibition Are Common Causes for Myofibroblast Differentiation and Ferroptosis. DNA Cell Biol. *38*, 725–733.

Hirt, M.N., Boeddinghaus, J., Mitchell, A., Schaaf, S., Börnchen, C., Müller, C., Schulz, H., Hubner, N., Stenzig, J., Stoehr, A., et al. (2014). Functional improvement and maturation of rat and human engineered heart tissue by chronic electrical stimulation. J. Mol. Cell. Cardiol. *74*, 151–161.

Horiuchi, M., Kobayashi, K., Masuda, M., Terazono, H., and Saheki, T. (1999). Pyruvate dehydrogenase kinase 4 mRNA is increased in the hypertrophied ventricles of carnitine-deficient juvenile visceral steatosis (JVS) mice. Biofactors *10*, 301–309.

Horiuchi, M., Yoshida, H., Kobayashi, K., Kuriwaki, K., Yoshimine, K., Tomomura, M., Koizumi, T., Nikaido, H., Hayakawa, J., Kuwajima, M., et al. (1993). Cardiac hypertrophy in juvenile visceral steatosis (jvs) mice with systemic carnitine deficiency. FEBS Lett. *326*, 267–271.

Koeth, R.A., Wang, Z., Levison, B.S., Buffa, J.A., Org, E., Sheehy, B.T., Britt, E.B., Fu, X., Wu, Y., Li, L., et al. (2013). Intestinal microbiota metabolism of l-carnitine, a nutrient in red meat, promotes atherosclerosis. Nat. Med. *19*, 576–585.

Koizumi, T., Nikaido, H., Hayakawa, J., Nonomura, A., and Yoneda, T. (1988). Infantile disease with microvesicular fatty infiltration of viscera spontaneously occurring in the C3H-H-2° strain of mouse with similarities to Reye's syndrome. Lab. Anim. *22*, 83–87.

Li, D., and Li, Y. (2020). The interaction between ferroptosis and lipid metabolism in cancer. Signal Transduct. Targeted Ther. *5*, 108–110.

Liepinsh, E., Vilskersts, R., Skapare, E., Svalbe, B., Kuka, J., Cirule, H., Pugovics, O., Kalvinsh, I., and Dambrova, M. (2008). Mildronate decreases carnitine availability and up-regulates glucose uptake and related gene expression in the mouse heart. Life Sci. *83*, 613–619.

Longo, N., Amat Di San Filippo, C., Pasquali, M., Filippo, C.A., di, S., and Pasquali, M. (2006). Disorders of carnitine transport and the carnitine cycle. Am. J. Med. Genet. C Semin. Med. Genet. *142C C*, 77–85.

Longo, N., Frigeni, M., and Pasquali, M. (2016). Carnitine transport and fatty acid oxidation. Biochim. Biophys. Acta *1863*, 2422–2435.

MacEyka, M., and Spiegel, S. (2014). Sphingolipid metabolites in inflammatory disease. Nature *510*, 58–67.

Magoulas, P.L., and El-Hattab, A.W. (2012). Systemic primary carnitine deficiency: An overview of clinical manifestations, diagnosis, and management. Orphanet J. Rare Dis. *7*, 68. https://doi.org/10.1186/1750-1172-7-68.

Mannhardt, I., Breckwoldt, K., Letuffe-Brenière, D., Schaaf, S., Schulz, H., Neuber, C., Benzin, A., Werner, T., Eder, A., Schulze, T., et al. (2016). Human Engineered Heart Tissue: Analysis of Contractile Force. Stem Cell Rep. *7*, 29–42.

Mannhardt, I., Saleem, U., Mosqueira, D., Loos, M.F., Ulmer, B.M., Lemoine, M.D., Larsson, C., Améen, C., de Korte, T., Vlaming, M.L.H., et al. (2020). Comparison of 10 Control hPSC Lines for Drug Screening in an Engineered Heart Tissue Format. Stem Cell Rep. *15*, 983–998.

Nicin, L., Abplanalp, W.T., Schänzer, A., Sprengel, A., John, D., Mellentin, H., Tombor, L., Keuper, M., Ullrich, E., Klingel, K., et al. (2021). Single Nuclei Sequencing Reveals Novel Insights into the Regulation of Cellular Signatures in Children with Dilated Cardiomyopathy. Circulation *143*, 1704–1719.

Rasmussen, J., Dunø, M., Lund, A.M., Steuerwald, U., Hansen, S.H., Joensen, H.D., Køber, L., and Nielsen, O.W. (2020). Increased risk of sudden death in untreated primary carnitine deficiency. J. Inherit. Metab. Dis. *43*, 290–296.

Rasmussen, J., Nielsen, O.W., Janzen, N., Duno, M., Køber, L., Køber, L., Steuerwald, U., and Lund, A.M. (2014). Carnitine levels in 26,462 individuals from the nationwide screening program for primary carnitine deficiency in the Faroe Islands. J. Inherit. Metab. Dis. *37*, 215–222.

Rebouche, C.J. (2004). Kinetics, pharmacokinetics, and regulation of L-carnitine and acetyl-L-carnitine metabolism. Ann. N. Y. Acad. Sci. *1033*, 30–41.

Roussel, J., Labarthe, F., Thireau, J., Ferro, F., Farah, C., Roy, J., Horiuchi, M., Tardieu, M., Lefort, B., François Benoist, J., et al. (2016). Carnitine deficiency induces a short QT syndrome. Hear Rhythm *13*, 165–174.

Saburi, Y., Okumura, K., Matsui, H., Hayashi, K., Kamiya, H., Takahashi, R., Matsubara, K., and Ito, M. (2003). Changes in distinct species of 1,2-diacylglycerol in cardiac hypertrophy due to energy metabolic disorder. Cardiovasc. Res. *57*, 92–100.

Shibamiya, A., Schulze, E., Krauß, D., Augustin, C., Reinsch, M., Schulze, M.L., Steuck, S., Mearini, G., Mannhardt, I., Schulze, T., et al. (2020). Cell Banking of hiPSCs: A Practical Guide to Cryopreservation and Quality Control in Basic Research. Curr Protoc Stem Cell Biol *55*, 1–26.

Spaniol, M., Brooks, H., Auer, L., Zimmermann, A., Solioz, M., Stieger, B., and Krähenbühl, S. (2001). Development and characterization of an animal model of carnitine deficiency. Eur. J. Biochem. *268*, 1876–1887.

Tamai, I., Ohashi, R., Nezu, J., Yabuuchi, H., Oku, A., Shimane, M., Sai, Y., and Tsuji, A. (1998). Molecular and functional identification of sodium ion-dependent, high affinity human carnitine transporter OCTN2. J. Biol. Chem. *273*, 20378–20382.

Tan, J., Wagner, M., Stenton, S.L., Strom, T.M., Wortmann, S.B., Prokisch, H., Meitinger, T., Oexle, K., and Klopstock, T. (2020). Lifetime risk of autosomal recessive mitochondrial disorders calculated from genetic databases. EBioMedicine *54*, 102730.

Tomlinson, S., Atherton, J., and Prasad, S. (2018). Primary Carnitine Deficiency: A Rare, Reversible Metabolic Cardiomyopathy. Case Reports Cardiol *2018*, 232105.

Tomomura, M., Imamura, Y., Horiuchi, M., Koizumi, T., Nikaido, H., Hayakawa, J., and Saheki, T. (1992). Abnormal expression of urea cycle enzyme genes in juvenile visceral steatosis (jvs) mice. Biochim. Biophys. Acta *1138*, 167–171.

Wagner, C.A., Lükewille, U., Kaltenbach, S., Moschen, I., Bröer, A., Risler, T., Bröer, S., and Lang, F. (2000). Functional and pharmacological characterization of human Na+-carnitine cotransporter hOCTN2. Am. J. Physiol. Ren. Physiol. *279*, F584–F591.

Wang, S.S., Rao, J., Li, Y.F., Zhang, Z.W., and Zeng, G.H. (2014). Primary carnitine deficiency cardiomyopathy. Int. J. Cardiol. *174*, 171–173.

Wu, J., and Corr, P.B. (1994). Palmitoyl transient carnitine modifies sodium currents and induces inward current in ventricular myocytes. Am. J. Physiol. *266*, 1034–1046.

Yagoda, N., Von Rechenberg, M., Zaganjor, E., Bauer, A.J., Yang, W.S., Fridman, D.J., Wolpaw, A.J., Smukste, I., Peltier, J.M., Boniface, J.J., et al. (2007). RAS-RAF-MEK-dependent oxidative cell death involving voltage-dependent anion channels. Nature *447*, 864–868.

Yang, W.S., Kim, K.J., Gaschler, M.M., Patel, M., Shchepinov, M.S., and Stockwell, B.R. (2016). Peroxidation of polyunsaturated fatty acids by lipoxygenases drives ferroptosis. Proc. Natl. Acad. Sci. USA *113*, E4966–E4975.

Zhang, B., Chen, X., Ru, F., Gan, Y., Li, B., Xia, W., Dai, G., He, Y., and Chen, Z. (2021). Liproxstatin-1 attenuates unilateral ureteral obstruction-induced renal fibrosis by inhibiting renal tubular epithelial cells ferroptosis. Cell Death Dis. *12*, 843.

Stem Cell Reports, Volume *18*

Supplemental Information

Human model of primary carnitine deficiency cardiomyopathy reveals ferroptosis as a novel mechanism

Malte Loos, Birgit Klampe, Thomas Schulze, Xiaoke Yin, Konstantinos Theofilatos, Bärbel Maria Ulmer, Carl Schulz, Charlotta S. Behrens, Tessa Diana van Bergen, Eleonora Adami, Henrike Maatz, Michaela Schweizer, Susanne Brodesser, Boris V. Skryabin, Timofey S. Rozhdestvensky, Sara Bodbin, Konstantina Stathopoulou, Torsten Christ, Chris Denning, Norbert Hübner, Manuel Mayr, Friederike Cuello, Thomas Eschenhagen, and Arne Hansen

Supplementary data file

Supplementary figures

Supplementary Figure 1

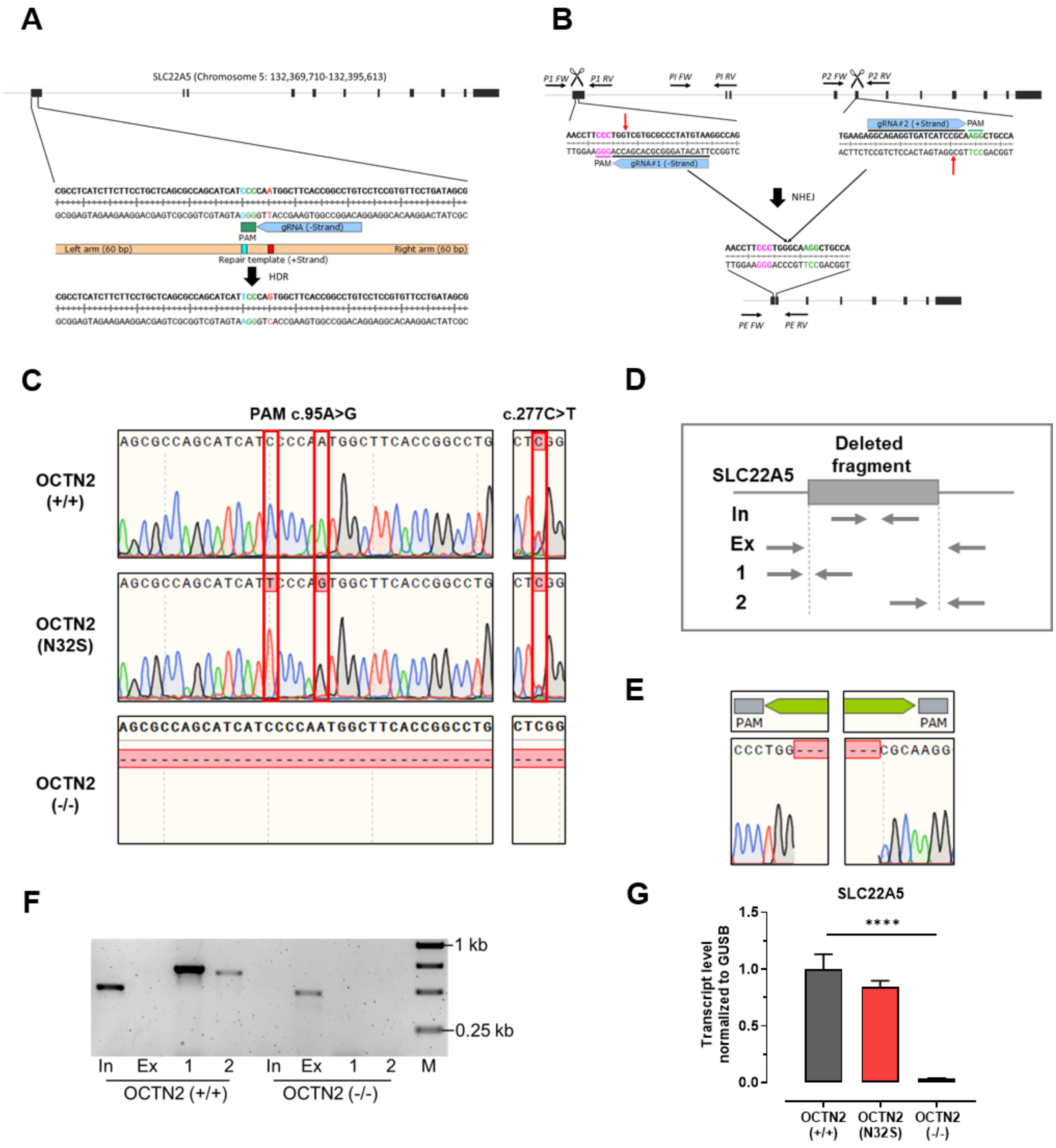

Supplementary Figure 1: Genotype characterization of CRISPR/Cas9-edited hiPSCs. A: Schematic overview of CRISPR/Cas9 strategy for OCTN2 (N32S) generation. A ssODN containing the desired point mutation was co-transfected with CRISPR components to introduce the mutation c.95A>G, p.N32S in exon1 of the SLC22A5 gene. Depicted is the gRNA target site and the predictive DNA sequence after successful integration of the repair template. **B**: Schematic overview of the CRISPR/Cas9 strategy for OCTN2 (-/-) generation. Two gRNA were co-transfected to induce simultaneous cutting and a large deletion in the SLC22A5 gene.

Depicted are the gRNA target sites and the predicted DNA sequence after successful editing. Red arrows indicate the predicted Cas9 cutting sites. Black arrows indicate the primer binding sites for PCR validation. P1: Primer target region gRNA1; P2: Primer target region gRNA2; PI: Primer internal; PE: Primer external; Pink: PAM1; Green: PAM2. **C**: Sanger sequencing traces of OCTN2 (+/+) and derived single cell clones for OCTN2 (N32S) and OCTN2 (-/-) genotypes. The red boxes indicate the silent PAM- and c.95A>G mutation and the heterozygous silent c277C>T mutation in the OCTN2 (N32S) clone. **D**: Schematic overview of primer localization in relation to the deleted fragment in the *SLC22A5* gene. **E**: Sanger sequencing trace of OCTN2 (-/-) PCR product amplified with external primers. **F**: Gel electrophoresis of PCR products of OCTN2 (+/+) and OCTN2 (-/-) hiPSC clones; M = 1 kb DNA standard marker. Depicted are the binding sites of both gRNA. **G**: qPCR analysis of *SLC22A5* transcripts for all genotypes.

Supplementary Figure 2

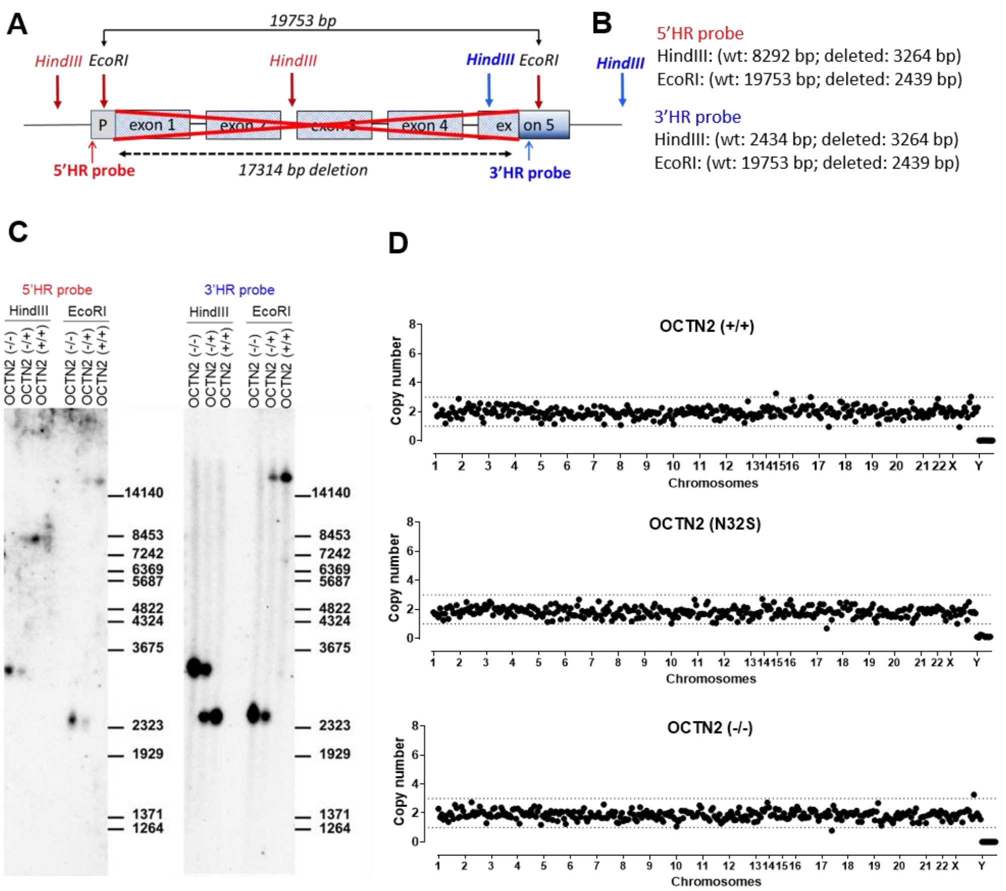

Supplementary Figure 2. **Southern blot and Nanostring analysis of genomic DNA from OCTN2 (+/+), OCTN2 (-/+) and OCTN2 (-/-) hiPSC. A:** Schematic presentation of Southern blot probes hybridization position. HindIII and EcoRI enzymes were used for enzymatic digestion of genomic DNA samples. **B**: Fragment size prediction of 5' HR and 3'HR hybridization after HindIII or EcoRI restriction enzyme digest. **C**: Southern blot analysis of OCTN2 (+/+), OCTN2 (-/+) and OCTN2 (-/-) hiPSC. **D:** Nanostring karyotype analysis of hiPSC master cell bank samples. OCTN2 (+/+), OCTN2 (N32S) and OCTN2 (-/-).

Supplementary Figure 3

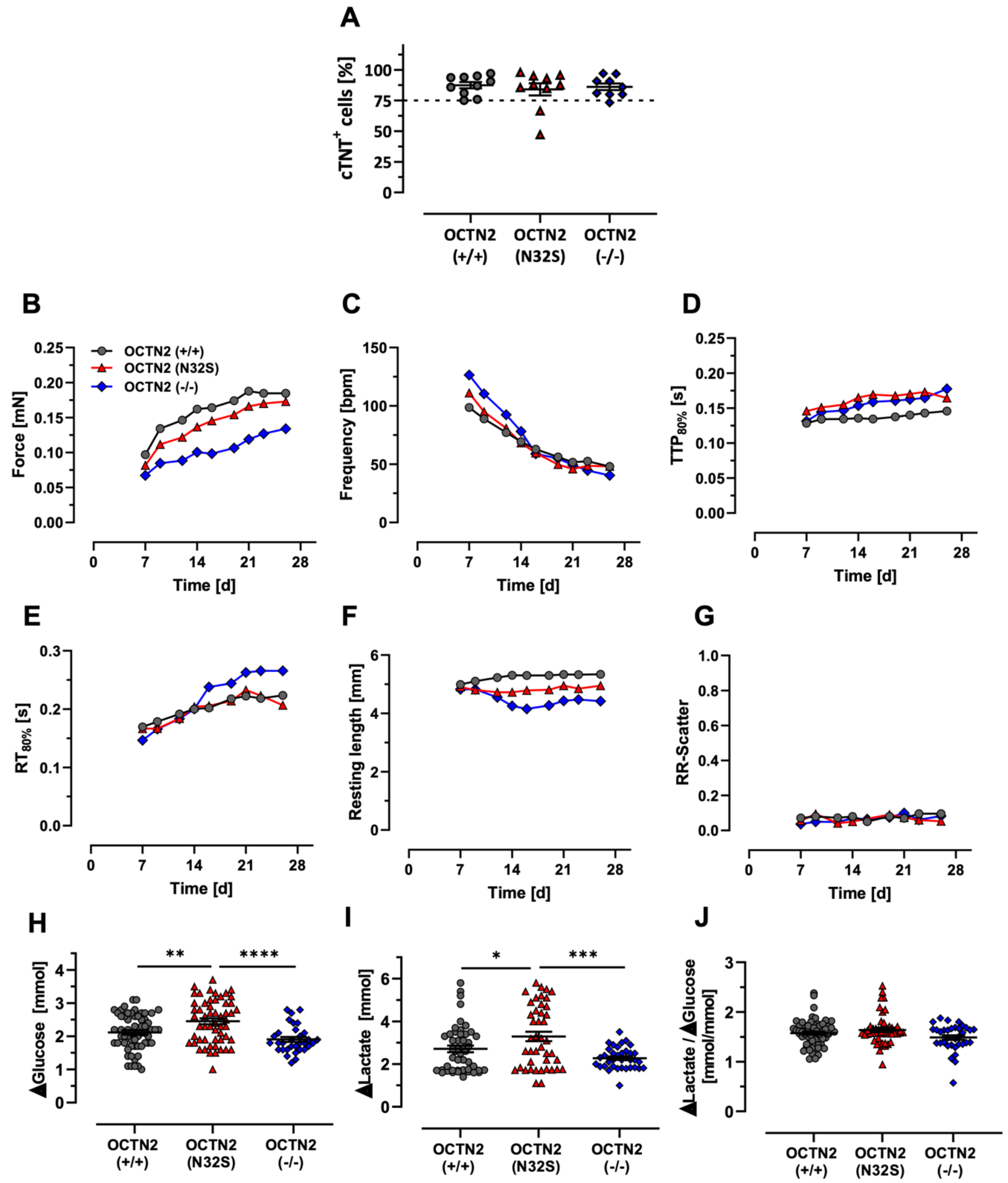

Supplementary Figure 3. Characterization of cardiomyocyte differentiations, contractile and metabolic parameters. A: FACS analysis of cardiac troponin T positive cells in the differentiated cell population. Dashed line (75%) indicates threshold value for EHT generation. OCNT2 (+/+): n=10, OCTN2 (N32S): n=10, OCTN2 (-/-): n=9 differentiation batches, data are expressed as mean±SEM, 1-way ANOVA followed by Bonferroni's post-test for multiple comparisons, ****p<0.0001. **B-G:** Effect of OCTN2 genotype on force, frequency, contraction time (TTP80), relaxation time (RT80), resting length and RR-Scatter (parameter of irregularity) of spontaneous beating EHTs between day 7 and day 26. OCNT2 (+/+): n=152 EHTs from 9 batches, OCTN2 (N32S): n=108 EHTs from 7 batches, OCTN2 (-/-): n=91 EHTs from 5 batches, data are expressed as EHT batch mean±SEM. **H:** Difference in Glucose- and **I:** Lactate concentration of EHT culture media (Δglucose = glucose concentration at baseline minus

glucose concentration after 24 h of incubation, Δlactate = lactate concentration at baseline minus lactate concentration after 24 h of incubation). **J:** ΔLactate of EHT culture media divided by Δglucose. OCNT2 (+/+): n=59 EHTs from 5 batches, OCTN2 (N32S): n=51 EHTs from 4 batches, OCTN2 (-/-): n=28 EHTs from 4 batches. 1-way ANOVA followed by Bonferroni's post-test for multiple comparisons, *$p<0.05$, **$p<0.01$, ***$p<0.001$, ****$p<0.0001$. One data point represents one independent EHT. Data are expressed as mean±SEM. Related to Figure 1 and 2.

Supplementary Figure 4

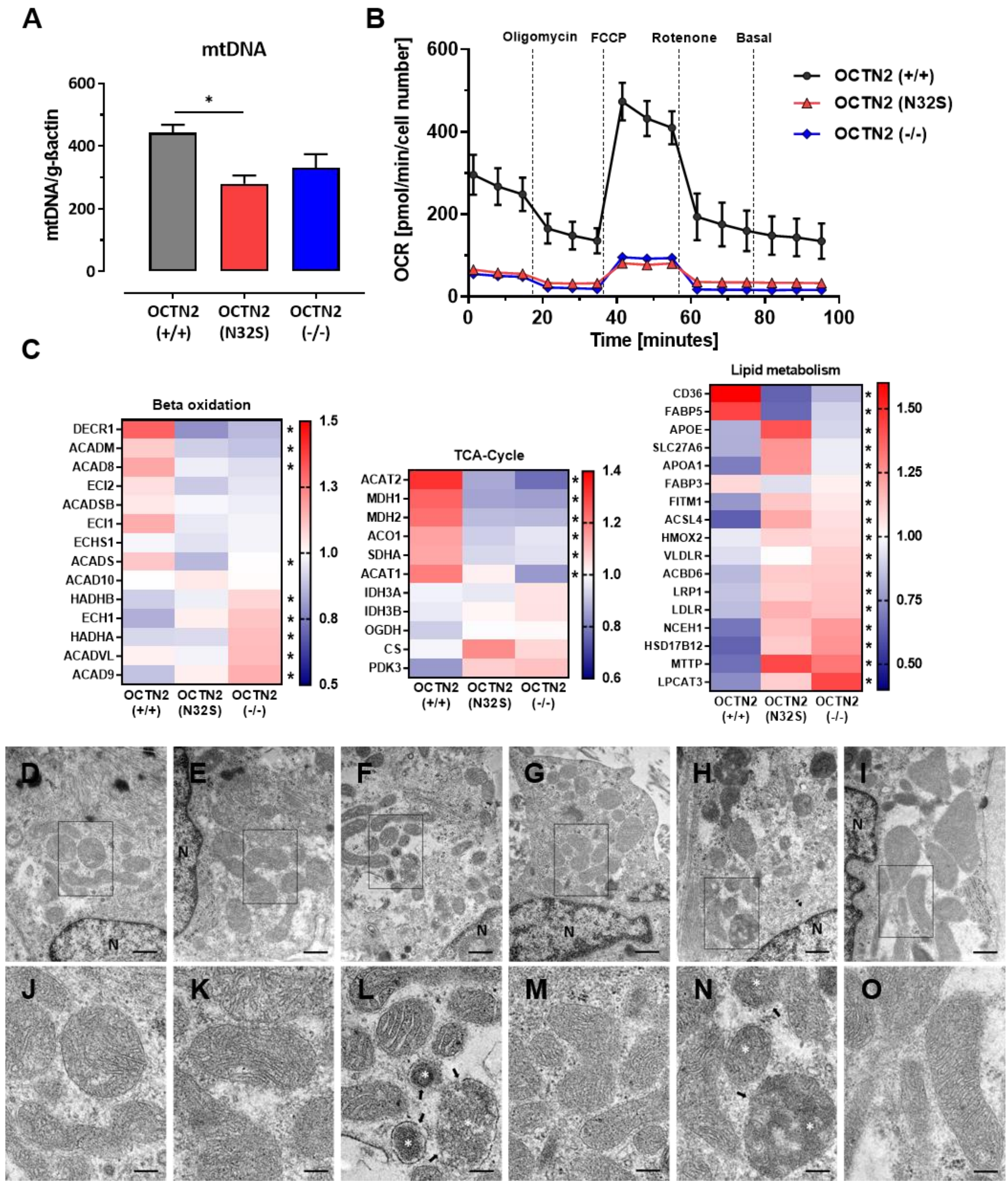

Supplementary Figure 4. A: Effect of OCTN2 genotype on mitochondrial DNA analyzed by quantitative PCR. MtDNA was normalized to nuclear encoded globular actin (g-actin). OCNT2 (+/+): n=5 EHTs from 1 batch, OCTN2 (N32S): n=7 EHTs from 1 batch, OCTN2 (-/-): n=7 EHTs from 1 batch. 1-way ANOVA followed by Bonferroni's post-test for multiple comparisons, *p<0.05. Data are expressed as mean±SEM. **B**: Oxygen consumption rate in OCNT2 (+/+), (N32S) and (-/-). Mean ± SEM, n=2 biological replicates (each biological replicate represents the average of 12 wells of a 96 well Seahorse plate), Mann-Whitney U test, *p<0.05. **C:** Clustering analysis of proteins related to metabolic pathways. Heatmaps display the relative abundance of proteins involved in the electron transport chain (ETC), beta-oxidation and lipid metabolism. OCNT2 (+/+): mean of 10 EHTs from 1 batch; OCTN2 (N32S): mean of 10 EHTs from 1 batch; OCTN2 (-/-): mean of 10 EHTs from 1 batch. Protein levels are depicted as a

color code ranging from blue (low abundance) to red (high abundance). Kruskal-Wallis test, * indicates statistically significant difference of OCTN2 (+/+) against OCTN2 (N32S) or OCTN2 (-/-). **Signs of Ferroptosis EM Transmission electron microscopy.** D + E: (+/+), F + G: OCTN2 (N32S), H + I: OCTN2 (-/-). D, F and H untreated, E, G and I supplemented with carnitine (2mM). J to O: enlargements of D to I, respectively. Mitochondria of OCTN2 mutants (F, L) and knock-out cells (H, N) show morphological features indicating ferroptosis like increased membrane matrix density and absent cristae (* in L, N), rupture and fragmentation of the outer membrane (arrows in L, N). Nuclear morphology did not display any changes (N). Scale bars 500nm D to I and 200nm J to L. Related to Figure 3 and 5.

Supplementary Figure 5

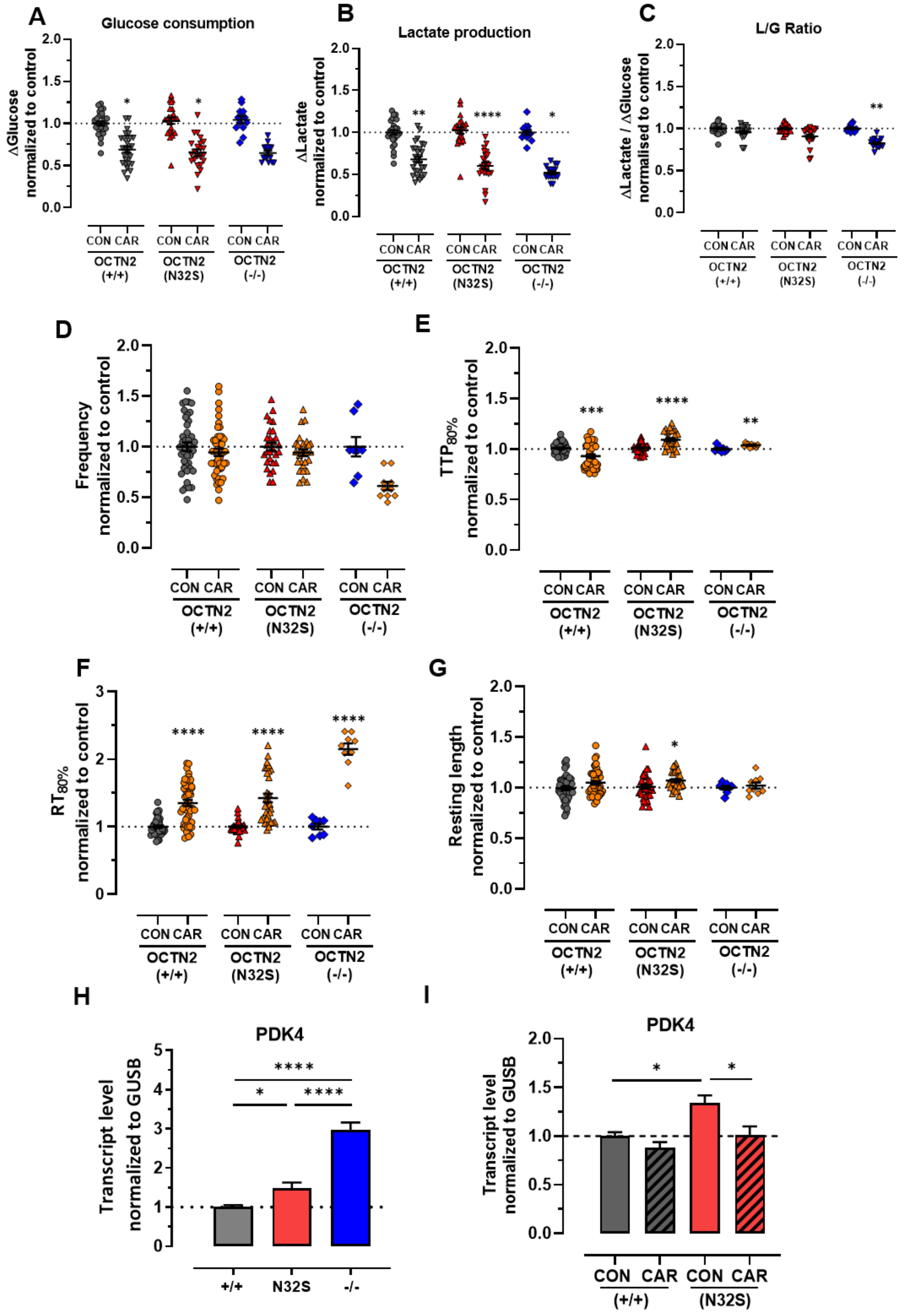

Supplementary Figure 5. A-C: Effect of carnitine supplementation on glucose consumption and lactate production, contractile parameters and PDK4 mRNA expression. A: ΔGlucose (ΔGlucose= Glucose concentration at baseline minus glucose concentration after 24 hours of incubation); **B**: ΔLactate (ΔLactate= Lactate concentration after 24 hours of incubation minus lactate concentration at baseline). Nested t-test vs CON, **p<0.01, ***p<0.001, ****p<0.0001. OCNT2 (+/+) control: n=27 EHTs from 3 batches. OCNT2 (+/+) + carnitine (2 mM): n=28 EHTs from 3 batches. OCTN2 (N32S) control: n=23 EHTs from 3 batches. OCTN2 (N32S) + carnitine (2 mM): n=23 EHTs from 3 batches, OCTN2 (-/-) control: n=13 EHTs from 3 batches, OCTN2 (-/-) + carnitine (2 mM): n=16 EHTs from 3 batches. Data are expressed as mean±SEM. **D-G: Effect of carnitine supplementation on spontaneous beating EHTs at the last day of treatment (Day 33-42). D**: Frequency, **E**: Time to peak, **F**: Relaxation time and **G**: Resting length. Values were normalized to last day of treatment of untreated control. Student's t-test vs CON, **p<0.01, ***p<0.001, ****p<0.001. OCNT2 (+/+) control: n=54 EHTs from 4 batches. OCNT2 (+/+) + carnitine (2 mM): n=49 EHTs from 4 batches. OCTN2 (N32S) control: n=36 EHTs from 3 batches, OCTN2 (N32S) + carnitine (2 mM): n=33 EHTs from 3 batches, OCTN2 (-/-) control: n=9 EHTs from 1 batch, OCTN2 (-/-) + carnitine (2 mM): n=9 EHTs from 1 batch. Data are expressed as mean±SEM. **H: Effect of OCTN2 genotype on PDK4 mRNA expression by quantitative RT-PCR analysis. Gene expression was normalized to GUSB over OCTN2 (+/+) control.** OCNT2 (+/+): n=8 EHTs from 2 batches, OCTN2 (N32S): n=8 EHTs from 2 batches; OCTN2 (-/-): n=6 EHTs from 2 batches. 1-way ANOVA plus Bonferroni's post-test for multiple comparisons, *p<0.05, ****p<0.0001. Data are expressed as mean±SEM. **I: Effect of carnitine supplementation on PDK4 mRNA expression.** OCNT2 (+/+) and OCTN2 (N32S) EHTs were treated over the entire culture time harvested on day 42. Gene expression was normalized to GUSB over OCTN2 (+/+) control. n=7 EHTs per genotype and carnitine treatment from 1 batch. 2-way ANOVA followed by Bonferroni's post-test for multiple comparisons, *p<0.05. Data are expressed as mean±SEM. Related to Figure 4.

Supplementary Figure 6

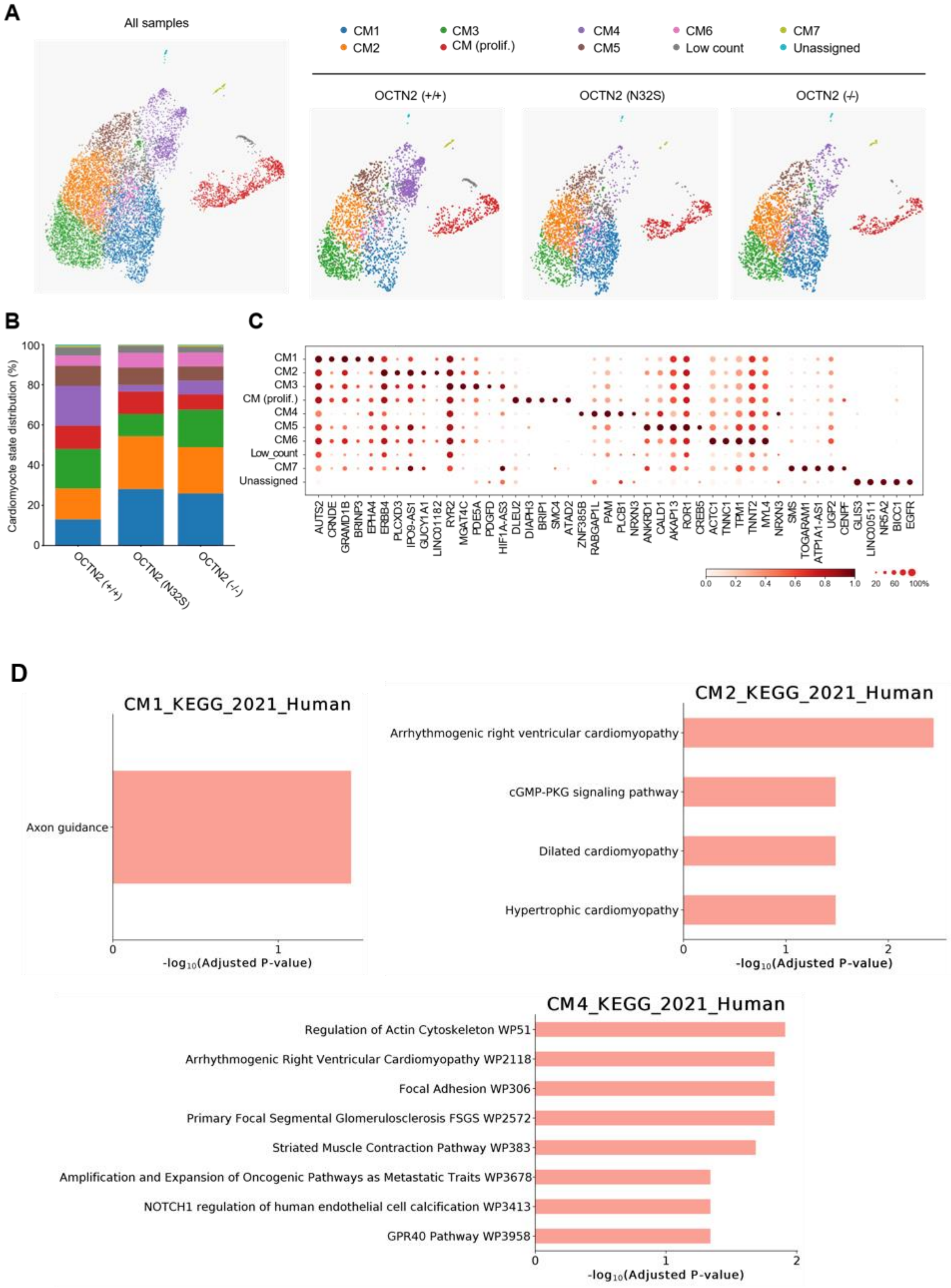

Supplementary Figure 6. Cardiomyocyte and fibroblast subcluster analysis in OCTN2 genotypes. A: Representative UMAP plot after single-nucleus RNA sequencing of all samples and individual

genotypes. **B:** Percentage of cardiomyocyte subcluster per genotype. **C:** Dot plot graph showing the relative expression of upregulated genes per cell cluster. Expression levels are depicted as a color code ranging from light red (low expression) to dark red (high expression) as mean of log2 fold of expression. **D:** Enrichment analysis of significantly upregulated genes from cardiomyocyte subclusters CM1, CM2, CM4. Related to Figure 6.

Supplementary Figure 7

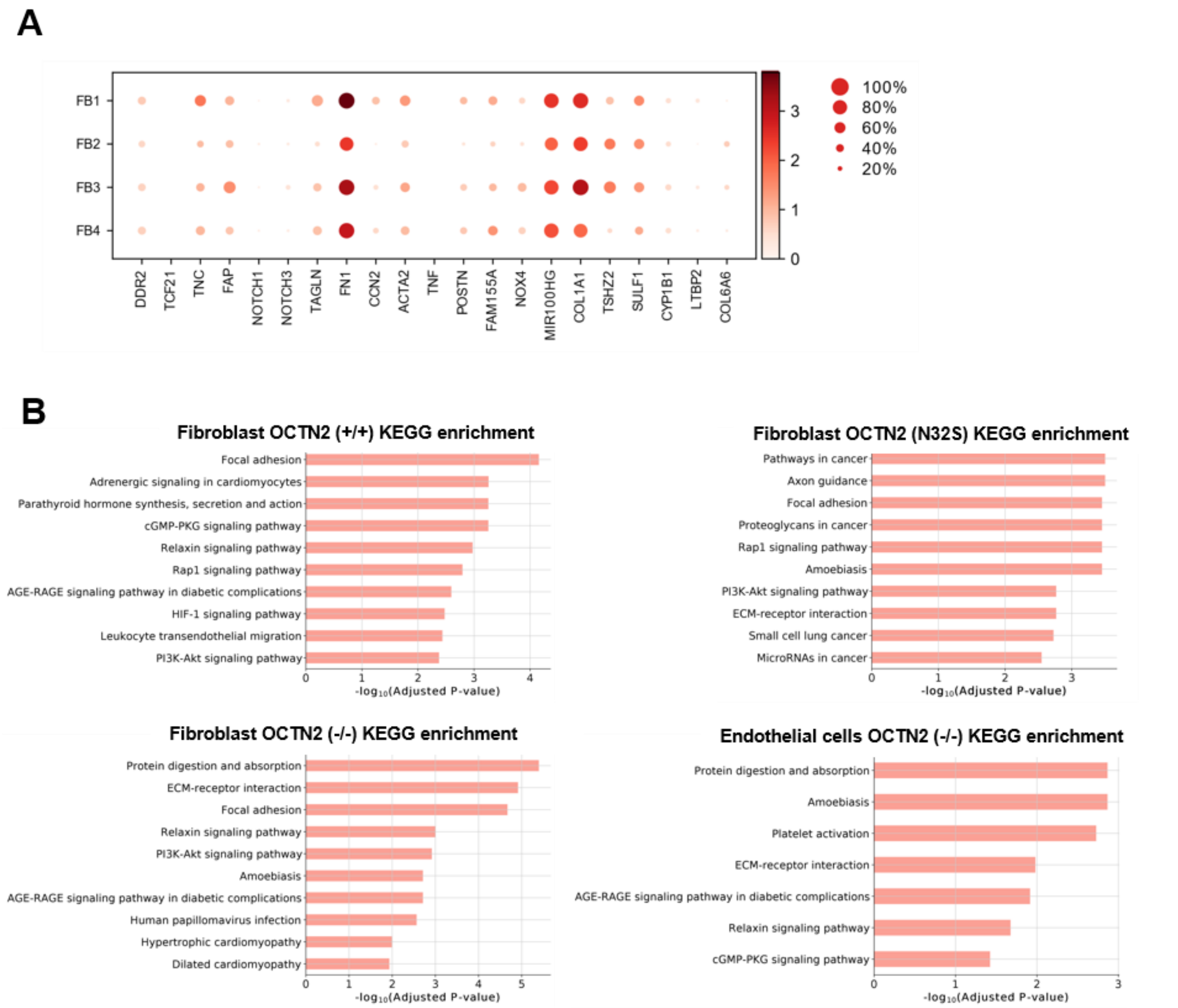

Supplementary Figure 7. Fibroblast and endothelial cell subcluster analysis in OCTN2 genotypes. A: Dot plot graph showing the relative expression of specific marker genes related fibroblast activation in fibroblast states. Unscaled expression levels are depicted as a color code ranging from light red (low expression) to dark red (high expression) as mean of log2 fold of expression. **B:** Enrichment analysis of significantly upregulated genes from fibroblast and endothelial cell subclusters. OCTN2 (+/+) and OCTN2 (N32S) endothelial cell and all myeloid subclusters did not reveal significant enriched pathways. Related to Figure 6 and 7.

Supplementary tables

Supplementary table 2. Composition of stem cell related culture media, related to Figure 1

**Medium**	**Components**	
Conditioned Medium (CDM)	DMEM/F12 without glutamine<br>1% (v/v) Non-essential amino acids<br>1% (v/v) L-glutamine<br>0.5% (v/v) Penicillin/streptomycin<br>3.5 µL/500 mL 2-Mercaptoethanol<br>20% (v/v) Knockout serum replacement<br>10 ng/mL Basic fibroblast growth factor (bFGF)	After incubation on mitotically inactivated mouse embryonic fibroblasts (strain CF-1) for 24 hours, CDM medium was collected and sterile filtered (0.1 µm filter). Fresh bFGF (30 ng/mL) was supplemented directly before use.
FTDA	DMEM/F-12 without glutamine<br>2 mM L-glutamine<br>0.1% (v/v) Lipid mix<br>5 mg/L Transferrin<br>5 µg/L Selenium<br>0.1% (v/v) Human serum albumin	5 µg/mL Insulin<br>2.5 ng/mL Activin-A<br>30 ng/mL bFGF<br>50 nM Dorsomorphin<br>0.5 ng/mL TGFß1
Mesoderm induction medium	RPMI 1640<br>4 mg/mL Polyvinyl alcohol<br>10 mM HEPES<br>0.05% (v/v) Human serum albumin<br>250 µM Phosphoascorbate<br>5 mg/l Transferrin	5 µg/l Selenium<br>0.1% (v/v) Lipid mix<br>10 µM Y-27632<br>3 ng/mL Activin-A<br>10 ng/mL BMP4<br>5 ng/mL bFGF
EB formation medium	FTDA<br>4 mg/mL Polyvinyl alcohol<br>10 µM Y-27632	
Cardiac differentiation medium I	RPMI 1640<br>10 mM HEPES<br>0.5% (v/v) Penicillin/streptomycin<br>0.05% (v/v) Human serum albumin<br>250 µM Phosphoascorbate	5 mg/L Transferrin<br>5 µg/L Selenium<br>0.1% (v/v) Lipid mix<br>1 µM Y-27632<br>1 µM XAV-939
Cardiac differentiation medium II	RPMI 1640<br>2% (v/v) B27 plus insulin<br>10 mM HEPES<br>0.5% (v/v) Penicillin/streptomycin	500 µM 1-Thioglycerol<br>1 µM Y-27632<br>1 µM XAV-939
Cardiac differentiation medium III	RPMI 1640,<br>2% (v/v) B27 plus insulin<br>10 mM HEPES	0.5% (v/v) Penicillin/streptomycin<br>500 µM 1-Thioglycerol<br>1 µM Y-27632

Supplementary Table 2ff. Composition of stem cell related culture media

Medium	Components	
EHT casting medium	DMEM<br>1% (v/v) Penicillin/streptomycin	2 mM L-Glutamine<br>10% (v/v) Horse serum (heat inactivated)
EHT culture medium	DMEM<br>1% (v/v) Penicillin/streptomycin<br>10% (v/v) Horse serum	10 µg/mL Insulin<br>33 µg/mL Aprotinin<br>0.2 mM Transexamic acid
Fatty acid medium	DMEM (without Glucose)<br>1% (v/v) Penicillin/streptomycin<br>10 µg/mL Insulin<br>33 µg/mL Aprotinin<br>0.2 mM Transexamic acid	50 ng/mL Hydrocortisone<br>0.5 ng/mL T_3<br>0.2 mM L-Glutamine<br>50 µM L-Carnitine hydrochloride<br>1 mg/mL Linoleic Acid-Oleic Acid-Albumin
Tyrode's solution	120 mM NaCl<br>5.4 mM KCl<br>1 mM $MgCl_2$ x 6·H2O<br>0.4 mM NaH_2PO_4	22.6 mM $NaHCO_3$<br>5 mM Glucose<br>0.05 mM Na_2EDTA<br>25 mM HEPES (pH 7.4, in PBS)
Mastermix EHT casting	hiPSC-CM<br>EHT casting medium<br>2x DMEM<br>0.1% Y-27632<br>Fibrinogen	$1.0x10^6$ per EHT<br>97.68 µL per EHT<br>6.125 µL per EHT<br>0.11 µL per EHT<br>2.78 µL per EHT

Supplementary Table 4. Primary antibodies used for flow cytometry/FACS, related to Supplementary Figure 3

Antibody	Dilution	Supplier and Cat#
Anti-cardiac troponin T-FITC	1:50	Miltenyi Biotec, 130-119-674
REA Control (I)-FITC	1:50	Miltenyi Biotec, 130-120-709
Rat Anti-Human SSEA-3 Antibody, PE Conjugated	1:50	BD Biosciences, 560237
Rat IgM, κ Isotype Control Antibody, PE Conjugated	1:50	BD Biosciences, 553943

Experimental procedures

Nucleofection

The AmaxaTM P3 Primary Cell 4D-Nucleofector X Kit L (Lonza) was used for delivery of the CRISPR/Cas9 ribonucleoprotein (RNP) complex into hiPSCs. A working cell bank aliquot of the control hiPSC (passage 25-30) was cultured for at least 2 passages on a 6-well plate to reach 60-70% confluency on the day of nucleofection. HiPSCs were incubated with the apoptosis inhibitor Y-27632 (10 µM) two hours prior to nucleofection. The fluorescence-labeled tracrRNA-ATTO 550 (IDT) was used to monitor the electroporation efficiency. The tracrRNA oligos and the CRISPR-Cas9 crRNA (IDT) oligos were resuspended in RNAse-free IDTE Buffer (IDT) to a final stock concentration of 100 µM. For gRNA duplex formation, 5 µL of crRNA (100 µM) were annealed with 5 µL tracrRNA (100 µM), incubated for 5 min at 95 °C and cooled down to room temperature. For formation of the RNP- complex, 5 µL of the gRNA duplex were mixed with 5 µL Cas9 protein (61 µM, IDT) and incubated for 1.5 hours at room temperature under light protection. For the knockout approach 5 µL of the second gRNA duplex were added to the suspension in addition. To prepare the nucleofector solution, 82 µL P3 reagent and 18 µL supplement reagent (Lonza) were mixed per reaction according to the instruction of the AmaxaTM P3 Primary Cell 4D-Nucleofector X Kit L (Lonza). HiPSCs were washed twice with PBS buffer and dissociated into single cells with 1 mL accutase solution (Sigma) per well at 37 °C. The dissociation was stopped by adding 1 mL FTDA medium per 6-well. The hiPSCs were resuspended in the media by gentle pipetting and centrifuged for 2 min at 200xg. $8x10^5$ hiPSCs were used in 100 µL nucleofector solution per electroporation reaction.

Single-stranded DNA oligonucleotide (ssODN) repair template oligos were resuspended in IDTE Buffer (IDT) to a stock concentration of 100 µM. 1 µL of ssODN repair template (100 µM) and 4 µL of RNP-complex were mixed with the hiPSC solution by gently pipetting, incubated for 5 min at room temperature and transferred to the nucleofection cuvette. Additionally, 1 µL Alt-R Cas9 Enhancer (100 µM, IDT) was added to the solution to promote transfection efficiency. The nucleofection cuvette was placed in the 4D-Nucleofector (Lonza) and hiPSCs were nucleofected by using the program CA137. After nucleofection, the cuvette was incubated for 5 min under cell culture conditions. Subsequently, hiPSCs were seeded in conditioned medium supplemented with Y-27632 (10 µM) and bFGF (30 ng/ml) (Supplementary Table 2) on a Matrigel-coated 24-well plate for 72 hours at 37 °C.

Subcloning and off-target analysis

72 hours after nucleofection, hiPSC were dissociated with Accutase and seeded in conditioned medium with Y-27632 (10 µM) and bFGF (30 ng/mL) at low seeding densities of 100, 250, 750, 1000 hiPSCs per well (10 cm^2) in a Matrigel-coated 6-well plate. In addition, the remaining nucleofected hiPSCs were seeded at a higher density of $5x10^5$ cells per well (10 cm^2) in a Matrigel-coated 6-well plate. Low-density hiPSC seedings were expanded for 9 to 10 days under daily conditioned medium change until clonal hiPSC colonies reached a size appropriate to pick. HiPSC cultures were incubated with conditioned medium with Y-27632 (10 µM) for 2 hours and sterile 100 µL-pipette tips were used to carefully scrape individual colonies from the 6-well plate and transfer them to Matrigel-coated 48-well plates into individual wells. 30 to 50 clones were picked per transfection approach and were sub-cultivated for 3-4 more days before they reached confluency and were splitted with a ratio of 1:2 into two 48-well copy plates. Colonies were again expanded with daily medium change until they reached confluency. One of the copy plates was used for cryo-preservation, while the second plate was used for DNA isolation. Cryopreservation was performed in 90% FBS and 10% DMSO.

QIAcube HT System (Qiagen) and QIAamp 96 DNA QIAcube HT kit (Qiagen) were used for DNA isolation according to the manufacturer’s instructions. Cryotubes of successfully edited hiPSC clones were thawed and expanded for master cell bank and working cell bank. Ten most likely off-targets were predicted (*in silico* tool IDT, Custom Alt R CRISPR-Cas9 gRNA software and CRISPOR software). Corresponding PCR primers were designed and PCR products were analysed by 1% (w/v) agarose electrophoresis and Sanger sequencing. Off-target primer sequences can be found in Supplementary Table 3, sheet 2.

Karyotyping

Karyotype analysis was performed using the nCounter Human Karyotype Panel (Nanostring Technologies) according to the manufacturer’s instructions with 250 µg DNA as starting material. The nCounter CNV Collector Tool software (Nanostring) was used for analysis.

Glucose- and lactate measurement

Glucose- and lactate concentrations were measured in EHT cell culture media by blood gas analysis. Samples were collected at baseline and after 24 hours of incubation in EHT medium on day 21 of culture and stored at -20°C. The blood gas analysis instrument ABL90 FLEX Analysator (Radiometer) was used to determine the metabolite concentrations by injecting the supernatant (0.5 mL) into the instrument with a 1 mL syringe.

Fatty acid- and carnitine supplementation

EHTs were cultured in serum-containing EHT culture medium until force values reached their plateau phase approximately at day 21-28 after generation. Then, EHTs were transferred to a serum- and glucose-free DMEM medium containing linoleic acid- and oleic acid-albumin (Sigma). The detailed serum-free fatty acid medium composition is shown in Supplementary Table 2. Contraction analysis was done daily after 2 hours of medium incubation for 4 days. Supplementation with carnitine was conducted by adding 2 mM L-carnitine hydrochloride (Sigma) to the EHT medium over the entire time of tissue cultivation.

Transmission electron microscopy

EHTs were transferred into a 24-well plate containing Tyrode's solution (Supplementary Table 2) with 1.8 mM Ca^{2+} and 30 mM butanedione monoxime (BDM) for 3-4 hours until EHTs completely stopped contraction. For fixation, EHTs were shifted into 4% paraformaldehyde (PFA, Thermo Scientific) in 0.1 M phosphate buffer containing 1% glutaraldehyde (Science Services) overnight at 4 °C. Samples were rinsed three times in 0.1 M sodium cacodylate buffer (pH 7.2–7.4) and osmicated using 1% osmium tetroxide in cacodylate buffer. Following osmication, the samples were dehydrated using ascending ethanol concentrations, followed by two rinses in propylene oxide. Infiltration of the embedding medium was performed by immersion in a 1:1 mixture of propylene oxide and Epon (Science Services, Germany), followed by neat Epon and hardening at 60 °C for 48 hours. For light microscopy, semi-thin sections (0.5 µm) with longitudinal orientation were mounted on glass slides and stained for 1 minute with 1% toluidine blue. For electron microscopy, ultra-thin sections (60 nm) were cut and mounted on copper grids and stained using uranyl acetate and lead citrate. Sections were examined and photographed using an EM902 (Zeiss) electron microscope equipped with a TRS 2K digital camera (A. Tröndle, Moorenweis, Germany).

Analysis of acylcarnitines and ceramides

EHT cells were homogenized in Milli-Q water (approximately 1.0x10^6 hiPSC-CM per 100 µl) using the Precellys 24 Homogenisator (Peqlab). The protein content of the homogenate was routinely determined using bicinchoninic acid. Levels of acylcarnitines and ceramides in the cell homogenates were determined by Liquid Chromatography coupled to Electrospray Ionization Tandem Mass Spectrometry (LC-ESI-MS/MS).

Acylcarnitines

Acylcarnitines were derivatized to butyl esters and using a procedure previously described (Giesbertz *et al*, 2015) with several modifications: To 100 µL of cell homogenate 750 µL of extraction solution (methanol (containing 0.005% 3,5-di-*tert.*-4-butylhydroxytoluol)/Milli-Q water/chloroform 4:1:1 (v/v/v)) and 20 µL of an internal standard mixture, containing deuterated acylcarnitines (Lyophilized Internal Standard MassChrom Amino Acids and Acylcarnitines from Dried Blood, Chromsystems, reconstituted in 2.5 ml, then 1:5 diluted), were added. After thorough mixing and centrifugation (16,100 RCF, 10 min, 4 °C), the supernatant was transferred to a new tube. The residue was re-extracted with 750 µL of extraction solution. The supernatants were pooled and dried under a stream of nitrogen. The evaporated extracts were treated with 200 µL of freshly prepared derivatization solution (*n*-butanol/acetyl chloride 95:5 (v/v)). After incubation for 20 min at 60 °C in a ThermoMixer (Eppendorf) at 800 rpm, the samples were again dried under nitrogen. After the addition of 100 µL of methanol/water 3:1 (v/v) and centrifugation (16,100 RCF, 10 min, 4 °C), 80 µL of supernatant were transferred to autoinjector vials. LC-ESI-MS/MS analysis was performed as previously described (Aravamudhan *et al*, 2021). The LC chromatogram peaks of butyl esters of endogenous acylcarnitines and internal standards were integrated using the MultiQuant 3.0.2 software (SCIEX). Endogenous acylcarnitine species were quantified by normalizing their peak areas to the peak area of the internal standards. These normalized peak areas were normalized to the protein content of the sample.

Ceramides

To 50 µL of cell homogenate 50 µL of Milli-Q water, 750 µL of methanol/chloroform 2:1 (v/v), and internal standard (127 pmol ceramide 12:0, Avanti Polar Lipids) were added. Lipid extraction and LC-ESI-MS/MS analysis were performed as previously described (Schwamb *et*

al, 2012). The LC chromatogram peaks of endogenous ceramide species and the internal standard ceramide 12:0 were integrated using the MultiQuant 3.0.2 software (SCIEX). Endogenous ceramide species were quantified by normalizing their peak areas to the peak area of the internal standard ceramide 12:0. These normalized peak areas were normalized to the protein content of the sample.

Actions potential

Actions potential (AP) measurements in EHT were performed with standard sharp microelectrode as described previously (Lemoine *et al*, 2018). The EHTs were transferred from the 24-well EHT culture plate into the AP measuring chamber by cutting the silicone posts and were fixed with needles in recording chamber. All measurements were done with tissues continuously superfused with Tyrode´s solution (NaCl 127 mM, KCl 5.4 mM, $MgCl_2$ 1.05 mM, $CaCl_2$ 1.8 mM, glucose 10 mM, $NaHCO_3$ 22 mM, $NaHPO_4$ 0.42 mM, balanced with O_2-CO_2 [95:5] at 36 °C, pH 7.4). Microelectrodes had a resistance between 25 - 55 MΩ when filled with 2 M KCl. The signals were amplified by a BA-1s npi amplifier (npi electronic GmbH, Tamm, Germany). APs were recorded and analyzed using the Lab-Chart software (version 5, AD Instruments Pty Ltd., Castle Hill NSW, Australia). Take-of potential (TOP) was defined as the diastolic membrane potential directly before the upstroke.

Analysis of mitochondrial respiration

The Seahorse™ XF96 extracellular flux analyzer was used to assess mitochondrial respiration as previously described (Mosqueira et al, 2019), using the Mito Stress Kit (Agilent Technologies). Briefly, cryopreserved isogenic sets of hiPSC-CMs were seeded into Matrigel™-coated (BD #356235) XF96 well plates at a density of approximately 5000 cells/mm^2. HiPSC-CMs were cultured for 2 days in RPMI1640 (USBiological Life Sciences #R9010-01) supplemented with B-27 with insulin (LifeTechnologies #0080085-SA), 2 mM L-glutamine (Life Technologies #25030-081), 10% Fetal Bovine Serum (Gibco #16000044) and 0.6 mM CaCl2. After 2 weeks, medium was exchanged for XF basal medium (Agilent Technologies #102353), supplemented with 10 mM glucose (Sigma #G7528), 1 mM sodium pyruvate (Sigma #S8636) and 2 mM L-glutamine (Life Technologies #25030-081) 1h before the conduction of the assay. Selective inhibitors were sequentially injected during the measurements (1.5 µM oligomycin, 0.4 µM FCCP, 1 µM rotenone; Agilent Technologies), following the manufacturer's

instructions. The measured Oxygen Consumption Rate (OCR) values were normalized to the number of cells in each well, quantified by 1:400 Hoechst33342 staining (Sigma #B2261) in PBS (Gibco #14190-094) using fluorescence at 355 nm excitation and 460 nm emission in an automated imaging platform (CellaVista, Synentec).

Tandem Mass Tag (TMT)-based quantitative proteomic analysis

EHT harvesting for quantitative mass spectrometry

EHTs from hiPSC-CMs were cultured in EHT medium for 28 days before proteome analysis. EHTs were washed twice in warm PBS buffer and incubated with collagenase II solution (collagenase II (200 units per mL), HBSS minus Ca^{2+}/Mg^{2+}, HEPES (10 mM), Y-27632 (10 µM), and BTS (30 µM)) in a falcon tube for 3 hours. Dissociated hiPSC-CMs were gently titrated with a 1000 µL-pipette (Eppendorf) until the last cluster of cells was disaggregated, spun down for 5 min at 200xg and supernatant was removed. The pellet was frozen in liquid nitrogen and stored at -80 °C before subjection to quantitative proteome analysis.

Tissue protein extraction and digestion for mass spectrometry

Cell pellets were lysed in 50 mM Tris, 0.1% SDS, pH=8.8, with protease inhibitors. After centrifugation at 4 °C at 16,000xg for 10 min, the supernatant was transferred to new 1.5 mL tubes and protein concentration was measured using a BCA protein assay kit (Thermo Fisher Scientific). For each sample, 23 µg of proteins were denatured by the addition of 9 M urea, 3 M thiourea (final conc. 6 M urea, 2 M thiourea) and reduced by the addition of 100 mM DTT (final conc. 10 mM) followed by incubation at 37 °C for 1 hour, 240 rpm. The samples were then alkylated by the addition of 500 mM iodoacetamide (final conc. 50 mM) followed by incubation in the dark for 1h at room temperature. Pre-chilled (-20 °C) acetone (1:9 volume ratio) was used to precipitate the samples overnight at -20 °C. Samples were centrifuged at 16,000xg for 30 min at 4 °C and the supernatant was subsequently discarded. Protein pellets were dried using a vacuum centrifuge (Thermo Fisher Scientific, Savant SPD131DDA), re-suspended in 0.1M TEAB buffer, pH 8.2 (Sigma T7408), and 0.6 µg trypsin was added. The digestion was performed overnight at 37 °C, 240 rpm, and stopped by adding 10% TFA (final conc. 1%). C18 clean-up was performed using Agilent Bravo AssayMAP and the eluted peptides were dried using a vacuum centrifuge.

Sample labeling for mass spectrometry

The samples were resuspended in 0.1 M TEAB and a pooled sample was made by taking the same amount of proteins from each individual sample. Samples were labeled with Tandem Mass Tag (TMT) 11-plex reagent (Thermo Fisher Scientific) following the manufacturer's instructions. The pooled sample labeled with TMT-126 was used as an internal standard. The samples labeled with different tags of the 11-plex TMT were combined, dried, and resuspended in 300 µl of 0.1% TEA. Samples were further fractionated using high pH RP HPLC (Agilent 300Extend-C18 3.5um 4.6x150mm P/N 763973-902) and 16 fractions were collected for each TMT mixture. All fractions were dried and resuspended in LC solution (2% acetonitrile (ACN), 0.05% TFA).

Mass spectrometry analysis

Samples were injected and separated by a nanoflow HPLC (Ultimate 3000 RSLCnano, Thermo Fisher Scientific) on an EASY-Spray column (C18, 75 µm x 50 cm, 2 µm) using 2 hour LC gradient: 0-10 min, 4%-10%B; 10-75 min, 10%-30%B; 75-80 min, 30%-40%B; 80-85 min, 40%-99%B; 85-90 min, 99%B; 90-120 min, 4%B; A=0.1% FA in H2O and B= 0.1% FA, 80% ACN in H2O. The flow rate was 250 nl/min and column temperature was set at 45°C. The separated peptides were directly injected to an Orbitrap Fusion Lumos MS (Thermo Fisher Scientific) and analyzed using a synchronous precursor selection (SPS)-based MS3 method for TMT-labeled sample. Full MS spectra were collected on the Orbitrap with a resolution of 120,000 and scan range 375-1500 m/z. The most abundant ions were fragmented using CID and MS2 spectra were collected on a linear ion trap, with dynamic exclusion enabled. The 5 most abundant ions from every MS2 spectrum were selected and fragmented at the same time using HCD with collision energy 65% and MS3 spectra were collected on the Orbitrap with a resolution of 60,000 and a scan range of 110-500 m/z to measure the TMT reporter ions. The cycle time was set at 3 seconds.

Raw data were analyzed using Proteome Discoverer 2.4. The 16 fractions of each TMT mixtures were loaded as fractions and analyzed together. Each TMT tag was assigned to the correct sample and group. Data were searched against the human UniProt/SwissProt database (version 2020_01, 20365 protein entries). Trypsin was used as an enzyme and maximum 2 missed cleavage was allowed. The precursor mass tolerance was set at 10 ppm and fragment mass tolerance was set at 0.8 Da. Carbamidomethylation on cysteine and TMT 6plex on N-

terminal and lysine were used as static modifications. Oxidation on methionine was used as a dynamic modification. Reporter ions S/N were used for quantification. The quantification values were normalized to total peptide amount and scaled on controls (pooled sample labeled with TMT-126). The scaled abundance was exported for further analysis.

The dataset was first imputed to replace missing relative quantities to zeros when these were consistent among any of the examined phenotypes. In specific, when the percent of missing values in one examined phenotype exceeded 90% and the percentage of missing values for the other phenotypes was below 10% then the missing values of the examined phenotype were imputed to zeros. The relative quantities of the proteins were then scaled using log2 transformation. Next, the dataset was filtered to keep only proteins with less than 30% missing values, without considering the previously imputed missing values as missing. The remaining missing values were imputed using KNN-Impute method with k equal to 3. The limma package has been used to compare different phenotypes using the Ebayes algorithm and correcting for selected covariates. The initial p-values were adjusted for multiple testing using Benjamini-Hochberg method and a threshold of 0.05 was used for the adjusted p-values to infer statistically significant changes.

Pathway enrichment analysis of significant proteins

The bioinformatic webtool Webgestalt (Liao *et al*, 2019) was used for pathway enrichment analysis of KEGG terms. KEGG pathways of significantly enriched proteins (fold change ≥ 1.4) were inferred by Benjamini-Hochberg testing with a p-value threshold of 0.05 and a maximum number of 150 proteins per category. To visualize the samples based on their proteomic profiles, principal component analysis (PCA) was conducted, and samples were projected in a 2D space based on their 2 most significant principal components. Samples were color-coded based on their phenotype. Volcano plots for the visualization of differentially expressed proteins were generated in GraphPad PRISM.

Gene expression analysis, mitochondrial DNA quantification

Total RNA was extracted from samples and complementary DNA (cDNA) was generated by reverse transcription using the TRIzol and high-capacity cDNA reverse transcription kit (Applied Biosystems) according to the manufacturer's instructions. All experiments were performed using technical triplicates. The ΔΔCt method was used for calculation of relative

transcript expression levels. Primer sequences are listed in Supplementary Table 3, sheet 3. Gene expression of target genes was normalized to the reference transcripts of the housekeeping gene glucuronidase-beta (GUSB). The qPCR experiments were conducted with the AbiPrism 7900HT Fast Real-Time PCR System (Applied Biosystems) using HOT FIREPol EvaGreen qPCR Mix Plus (Solis BioDyne).

Quantification of mitochondrial DNA (mtDNA) was performed according to a qPCR protocol recently described by Ulmer et al. (Ulmer *et al*, 2018). In brief, genomic DNA (including mitochondrial DNA) was isolated by TRIzol extraction according to the manufacturer's instruction. DNA concentrations of each sample was adjusted to 16.5 µg/µL prior to the experiment by dilution. The mt-DNA content was quantified by normalizing gene expression values of the mitochondrially encoded NADH dehydrogenase- 1 (mt-ND1) and -2 (mt-ND2) to the nuclear-encoded globular actin (g-actin). Primer sequences were used as described (Ulmer *et al*, 2018; Burkart *et al*, 2016) and are listed in Supplementary Table 3, sheet 3.

Southern blot

Southern blot probe design.

The 5'HR probe was cloned using PCR primer pairs SLC_5HR1 (Supplementary Table 3, sheet 4), and PCR amplified using pairs SLC_5HR2. The 3'HR probe was cloned using PCR primer pairs SLC_3HR1, and PCR amplified using pairs SLC_3HR2 (Supplementary Table 3, sheet 4).

The Southern blot procedure was performed according to Skryabin et al .(Skryabin *et al*, 2020). HiPSC were thawed from an MCB aliquot and expanded to T25 flask format with 100% confluency. HiPSCs were washed with 5 mL PBS per flask and lysed in 1 mL standard lysis buffer containing 100 mM Tris-HCl (pH 8.5), 5 mM EDTA, 0.2% SDS, 200 mM NaCl, 100 g/mL Proteinase K (Roche) incubated overnight at 37 °C. DNA was extracted by phenol, phenol/chloroform extraction, precipitated in isopropanol and washed in 80% ethanol. DNA samples were dissolved in TE buffer. Approximately 10-15 µg of genomic DNA was digested with *Eco*RI, and *Hind*III restriction endonucleases, fractionated on 0.8% agarose gels and transferred to GeneScreen nylon membranes (NEN DuPont, USA). The membranes were hybridized with a ^{32}P-labeled 0,5-kb 5'HR probe containing sequences 5' to the deleted region (5'HR probe, Supplementary Figure 3) and washed with SSPE buffer (0.09 M NaCl, 5 mM NaH_2PO4, and 0.5 mM EDTA [pH 7.7]) and 0.5% sodium dodecyl sulfate at 65°C. Similarly,

membranes were hybridized and washed with a ^{32}P-labeled 1,2-kb 3'HR probe containing sequences 3' to the deleted region (3'HR probe, Supplementary Figure 3).

Single-nucleus RNA sequencing

EHTs were washed with PBS, detached from the PDMS posts, frozen in liquid nitrogen, and stored at -150 °C. Single-nucleus RNA sequencing (snRNA seq) was performed according to Litviňuková et al. (Litviňuková *et al*, 2020). In brief, single nuclei were isolated from frozen EHTs and purified by fluorescent-activated cell sorting (FACS). Nuclei were further processed using the Chromium Controller (10X Genomics) according to the manufacturer's protocol with a targeted nuclei recovery of 5,000 per reaction. 3' gene expression libraries were prepared according to the manufacturer's instructions of v3 Chromium Single Cell Reagent Kits (10X Genomics).

Sequencing data analysis

Bcl files were converted to Fastq files by using bcl2fastq. Each sample was mapped to the human reference genome GRCh38 (release Ens84) using the CellRanger suite (v.3.0.1). Mapping quality was assessed using the cellranger summary statistics; Empty droplets were identified by Emptydrops, implemented in the CellRanger workflow, and subsequently removed, while doublets were identified and filtered using Solo (Bernstein *et al*, 2020).

Downstream analysis was performed using the Python Scanpy v1.5.1 toolkit. Single nuclei were filtered for counts (300 ≤ n_counts ≤20,000), genes (500 ≤ n_genes ≤5,500), mitochondrial genes (percent_mito ≤1%), ribosomal genes (percent_ribo ≤1%), and soft max score detected by Solo (solo_softmax_scores ≤0.5). After read count normalization and log-transformation, highly variable genes were selected. Principal components were computed, and elbow plots were used to define the appropriate number of principal components for neighbor graph construction. Prior to manifold construction using UMAP, selected principal components were harmonized by using Python Harmonypy. Finally, nuclei were clustered using the network-based Leiden algorithm.

Statistical analysis

GraphPad Prism software 8.4.3 was used to perform statistical analysis. All data was depicted as mean±SEM either as scatterplots or bar graphs. Where possible, data sets were tested for

normal distribution and the appropriate statistical test was chosen accordingly. Either the unpaired or nested Student's t-test, a nested-, a classical one-way ANOVA or a two-way ANOVA (plus Bonferroni's post-test) was used to determine whether the difference between groups was statistically significant. A p-value lower than 0.05 was statistically significant.

References

Aravamudhan S, Türk C, Bock T, Keufgens L, Nolte H, Lang F, Krishnan RK, König T, Hammerschmidt P, Schindler N, *et al* (2021) Phosphoproteomics of the developing heart identifies PERM1 - An outer mitochondrial membrane protein. *J Mol Cell Cardiol* 154: 41–59

Bernstein NJ, Fong NL, Lam I, Roy MA, Hendrickson DG & Kelley DR (2020) Solo: Doublet Identification in Single-Cell RNA-Seq via Semi-Supervised Deep Learning. *Cell Syst* 11: 95-101.e5

Burkart AM, Tan K, Warren L, Iovino S, Hughes KJ, Kahn CR & Patti ME (2016) Insulin Resistance in Human iPS Cells Reduces Mitochondrial Size and Function. *Sci Rep* 6: 1–12

Giesbertz P, Ecker J, Haag A, Spanier B & Daniel H (2015) An LC-MS/MS method to quantify acylcarnitine species including isomeric and odd-numbered forms in plasma and tissues. *J Lipid Res* 56: 2029–2039

Lemoine MD, Krause T, Koivumäki JT, Prondzynski M, Schulze ML, Girdauskas E, Willems S, Hansen A, Eschenhagen T & Christ T (2018) Human Induced Pluripotent Stem Cell-Derived Engineered Heart Tissue as a Sensitive Test System for QT Prolongation and Arrhythmic Triggers. *Circ Arrhythmia Electrophysiol* 11: 1–15

Liao Y, Wang J, Jaehnig EJ, Shi Z & Zhang B (2019) WebGestalt 2019: gene set analysis toolkit with revamped UIs and APIs. *Nucleic Acids Res* 47: W199–W205

Litviňuková M, Talavera-López C, Maatz H, Reichart D, Worth CL, Lindberg EL, Kanda M, Polanski K, Heinig M, Lee M, *et al* (2020) Cells of the adult human heart. *Nature* 588: 466–472

Schwamb J, Feldhaus V, Baumann M, Patz M, Brodesser S, Brinker R, Claasen J, Pallasch CP, Hallek M, Wendtner CM, *et al* (2012) B-cell receptor triggers drug sensitivity of primary

CLL cells by controlling glucosylation of ceramides. *Blood* 120: 3978–3985

Skryabin B V., Gubar L, Seeger B, Kaiser H, Stegemann A, Roth J, Meuth SG, Pavenstädt H, Sherwood J, Pap T, *et al* (2020) Pervasive head-to-tail insertions of DNA templates mask desired CRISPR-Cas9-mediated genome editing events. *Sci Adv* 6

Ulmer BM, Stoehr A, Schulze ML, Patel S, Gucek M, Mannhardt I, Funcke S, Murphy E, Eschenhagen T & Hansen A (2018) Contractile Work Contributes to Maturation of Energy Metabolism in hiPSC-Derived Cardiomyocytes. *Stem Cell Reports* 10: 834–847
